# Discovering consensus genomic regions in wheat for root-related traits by QTL meta-analysis

**DOI:** 10.1038/s41598-019-47038-2

**Published:** 2019-07-22

**Authors:** Jose Miguel Soriano, Fanny Alvaro

**Affiliations:** Sustainable Field Crops Programme, IRTA (Institute for Food and Agricultural Research and Technology), Lleida, Spain

**Keywords:** Agricultural genetics, Plant breeding

## Abstract

Root system architecture is crucial for wheat adaptation to drought stress, but phenotyping for root traits in breeding programmes is difficult and time-consuming owing to the belowground characteristics of the system. Identifying quantitative trait loci (QTLs) and linked molecular markers and using marker-assisted selection is an efficient way to increase selection efficiency and boost genetic gains in breeding programmes. Hundreds of QTLs have been identified for different root traits in the last few years. In the current study, consensus QTL regions were identified through QTL meta-analysis. First, a consensus map comprising 7352 markers was constructed. For the meta-analysis, 754 QTLs were retrieved from the literature and 634 of them were projected onto the consensus map. Meta-analysis grouped 557 QTLs in 94 consensus QTL regions, or meta-QTLs (MQTLs), and 18 QTLs remained as singletons. The recently published genome sequence of wheat was used to search for gene models within the MQTL peaks. As a result, gene models for 68 of the 94 *Root_MQTLs* were found, 35 of them related to root architecture and/or drought stress response. This work will facilitate QTL cloning and pyramiding to develop new cultivars with specific root architecture for coping with environmental constraints.

## Introduction

Wheat is the most widely cultivated crop in the world, providing humans with about 18% of their daily intake of calories and 20% of their protein (http://faostat.fao.org/). Because of its wide adaptability, wheat is grown in a wide range of environments, many of them in dryland regions, where soil water deficit is the main limiting factor for crop growth, and production depends on stored soil moisture. Moreover, as a consequence of human-induced climate change, warmer temperatures and lower and more erratic water availability affecting the major producing areas are expected in the next few decades^1^.

Breeding for adaptation to drought-prone environments is challenging because of its complexity and that of the plant mechanisms involved in drought tolerance^2^. Among the mechanisms adopted by plants, the root system architecture plays an important role in crop performance under low water input conditions^3^. Modelling studies conducted in Australia have shown that direct selection for deeper, more effective roots could considerably increase the capture of water and nitrogen from soil, thus resulting in a wheat yield improvement under rainfed conditions^4,5^. However, because of the inherent difficulties in measuring and assessing it, root system architecture has not been considered a selection trait in the breeding process^6^. Discovering quantitative trait loci (QTLs) controlling root architecture and identifying molecular markers linked to them are an essential tool for exploiting root traits in breeding programmes through marker-assisted selection.

A number of QTL studies for a wide range of root traits have been carried out in the last few years, and some of them are reviewed in Table 1. These studies identified hundreds of QTLs in different mapping populations with different types of markers. In order to identify consensus QTL regions in the genome, Goffinet and Gerber^7^ developed QTL meta-analysis. This method allows results of independent QTL studies to be integrated in a consensus or reference map. The power of QTL meta-analysis lies in identifying regions of the genome that are most frequently involved in trait variation and narrowing down the QTL supporting intervals, thus facilitating the identification of candidate genes for positional cloning. In selecting meta QTLs (MQTLs) to be used for breeding purposes, three criteria must be met^8^: (1) a small supporting interval, (2) clustering of a high number of initial QTLs, and (3) a high effect of the phenotypic variance explained by the initial QTLs.

**Table 1:** Summary of QTL studies included in the meta-analysis.

Reference	Cross	Type	Size	Traits^a^	N QTLs	Projected QTLs
An *et al*.^71^	Hanxuan 10 × Lumai 14	DH	120	RDW	4	3
Ayalew *et al*.^32^	W7984 × Opata 85	RIL	104	RDW, TRL	13	13
Bai *et al*.^72^	Avalon × Cadenza	DH	199	RDW, RSA, RSR, RV, SAL, SASA, SAVol, SLL, SLSA, SLVol, TRL	32	32
Ballesteros *et al*.^73^	USG3209 × Jaypee	RIL	130	TRB, TRL	7	6
Botwright Acuña *et al*.^74^	Cranbrook × Halberd	DH	161	AWSDW, BWSDW, RDW	21	18
Christopher *et al*.^29^	SeriM82 × Hartog	DH	184	RGA, TRN	11	4
Czyczyło-Mysza *et al*.^75^	Chinese spring × SQ1	DH	90	RDW, TRL	18	17
Ehdaie *et al*.^76^	Iran #49 × Yecora Rojo	RIL	168	DRW, LRL, RPR, RSR,SRW, TRL	12	6
Guo *et al*.^77^	Chuan 35050 × Shannong 483	RIL	131	ARN, RDW, RKC, RKUE, RNC, RPC, RPUE	13	13
Hamada *et al*.^78^	U24 × Ayahikari	DH	103	DRR, RAH, RER, SRN	7	7
Horn *et al*.^79^	Spark × Rialto	DH	119	RHL	3	3
Horn *et al*.^79^	Charger × Badger	DH	95	RHL	1	1
Ibrahim *et al*.^80^	Devon × Syn084	BC	177	ARD, CRS,FRK, RSA, RV, TIP, TRL	32	17
Iehisa *et al*.^81^	Ldn/KU-2159 × Ldn/IG126387	F_2_	100	RRGI, TRL	3	3
Iehisa *et al*.^82^	Chinese spring × Hope5A	F_2_	110	RRGI, TRL	2	2
Kabir *et al*.^83^	Nongda 338 × Jingdong 6	DH	216	MRL, RSA, RV, TIP, TRL	39	26
Kabir *et al*.^83^	Nongda 331 × Zang 1817	RIL	217	MRL, RSA, RV, TIP, TRL	14	9
Kadam *et al*.^84^	WL711 × C306	RIL	206	MaxRL, RBB30, RBU30, TRB	5	5
bKubo *et al*.^21^	Jennah Khetifa × Cham1	RIL	110	RDW, RP	2	2
Landjeva *et al*.^85^	Chinese Spring × Synthetic 6×	RIL	85	RSR	5	5
Li *et al*.^86^	Rio Blanco × IDO444	RIL	159	LRL, TRL, TRN	15	14
Liu *et al*.^87^	Hanxuan 10 × Lumai 14	DH	150	MaxRL, PRA, SRA, SRN, TRL	46	42
^b^Maccaferri *et al*.^22^	Colosseo × Lloyd	RIL	176	ARL, LRN, PRD, PRL, RDW, RGA, RSR, RT6, RV, SRA, TRD, TRL, TRN	41	41
^b^Maccaferri *et al*.^22^	Meridiano × Claudio	RIL	181	ARL, LRN, PRD, PRL, PRS, PRV, RDW, RGA, RT6, RV, SRA, TRD, TRL, TRN	67	66
^b^Petrarulo *et al*.^23^	Creso × Pedroso	RIL	123	RV, SRA, TIP, TRL	57	57
Ren *et al*.^88^	Xiaoyan 54 × Jing 411	RIL	142	LatRL, MaxRL, RDW	35	31
Ren *et al*.^89^	Xiaoyan 54 × Jing 411	RIL	142	MaxRL, RDW	17	15
Sun *et al*.^90^	Chuan 35050 × Shannong 483	RIL	131	REW, RFW, RSR	31	26
Xie *et al*.^91^	Forno × Oberkulmer	RIL	226	MasRD, MaxRW, SRL, SRN	38	25
Yu and Chen^92^	W7984 × Opata85	RIL	112	RDW	8	8
Zhang *et al*.^93^	Weimai 8 xLuohan 2	RIL	229	LRL, RDW, RFW, RSR, TRN	38	27
Zhang *et al*.^93^	Weimai 8 × Yannong 19	RIL	302	LRL, RDW, RFW, RSR, TRN	17	8
Zhang *et al*.^94^	Weimai 8 × Jimai 20	RIL	172	LRL, RDW, RSR, TRN	17	15
Zhang *et al*.^94^	Weimai 8 × Luohan 2	RIL	179	LRL, RDW, RSR, TRN	20	17
Zhang *et al*.^95^	Weimai 8 × Yannong 19	RIL	175	LRL, RDW, RSR, TRN	19	15
Zhao *et al*.^95^	Huapei 3 × Yumai 57	DH	168	ACT, ARD, CCR, KCR, MCR, NCR, RDW, RSR, RV, SRA, TRL	44	35

^a^Abbreviation list for the traits is shown in Supplementary Material 2.

^b^Studies carried out in durum wheat.

QTL meta-analysis has been performed in the last few years in wheat for traits such as grain yield^9,10^, crop phenology^10,11^, disease resistance^8,12–14^, plant height^15^, grain-related traits^16,17^ and sprouting tolerance and dormancy^18^. QTL meta-analysis for root-related traits was performed previously by Darzi-Ramandi *et al*.^6^, who only considered 53 QTLs from chromosome groups 2 and 3 for the analysis, and by Iannucci *et al*.^19^, who performed the analysis in durum wheat using 100 QTLs retrieved from the literature together with 17 QTLs discovered in their study.

The present study reports the results of the largest QTL meta-analysis conducted for root traits in wheat. QTL projection was carried out on a consensus map also developed in this study by integrating three maps used as a reference composed mainly of simple sequence repeat (SSR) and DArT markers (Composite-2004, available at http://wheat.pw.usda.gov; SSR-2004^20^; and Integrated-2013^13^). The main objective of the study was to produce a repository of root QTL information to define consensus regions controlling root architecture in wheat.

## Results

### Consensus map

The Wheat_Consensus_2018 map included 7352 markers (Supplementary Material 1) after combining the Composite_2004, SSR_2004^20^ and Integrated_2013^13^ maps. The total length of the consensus map was 4994.0 cM, with an average chromosome length of 237.8 cM and a range of 155.6 cM (5D) to 347.5 cM (7A). The average number of markers per chromosome was 350, chromosome 3B carrying the highest number of markers (580) and chromosome 4D the lowest (178). The overall marker density was 1.5 markers/cM, ranging from 3.1 markers/cM on chromosome 3B to 0.8 markers/cM on chromosome 7D. Genome A covered a distance of 1810.2 cM with 2497 markers, genome B covered a distance of 1789.1 cM with 3230 markers, showing the highest marker density (1.8 markers/cM), and genome D covered a distance of 1394.7 cM with 1625 markers.

**Figure 1:**
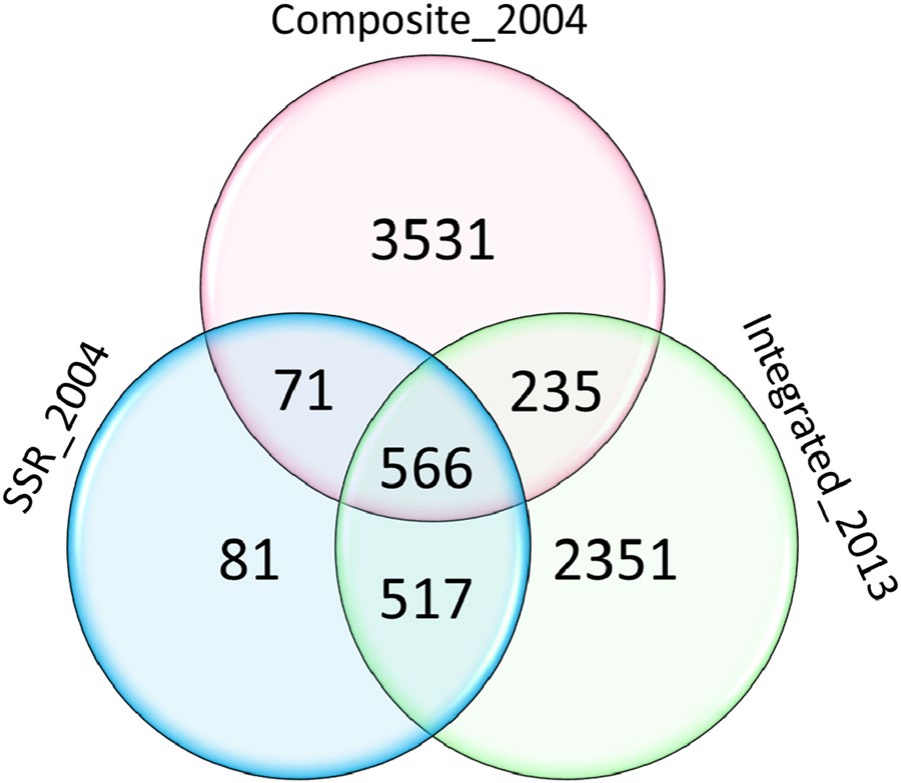
Venn diagram showing the number of unique and common markers among the three maps used to construct the consensus map.

The three maps shared 8% of the markers (566), whereas Composite_2004 had 3531 unique markers (48%), SSR_2004 81 had markers (1%) and Integrated_2013 had 2351 markers (32%). Composite_2004 and SSR_2004 shared 71 markers (1%), Composite_2004 and Integrated_2013 shared 235 markers (3%) and SSR_2004 and Integrated_2013 517 shared 517 markers (7%) (Fig. 1).

### QTL distribution and projection

**Figure 2:**
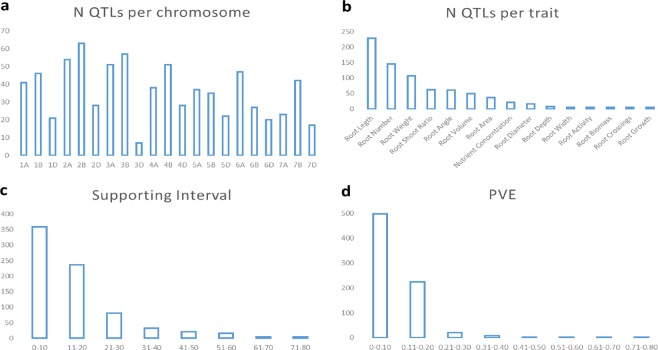
Traits estimated from the collected QTL studies. Number of QTLs per (**a**) chromosome, (**b**) trait, (**c**) supporting interval and (**d**) phenotypic variance explained (PVE).

Thirty studies published from 2006 to 2017 based on bi-parental populations reporting 754 QTLs for root traits were collected (Table 1, Supplementary Material 2). The studies covered 31 different populations with 56 lines as parents. QTLs were distributed throughout the whole genome. The number of QTLs per chromosome ranged from 7 on chromosome 3D to 63 on chromosome 2B, with an average of 36 QTLs per chromosome. Of the QTLs, 39% were identified in genome A, 42% in genome B and 19% in genome D (Fig. 2a). Three studies^21–23^ were carried out in durum wheat, where only genomes A and B are present, reporting a total of 167 QTLS. The most reported QTLs corresponded to traits involved in root length (30%), followed by QTLs involved in root number and weight (19% and 14% respectively) (Fig. 2b). Supporting intervals (SI) ranged from 0 to 75.1 cM, with an average of 14.8 cM. Approximately half of the collected QTLs (47%) had an SI lower than 10 cM, and 79% had an SI lower than 20 cM (Fig. 2c). The proportion of phenotypic variance explained (PVE) by single QTLs followed an L-shaped distribution, with most of the QTLs showing a PVE lower than 0.20 (96%) (Fig. 2d). PVE ranged from 0.01 to 0.76, with an average of 0.1.

**Figure 3:**
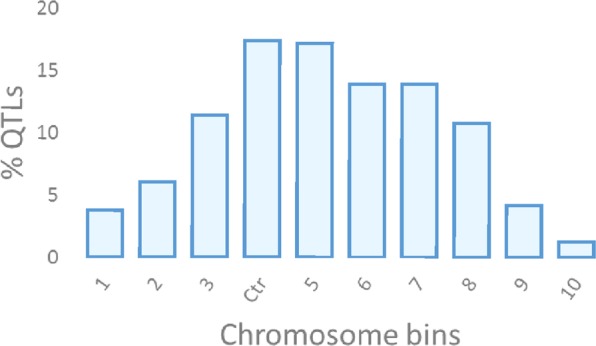
Representation of the QTL coverage along a consensus chromosome Ctr: centromere (bin 4). Each bin represents 10% of the whole chromosome.

**Figure 4:**
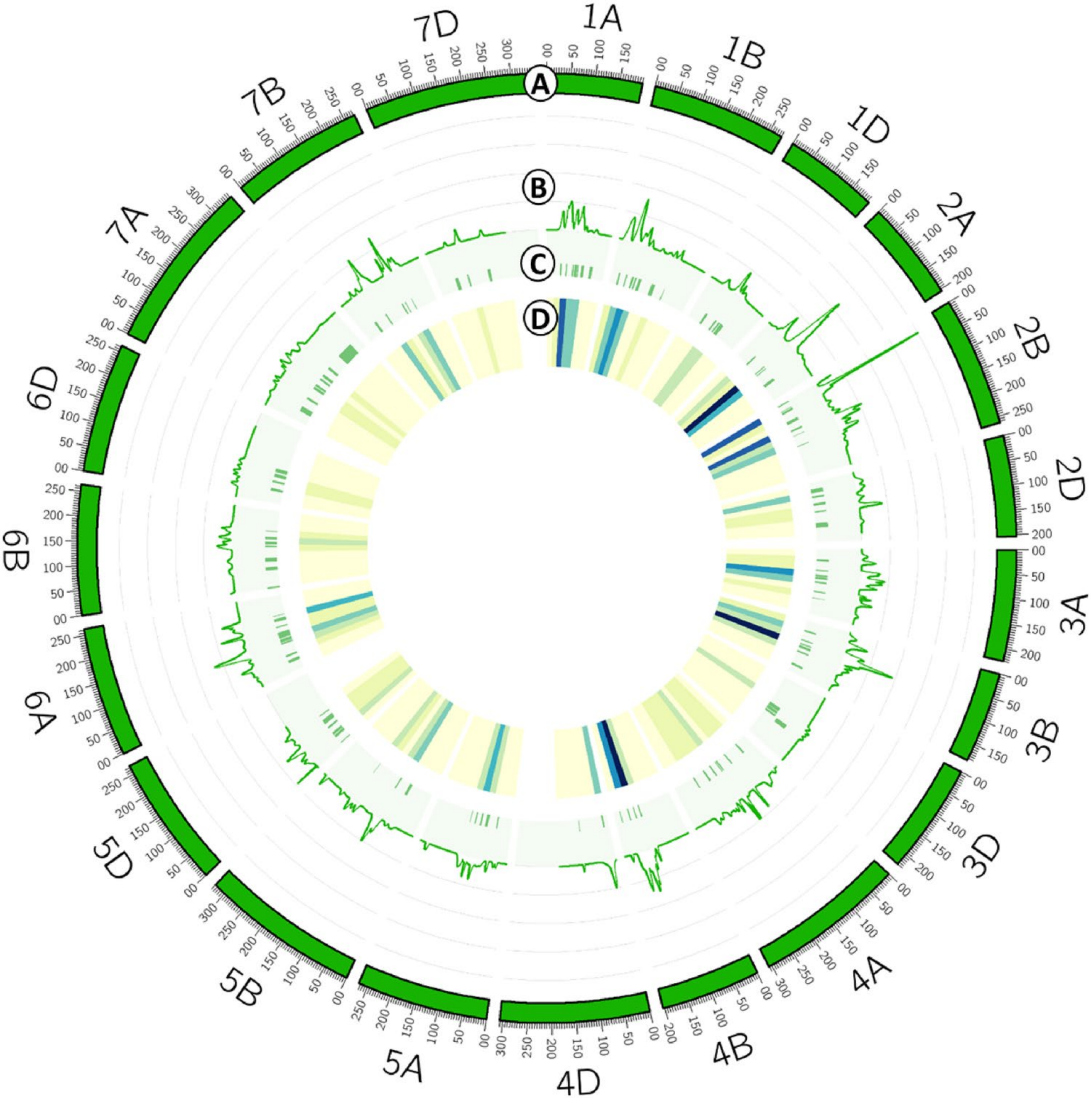
Concentric circles representing the root QTLome. (**A**) Wheat_Consensus_2018 map. (**B**) Frequency of QTLs computed as QTL-overview index. (**C**) MQTL positions with an SI of 95%. (**D**) Heatmap representing the number of QTLs for 25 cM bins (number of QTLs increasing from light to dark).

A total of 634 out of the 754 collected QTLs were projected onto the Wheat_Consensus_2018 map. One hundred and twenty-one QTLs were not projected because (1) they lacked common markers between initial and consensus maps, and (2) the QTL showed a low PVE, causing a large SI (>50 cM). The projection onto the consensus map showed a clustering of QTLs in the centromeric and pericentromeric regions (Figs 3 and 4).

Figure 3 represents a consensus chromosome among the 21 wheat chromosomes divided into 10 bins. Each bin represents 10% of the total length of the consensus chromosome. Centromeric (4) and pericentromeric bins (3 and 5) included almost half of the QTLs (46%), decreasing towards telomeres. Central regions of chromosome arms, bin 2 (short arm) and bins 6 to 8 (long arm), carried 45% of the QTLs and, finally, the telomeric regions, bin 1 (short arm) and bins 9 and 10 (long arm), contained only 9% of the QTLs.

To detect the genome regions most frequently involved in the genetic control of root traits, the QTL-overview index^24^ was calculated for each cM of the consensus map (Fig. 4, Supplementary Material 3). A total of 150 overview peaks were obtained, of which 126 exceeded the average value of the statistic for each chromosome and represent hot spots for root QTLs. Additionally, 41 of the 126 peaks exceeded a high value threshold calculated as five times the average value of the overview index for each chromosome (Supplementary Material 3). These 41 peaks corresponded to 35 of the 94 MQTL (37%) and included 292 QTLs (52%).

### QTL meta-analysis

From 634 QTLs projected onto the Wheat_Consensus_2018 map 557, were grouped into 94 meta-QTLs (MQTLs), whereas 18 remained as single QTLs (sQTLs) not overlapping with MQTLs (Table 2, Fig. 4). Fifty-nine QTLs were not assigned to any MQTL because of their large SI overlapping with different MQTLs or because the predicted QTL peaks were not included within any MQTL SI. However, they were not considered as sQTLs as their SI overlapped with MQTLs.

**Table 2:** Summary of the meta-QTLs for root traits including the gene model based on the wheat genome sequence annotation for the closest marker and its protein description.

MQTL	Chr	Position (cM)	SI (95%)	N QTLs	N studies	Traits	Closest marker	Gene model^a^	Description
*Root_MQTL_1*	1A	48.0	4.5	5	2	RDW, PRV,LRL, RV	wPt-1862_1A	TraesCS1A01G038700	NBS-LRR like resistance protein
*Root_MQTL_2*		66.3	2.9	16	3	TRL, RHL, SRA, TIP, LatRL, RV, MaxRL	Xgwm33_1A	…	…
*Root_MQTL_3*		89.2	3.2	5	3	RDW, RFW, CCR, TRL	Xwmc286_1A	TraesCS1A01G069600	Zinc finger CCCH domain-containing protein
*Root_MQTL_4*		99.2	8.3	2	2	RDW, TRL	Xgwm135_1A	TraesCS1A01G228000	Phosphatase 2C family protein
*Root_MQTL_5*		106.2	2.7	7	5	RAH, NCR, RSR, LRL, RV, RDW, TRL	Xpsr1327_1A	TraesCS1A01G431000	F-box family protein
*Root_MQTL_6*		121.3	7.1	2	1	TRL	XksuE3_1A	…	…
*Root_MQTL_7*	1B	27.2	3.5	12	6	RV, RSR, TRL, BWSDW, TIP, SRA, AWSDW, RDW	Xwmc619_1B	TraesCS1B01G086300	Glycosyl hydrolase family protein
*Root_MQTL_8*		51.1	2.8	15	4	ACT, TRL, SRA, ARN, RPUE, RPC, RNC, RSR, RKC, RKUE, RV, TRN	Xwmc500.2_1B	…	…
*Root_MQTL_9*		73.8	4.8	4	3	RDW, TRL, TIP, MaxRL	Xpsr949_1B	TraesCS1B01G144600	Hexosyltransferase
*Root_MQTL_10*		84.2	3.5	5	4	TRL, ARL, LRL, RDW, RV	Xbarc240_1B	TraesCS1B01G214900	Peroxidase
*Root_MQTL_11*		99.3	7.9	2	1	LRL	Xwmc85a_1B	TraesCS1B01G312100	Glycosyltransferase
*Root_MQTL_12*		147.4	10.6	2	2	DRR, TRL	wPt-8832_1B	TraesCS1B01G440200	Root Primordium Defective 1
*Root_MQTL_13*		187.1	1.1	3	2	TRB, RSR	wPt-6142_1B	…	…
*Root_MQTL_14*	1D	50.0	6.4	3	3	AWSDW, SRA, RFW	P32/M59-2_1D	TraesCS1D01G040100	Poly [ADP-ribose] polymerase
*Root_MQTL_15*		87.7	3.6	7	3	RDW, RSR, BWSDW	Xwmc590_1D	TraesCS1D01G238300	Signal peptidase I
*Root_MQTL_16*		100.8	4.5	2	2	FRK, RFW	Xbarc62_1D	TraesCS1D01G451200	Early flowering 3
*Root_MQTL_17*	2A	47.0	3.5	11	5	TRN, LRN, LRL, TRL, TIP, RDW, RSA	Xbarc1138_2A	TraesCS2A01G004600	C2H2-like zinc finger protein
*Root_MQTL_18*		91.7	2.2	14	4	MaxRW, RSR, RV, TRL, RPR, SRA, TIP, SRW, RT6	Xwmc296_2A	TraesCS2A01G134400	Plasma-membrane choline transporter family protein
*Root_MQTL_19*		99.6	1.9	8	6	TRL, RHL, RV, SRN,MRL, TRN	Xbarc309_2A	TraesCS2A01G160000	Pentatricopeptide repeat-containing protein
*Root_MQTL_20*		144.4	9.2	3	2	LRN, RV, RDW	Xcfd168_2A	…	…
*Root_MQTL_21*	2B	1.2	3.3	2	1	TRN	Lr16_2B	…	…
*Root_MQTL_22*		22.3	1.0	14	5	RAH, MaxRL, PRL, RSR, TRL, LRL, RFW, PRE, LatRL	wPt-8737_2B	TraesCS2B01G029100	Glycosyltransferase
*Root_MQTL_23*		30.0	6.4	4	1	TRL, SRA, PRL, ARL	Xcdo456_2B	TraesCS2B01G040500	Glycosyltransferase
*Root_MQTL_24*		97.5	5.8	3	2	TRL, SRN	Xwmc770_2B		
*Root_MQTL_25*		116.5	1.9	18	7	RDW, RGA, BWSDW, PRA, SRA, TRL, TRN	Xbarc55_2B	TraesCS2B01G159800	Terpene synthase
*Root_MQTL_26*		154.3	0.5	17	8	RGA, RHL, RFW, RDW, RT6, ARL, PRL, CRS, PRD, TRN, SRA	wPt-6522_2B	TraesCS2B01G436400	Oleosin
*Root_MQTL_27*	2D	29.2	12.4	5	1	SASA, RSA, RDW, SAVol, RV	Xgwm296_2D	TraesCS2D01G100800	NAC domain protein,
*Root_MQTL_28*		49.2	5.0	4	4	MaxRL, RSR, TRB, TRL	Xcfd255_2D	TraesCS2D01G118300	E3 ubiquitin-protein ligase
*Root_MQTL_29*		75.2	7.8	2	2	RDW, RSR	Xwmc18_2D	TraesCS2D01G185600	Dihydroflavonol-4-reductase
*Root_MQTL_30*		100.5	2.8	7	3	RSR, TRN, RDW	Xbarc228_2D	TraesCS2D01G464800	Multidrug resistance protein ABC transporter family protein
*Root_MQTL_31*	3A	37.7	4.6	6	3	TRD, RDW, PRD	Xbcd1428_3A	TraesCS3A01G025200	E3 ubiquitin-protein ligase
*Root_MQTL_32*		72.3	2.9	7	4	TRL, MaxRD, TRD, RSA, SRL, TIP	Xgwm2_3A	TraesCS3A01G197700	Serine/threonine-protein phosphatase
*Root_MQTL_33*		90.7	5.5	7	2	RSA, TRL, SLL, SLVol, SLSA, TIP	Xwmc695_3A	…	…
*Root_MQTL_34*		99.2	5.7	6	3	TIP, SRA, TRN, PRE, RV, TRL	Xwmc264_3A	TraesCS3A01G376500	E3 ubiquitin-protein ligase SINA-like 10
*Root_MQTL_35*		113.8	4.5	5	3	RDW, RV, TRL, SRA	wPt-4725_3A	TraesCS3A01G477800	glutamyl-tRNA amidotransferase
*Root_MQTL_36*		146.7	3.4	2	2	RDW, RSR	wPt-1864_3A	…	…
*Root_MQTL_37*		169.6	1.3	4	3	LRL, TRN, RT6, TRL	wPt-9422_3A	TraesCS3A01G512900	Regulator of chromosome condensation (RCC1) family with FYVE zinc finger domain
*Root_MQTL_38*	3B	35.3	4.3	11	4	RSR, RDW, TRD, ARD, LRL, PRS, RV	Xwmc597_3B	…	…
*Root_MQTL_39*		68.3	3.6	5	4	MaxRD, TRL, RSR	wPt-9170_3B	TraesCS3B01G306100	transmembrane protein (DUF616)
*Root_MQTL_40*		82.6	2.4	11	2	SRN, TRL, SRA, PRA	Xwmc527a_3B	TraesCS3B01G334000	F-box family protein
*Root_MQTL_41*		92.4	6.3	2	1	TRL, MRL	Xwmc787_3B	TraesCS3B01G463600	F-box domain containing protein
*Root_MQTL_42*		109.7	3.7	8	5	AWSDW, BWSDW, RGA, TRD, RSR, RDW, TRN	wPt-10071_3B	TraesCS3B01G504900	B3 domain-containing protein
*Root_MQTL_43*		158.0	5.9	3	3	LRN, RDW, TRN	Xfba167b_3B	TraesCS3B01G586400	Late embryogenesis abundant protein
*Root_MQTL_44*	4A	40.7	5.1	8	5	RV, TIP, TRL, PRA, SRA, LRL	Xwmc89_4A	TraesCS4A01G216800	Late embryogenesis abundant hydroxyproline-rich glycoprotein
*Root_MQTL_45*		94.5	2.7	3	1	SRN, MaxRD, TRL	Xwmc513_4A	TraesCS4A01G306600	Germin-like protein
*Root_MQTL_46*		130.6	3.1	5	3	MaxRW, TRL, SRN, RDW, RNC	Xpsr115_4A	…	…
*Root_MQTL_47*		136.2	2.3	3	2	RPC, RKC, RSR	XS25M49(350)_4A	…	…
*Root_MQTL_48*		160.2	3.7	4	3	RFW, KCR, RDW	XgbxG557_4A	TraesCS4A01G372100	ABC transporter family protein
*Root_MQTL_49*		187.6	5.0	6	4	TRN, BWSDW, RDW, LRL	wPt-5543_4A	TraesCS4A01G431700	Late embryogenesis abundant hydroxyproline-rich glycoprotein
*Root_MQTL_50*		229.5	2.1	5	2	TRN, RDW, RSR, CCR, KCR	P31/M53-4_4A	…	…
*Root_MQTL_51*	4B	114.8	2.3	5	2	TRL, LRL, LRN	Xcdo795_4B	…	…
*Root_MQTL_52*		133.0	2.1	18	9	TIP, RDW, MaxRL, LRL, RBU30, ACT, TRB, RSR, RV, ARN, RBB30, TRL	QPhs.ocs-4B.1_4B	TraesCS4B01G136500	Root Hair Defective 3 homolog
*Root_MQTL_53*		153.4	2.5	16	4	RV, TRL, LRL, SRA, RT6, PRA, PRS, PRL, RT6, RDW, TRN, RGA, LRN	Xwmc413_4B	TraesCS4B01G347600	Peroxidase
*Root_MQTL_54*	4D	16.3	3.9	12	2	SAL, TIP, RSA, SASA, RDW, RSR, RSV, RV, TRL, MRL	Xgpw2180_4D	…	…
*Root_MQTL_55*	5A	48.5	3.9	4	1	MaxRD, TRL, MaxRW, SRN	Xwmc51_5A	TraesCS5A01G012600	ERD (Early-responsive to dehydration stress)
*Root_MQTL_56*		84.3	8.1	4	3	MRL, MaxRL, TRL, SRN	Xcfd17b_5A	TraesCS5A01G055000	Root Hair Defective 3 homolog
*Root_MQTL_57*		103.9	3.4	7	5	SAVol, TRN, AWSDW, TRN, SRN, RT6, PRL	Xbarc180_5A	TraesCS5A01G163400	Cinnamoyl-CoA reductase
*Root_MQTL_58*		123.6	3.0	4	3	RRGI, TRL, MaxRL, RSA	Xcdo1090A_5A	…	…
*Root_MQTL_59*		141.7	1.5	6	4	MRL, RDW, TRL, RSR, LRN	Xbarc40_5A	TraesCS5A01G228300	E3 ubiquitin-protein ligase
*Root_MQTL_60*	5B	80.6	8.1	3	2	TRN, ARL, RGA	Xgdm146_5B	…	…
*Root_MQTL_61*		122.4	3.2	16	7	TRL, SLVol, RV, SLL, RDW, SRA, MaxRL, SLSA, MRL	Xbarc109_5B	TraesCS5B01G127200	Pentatricopeptide repeat-containing protein
*Root_MQTL_62*		211.9	0.7	11	6	RV, PARA, TRL, ARD, MaxRD, TRN, LRL	Xbarc232_5B	TraesCS5B01G447800	Cytosine-specific methyltransferase
*Root_MQTL_63*	5D	35.7	8.0	3	2	MaxRL	Xcfd78_5D	TraesCS5D01G096700	Ribosomal RNA apurinic site specific lyase
*Root_MQTL_64*		47.2	8.1	2	2	TRL, RER	Xwmc805_5D	TraesCS5D01G176000	Late embryogenesis abundant hydroxyproline-rich glycoprotein
*Root_MQTL_65*		73.2	5.1	7	3	TRL, RSR, RDW	XgbxG083_5D	TraesCS5D01G342500	ALWAYS EARLY 2
*Root_MQTL_66*		121.6	2.3	3	3	RV, CCR, RDW	Xcfd86_5D	TraesCS5D01G504400	E3 ubiquitin-protein ligase
*Root_MQTL_67*	6A	35.2	8.6	3	3	RGA, RDW	Xgwm334_6A	…	…
*Root_MQTL_68*		55.9	4.9	5	3	TRN, NCR, RDW	wPt-7565_6A	TraesCS6A01G023100	Acid beta-fructofuranosidase
*Root_MQTL_69*		100.1	4.0	14	5	TRL, RFW, RHL, SRA, RV, RT6	Xbarc171_6A	TraesCS6A01G210900	Root meristem growth factor 1
*Root_MQTL_70*		110.0	10.4	3	3	TIP, MRL	Xbarc107_6A	TraesCS6A01G269500	Protein BREVIS RADIX
*Root_MQTL_71*		128.4	19.7	3	1	TRL, SAL, SLVol	Xcdo1373_6A	…	…
*Root_MQTL_72*		157.5	2.8	4	3	RDW, TRL LatRL, TIP	Xfba111b_6A	…	…
*Root_MQTL_73*		180.7	8.9	4	3	PRV, RDW, TRL, SRA, ARL	Xgwm169_6A	TraesCS6A01G393200	Heavy metal transport / detoxification superfamily
*Root_MQTL_74*		195.7	0.9	6	2	TRL, PRL, RP, PRS, RGA	Xcdo836_6A	TraesCS6A01G410100	Embryogenesis transmembrane protein-like
*Root_MQTL_75*	6B	66.7	9.3	2	2	LRN, TRL	Gli-B2_6B	TraesCS6B01G054600	F-box family protein
*Root_MQTL_76*		97.7	12.8	2	1	TIP, LatRL	TC85307_6B	TraesCS6B01G075800	SAUR-like auxin-responsive protein family, putative
*Root_MQTL_77*		141.8	4.8	5	3	TRN, ARL, RT6, RDW	Xbarc198_6B	TraesCS6B01G212200	Pentatricopeptide repeat-containing protein
*Root_MQTL_78*		153.8	7.4	3	1	PRS, TRD, PRL	Yr36_6B	…	…
*Root_MQTL_79*		170.4	5.8	4	2	TRN, MaxRL, PRL	Xfba67b_6B	TraesCS6B01G356400	E3 ubiquitin-protein ligase COP1
*Root_MQTL_80*		193.8	1.4	4	3	TIP, TRL, RDW	P32/M54-2_6B	TraesCS6B01G398300	Embryogenesis transmembrane protein-like
*Root_MQTL_81*	6D	42.9	7.5	2	2	RSA, TRL	XksuG48_6D	…	…
*Root_MQTL_82*		108.7	12.1	2	2	TRL	P43/M62-1_6D	TraesCS6D01G245400	Protein BREVIS RADIX
*Root_MQTL_83*	7A	82.5	6.1	2	2	ARN, RSR	Xgbx3480a_7A	…	…
*Root_MQTL_84*		135.0	6.1	3	1	PRV, SRA, PRD	Xgwm900_7A	TraesCS7A01G154600	Gibberellin receptor GID1A
*Root_MQTL_85*		159.9	6.2	3	2	RDW, RSR	Xbarc195_7A	TraesCS7A01G428400	Peroxidase
*Root_MQTL_86*		175.2	7.6	4	3	RGA, SRN, RDW	Xwmc9_7A	…	…
*Root_MQTL_87*		206.4	7.9	3	2	RDW, TRN	Xcfa2257_7A	TraesCS7A01G481200	F-box protein
*Root_MQTL_88*	7B	51.0	5.2	2	2	LRL, TRD	wPt-2278_7B	TraesCS7B01G184000	1-phosphatidylinositol-3-phosphate 5-kinase-like
*Root_MQTL_89*		95.7	2.6	15	8	LRL, RT6, TRN, TRL, PRL, MaxRL	wPt-7925_7B	TraesCS7B01G134800	Root hair defective 3 GTP-binding protein (RHD3)
*Root_MQTL_90*		160.5	2.4	2	2	ARD, MaxRL	wPt-5280_7B	TraesCS7B01G429700	Glycosyltransferase
*Root_MQTL_91*		173.3	2.0	8	3	ARD, RSR, TRL, KCR, MCR, SRN, MaxRW	Psy-B1_7B	TraesCS7B01G482000	Phytoene synthase
*Root_MQTL_92*		196.1	1.2	8	3	SRA, ARL, PRL, TRL, TRN, PRS, LRL	Xwmc273_7B	TraesCS7B01G472200	NAC domain-containing protein
*Root_MQTL_93*	7D	102.9	4.8	3	1	TRL, SRA, PRA	Stb5_7D	…	…
*Root_MQTL_94*		176.8	8.8	2	2	RDW	D1.1_ctg10053_7D	…	…

^a^ …: closest or flanking markers not mapped on the genome sequence.

The number of MQTLs per chromosome ranged from 1 on chromosome 4D to 8 on chromosome 6A. The QTLs on chromosome 3D did not cluster into MQTLs. The number of clustered QTLs per MQTL ranged from 2 on several chromosomes to 18 on chromosomes 2B (*Root_MQTL25*) and 4B (*Root_MQTL52*). Fifty-two MQTLs (55%) were derived from the clustering of QTLs from three or more different studies, thus involving different mapping populations. These MQTLs were more likely to be stable across different environments. The number of traits involved per MQTL ranged from 1 to 13 in *Root_MQTL53*. The SI of the MQTLs ranged from 0.47 to 19.68 cM with an average of 4.96 cM, showing a significant reduction from the initial QTLs ranging from 0.00 to 75.10 cM with an average of 14.80 cM.

Based on the criteria defined by Löffler *et al*.^8^ to consider a MQTL for marker assisted breeding, i.e: small supporting intervals of the MQTLs, high number of initial QTLs and high PVE of the initial QTLs, eight root MQTLs, the “Breeding MQTLs”, were selected (Table 3). Firstly, seventeen MQTLs including more than 10 QTLs with SI lower than 5 cM were identified. Subsequently those with a PVE mean of the initial QTLs of 0.10 or higher were chosen.

**Table 3:** Breeding MQTLs.

MQTL	Chr	Position (cM)	CI (95%)	Marker interval	N QTLs	Mean PVE	Trait category
*Root_MQTL_2*	1A	66.3	2.9	Xcfd15 - Xwmc104	16	0.11	Angle, Length, Number, Volume
*Root_MQTL_8*	1B	51.1	2.8	Xwmc500 - XKsu136	15	0.11	Nutrient Concentration, Activity, Angle, Length, Number, Volume, Root Shoot Ratio
*Root_MQTL_18*	2A	91.7	2.2	Xwmc32 - Xgwm339	14	0.13	Activity, Angle, Biomass, Length, Number, Root Shoot Ratio, Volume, Width
*Root_MQTL_22*	2B	22.3	1.0	wPt-3459 - Xwmc382	14	0.27	Angle, Length, Root Shoot Ratio, Weight
*Root_MQTL_25*	2B	116.5	1.9	Xcfa2043 - Xwmc272	18	0.10	Angle, Area, Length, Number, Weight
*Root_MQTL_40*	3B	82.6	2.4	Xcdo1164 - Xfbb110	11	0.12	Angle, Area, Length, Number
*Root_MQTL_52*	4B	133.0	2.1	Xgwm710 - Xgwm368	18	0.10	Activity, Angle, Biomass, Length, Number, Root Shoot Ratio, Volume, Weight
*Root_MQTL_69*	6A	100.1	4.0	Xbarc146 - Xcdo29	14	0.10	Angle, Length, Number, Volume, Weight

### Gene models

Identification of gene models using the Gbrowse tool available at https://wheat-urgi.versailles.inra.fr/Seq-Repository/Assemblies was successful for 68 of the 94 MQTL peaks reported in Table 2. Most of the gene models corresponded only to one peak. However, E3 ubiquitin ligases were found in 6 peaks, F-box domain in 5 peaks, glycosyltransferases and late embryogenesis abundant (LEA) hydroxyproline-rich glycoprotein in 4 peaks, root hair defective (RHD) 3 protein, peroxidases and pentatricopeptide repeat-containing protein in 3 peaks, and finally, ABC transporter proteins, NAC domains, embryogenesis transmembrane proteins, zinc finger domains and brevis radix proteins in 2 peaks.

## Discussion

Increasing wheat productivity is considered one of the major challenges to cope for wheat producers worldwide due to the need to ensure sufficient food supply for a growing world population in the current global climate change scenario.

Breeding for drought adaptation is particularly challenging because of the complexness of the target environments and the stress-adaptive mechanisms that plants use to diminish the negative effects of water deficit^2^. These mechanisms allow the plant to skip, evade or accept the negative effects of drought, and therefore have an important effect on final yield^25–27^.

Roots anchor the plant to the soil and take up water and nutrients, thus interacting with the water available in the soil and affecting biomass production^28^. The role of root architecture in the response to drought stress has been reported by several authors^3,29–32^. The wide morphological plasticity of root system to the different soil conditions, allow plants to adapt better, particularly under drought conditions. It has been demonstrated that wheat roots reduce their growth during periods of water scarcity while increasing the water uptake rates^33^ and extracting water stored in deep soil layers^34^. It has been suggested that a deep root system with an appropriate distribution of root density along the soil profile would confer some yield advantage on wheat grown in rainfed systems, where grain filling relies on deep soil water^35^. Therefore, identifying and introgressing favourable alleles controlling different root traits are desirable approaches for breeding programmes. The use of molecular markers accounting for a significant amount of the variability for relevant root traits, such as those analysed in the current study, may accelerate genetic gain by improving the efficiency of selection in segregating populations and conferring breeding programmes a great advantage in terms of selection for adaptation to drought.

In the last ten years, numerous studies identifying QTLs controlling root traits have been published, each of them using different traits, genetic backgrounds, mapping populations and/or environmental conditions. In order to reduce redundancies and to find consensus genomic regions harbouring the most robust and reliable QTLs among the mapping populations, in the current study QTL meta-analysis was performed.

The first step in the QTL meta-analysis is the projection of QTLs onto a reference or consensus map integrating a high number of molecular markers. However, not all of the QTLs reported for root traits were included in the analysis based on (1) the lack common markers between initial and consensus maps, and (2) some of the QTLs showed a low PVE, causing a large SI (>50 cM). Other studies reporting root QTLs were not considered (and not included in the reference list) because the map position of QTLs or the genetic maps were not reported in the papers. Although GBS and SNPs are being common used makers in the last years, most of the maps published before 2017 do not include other types of markers^36–39^, thus they are not useful for map comparison with previously developed maps. Or when SSRs and SNPs are present as in Iannucci *et al*.^19^ and Maccaferri *et al*.^22^ the studies are based on durum wheat where genome D is not represented. Additionally the use of the reference sequence of the wheat genome^40^ has the inconvenience that not all of SSR markers reported in this study are mapped and the different physical:genetic distance rate depending on chromosome regions, as previously reported^41–43^, could lead to a wrong determination of supporting intervals, increasing the complexity to identify common QTL regions. The majority of QTL studies reported in this work were based on mapping populations genotyped with SSR or DArT markers. Therefore, to include most of the QTLs, a consensus map integrating different types of markers (SSR, DArT, restriction fragment length polymorphism (RFLP), amplified fragment length polymorphism (AFLP), expressed sequence tag (EST), sequence-tagged sites (STS)) was constructed by merging three reference maps: (1) Wheat Composite 2004 (http://wheat.pw.usda.gov), (2) the consensus SSR map developed by Somers *et al*.^20^, and (3) the integrated map developed by Marone *et al*.^13^.

The usefulness of QTL meta-analysis is to integrate the QTL information previously published to define consensus genomic regions for a given trait and narrow down the confidence intervals of QTLs to tackle map-based cloning strategies more efficiently. However, QTL meta-analysis is highly dependent on the individual QTL mapping studies, their SI and projection quality^7^. For this reason, we projected only QTLs that had all the information required for QTL projection following an homothetic approach^24^ and meta-analysis using the BioMercator v4.2 software, such as LOD score, PVE, peak position, SI and flanking markers.

Using the Wheat_Consensus_2018 map, we were able to project 84% of the retrieved QTLs, most of them reporting a low PVE (96% lower than 0.2) and supporting the implication of many loci in the trait variation, each of them with a small effect.

QTL meta-analysis revealed the presence of 112 genomic regions harbouring root QTLs. Of these 94 represented consensus QTLs (MQTLs) and 18 remained as singletons (sQTL). Results of meta-analysis were supported by the QTL-overview density index^24^, which was computed to picture the regions involved in the genetic control of root-related traits. According to the QTL-overview index, 126 peaks exceeded the average value of the statistic and represented hotspots for root QTLs. Seventy-eight of the 94 MQTLs (83%) were located within overview peaks and 7 (7%) were located between two overview peaks. The remaining 10% did not correspond to overview peaks. Of the 18 sQTLs, 14 (78%) corresponded to overview peaks exceeding the average value and the remaining 4 (22%) were located within non-significant overview peaks. In addition, 37% of the MQTL corresponded to high-value overview peaks grouping 52% of the projected QTLs. The number of QTLs in the wheat genome was reduced by 82% or 85%, respectively, when all of the reported genome regions were considered, or only MQTLs. The average SI of MQTLs was reduced three-fold, increasing the mapping precision.

Previous studies reporting MQTLs for root traits have been carried out by Darzi-Ramandi^6^ and Iannucci^19^. From an initial database comprising 243 QTLs reported from 12 different experiments, Darzi-Ramandi^6^ used for meta-analysis only 53 QTLs located on chromosome groups 2 and 3, as they carried most of the QTLs for root traits. After meta-analysis, these authors grouped the QTLs into 8 MQTLs. Based on the position of flanking SSR markers, common regions for MQTLs were found between their results and those of the present study. On chromosome 2A, M-QTL1^6^ was located in a similar position to *Root_MQTL_18*. On chromosome 2B, M-QTL2 to M-QTL5 corresponded to the positions for *Root_MQTL_22*, *Root_MQTL_23*, *Root_sQTL_3* and *Root_MQTL_24*, respectively. On chromosome 3A, M-QTL6 was positioned between *Root_MQTL_32* and *Root_MQTL_33*, and M-QTL7 in a common region with *Root_MQTL_35*. Finally, on chromosome 3B, M-QTL8 corresponded to the position of *Root_MQTL_39*. Iannucci^19^ performed a QTL meta-analysis in durum wheat; from 12 previously published QTL studies comprising 100 QTLs, and 17 QTLs identified in their study, the authors found 34 MQTLs and 29 QTLs on 8 out of the 14 durum wheat chromosomes. Common MQTL regions with our study were found for the 8 chromosomes reported by these authors^19^ based on common flanking markers between Wheat_Consensus_2018 and the map reported by Maccaferri^22^ integrating SNP, SSR and DArT markers. Chromosomes 1B, 2A, 4B, 5B, 6A and 7B shared one MQTL among studies. Chromosome 3A harboured the highest number of common MQTL positions (5), and MQTL7-11 shared flanking markers with *Root_MQTL_32-36*. Finally, on chromosome 6B, 2 MQTLs were mapped in similar regions (MQTL30 – *Root_MQTL_77* and MQTL31 – *Root_MQTL_80*).

The annotation of the wheat genome sequence^40^ allowed us to identify gene models near the MQTL peaks with implications in a wide range of biological functions. Among them, those involved in root development or response to abiotic stress may be of special interest for breeding.

Six MQTL peaks corresponded to gene models with similarity to E3 ubiquitin protein ligases located on chromosomes 2D, 3A, 5A, 5D and 6B. These proteins play a role in the signal pathway of abiotic stresses tolerance, such as dehydration. Moreover, they are also involved in the regulation of plant development^44^. Additionally, five peaks corresponded to F-box domains that are the protein subunit of E3 ubiquitin ligases involved in the responses to abiotic stresses^45^. The overexpression of *TaFBA1*, a wheat F-box gene, improved the heat tolerance of transgenic tobacco^46^. These transgenic plants showed longer roots than wild type plants. Four MQTL peaks were located near gene models for LEA hydroxyproline-rich glycoproteins (chromosomes 3B, 4A and 5D). These proteins are involved in the response to abiotic stresses and they accumulated during the late embryo development. Although they are mainly expressed in seeds, they have also been found in roots during the whole cycle of development^47^.

Gene models for three MQTL peaks on chromosomes 2A, 5B and 6B corresponded to proteins containing pentatricopeptide repeats. These repeats have been found in the protein *TaMRRP* (TaMOR-related protein), which interacts with the wheat transcription factor gene *TaMOR* (MORE ROOT)^48^. The overexpression of this gene in *Arabidopsis* led to an increase in the number of lateral roots, and rice produced more crown roots, thus enhancing grain yield^48^. Based on the expression pattern, the authors suggested that *TaMOR* is involved in root initiation^48^. In our study, peroxidases were identified for three MQTL peaks (chromosomes 1B, 4B, 7A). The role of extracellular peroxidases in the production of superoxide in root cells as a part of the inducible defence response against abiotic and biotic stresses has been reported^49^.

Root hair defective 3 (*RHD3*) proteins were identified for three MQTL peaks on chromosomes 4B, 5A and 7B. Root hairs increase the surface area of the roots, providing major water and nutrient uptakes. In mature *Arabidopsis* plants, the *rhd3* mutation produces a reduction in plant ize^50^. In wheat, Shan *et al*.^51^ found that under salt stress the expression of *RHD3* was inhibited, stopping root growth.

Zinc finger proteins play important roles in several plant processes from growth regulation and development, signalling and responses to abiotic stresses. Two zinc finger domains were found for two MQTL peaks on chromosomes 1A and 2A. In wheat, the overexpression of the zinc finger protein *TaZFP34* in roots resulted in an increased root-to-shoot ratio, reducing shoot growth but maintaining root elongation^52^. The expression of *TaZFP34* in roots was upregulated during plant adaptation to drying soils caused by high salinity and dehydration.

On chromosomes 2D and 4A, ABC transporter proteins were located within the peaks for the *Root_MQTL30* and *48*. In *Arabidopsis*, a new ABC transporter controlling root development, *AtMRP5*, was identified^53^. A T-DNA insertion in this gene produced mutant plants with a decrease in root growth but an increase in the formation of lateral roots. The authors concluded that *AtMRP5* acts as an auxin conjugate transporter or that the mutants are defective for ion uptake, leading to changes in auxin concentration.

NAC domain-containing proteins are also present within MQTL positions in chromosomes 2D and 7B. Transcription factors of this kind have been described in many developmental processes and stress responses. In *Arabidopsis NAC1* is induced by auxins, promoting the development of lateral roots^54^, and the expression of *AtNAC2* is induced in response to salt stress, leading to an increase in the development of lateral roots^55^.

Brevis radix (*BRX*) acts as a key regulator of cell proliferation and elongation in the roots. In this work, two MQTLs were found on chromosomes 6A and 6D with gene models corresponding to this type of protein. In *Arabidopsis, brx-2*, an abscisic acid-hypersensitive mutant in root growth was isolated, providing evidence of the role of BRX-2 as a modulator of abscisic acid sensitivity in roots^56^.

The gene model TraesCS6B01G075800 was identified in the *Root_MQTL76* coding for a small auxin-upregulated RNA (SAUR)-like auxin-responsive protein. According to Guo *et al*.^57^, these genes are regulated by auxin and environmental factors. The authors identified in wheat the gene *TaSAUR75*, which was downregulated in roots under salt stress conditions. Its overexpression in *Arabidopsis* increased salt and drought tolerance and the plants showed increased root length.

Heavy metals are essential for plant growth, but high concentrations of them result in growth inhibition and toxicity. A heavy metal transport/detoxification gene model (TraesCS6A01G393200) was found for the *Root_MQTL73* peak. As the organ in contact with the soil metals, roots play an important role in adsorption and detoxification. These mechanisms are reviewed in Hall^58^.

The role of root primordium defective 1 (*RPD1*) has been described in *Arabidopsis*^59^. This gene is involved in maintaining active cell proliferation in the root primordium. When *rpd1* mutants were used, initially cell proliferation led to root primordia formation, although it stopped at an early stage^59^. The authors concluded that *rpd1* could not maintain the highly active cell division during the first stages of the root primordium. In our study, the gene model TraesCS1B01G440200 located within the peak position of *Root_MQTL12* on chromosome 1B corresponded to an RPD1 protein.

The gene model TraesCS7A01G154600 found in the peak of Root_QTL84 codes for a gibberellin receptor *GID1A*. Its interest lies in the fact that gibberellins are essential hormones regulating growth and development in plants.

Among other genes involved in root development although not found within MQTL peaks are expansin genes. The overexpression of *TaEXPB23*, a wheat expansin gene, improved drought tolerance stimulating the growth of the root system in tobacco without affecting other developmental processes^60^. VERNALIZATION1 (*Vrn1*), a key regulator of flowering time in cereals, was also involved in root architecture in wheat and barley^61^. QTLs for root traits were also detected in barley in the vicinity of *Vrn1*^62,63^.

The current study is the largest QTL meta-analysis for root traits carried out in wheat, although it only includes a fraction of all the published QTL information. Integrating the recently developed maps based on high-throughput genotyping with SNP arrays with the earlier maps will allow the regions involved in trait variation to be identified more precisely in order to tackle QTL cloning approaches successfully. Additionally, the integration of genome wide association studies will improve our understanding of the molecular bases of the quantitative traits involved in root architecture.

By integrating maps for meta-analysis, we were able to detect consensus QTL regions where robust SSR markers flanking MQTLs can be identified in the consensus map and used to transfer relevant QTLs by marker-assisted selection (MAS). The identification of several root traits within the same MQTL region will help breeders pyramiding different genes to model the architecture of new cultivars in response to the changing environment. From a breeding point of view, Löffler *et al*.^8^ defined the criteria that must be taken into consideration for MQTL selection to be used to accelerate breeding programs: (1) MQTLs should have small supporting intervals, (2) the MQTL should include a high number of initial QTLs and (3) the initial QTLs should be characterized by high PVE. Based on these criteria in this work we identified the most promising MQTL to be used for root improvement in wheat. Additionally, the use of common SSR markers in this study would allow to any laboratory or breeding company to implement MAS in their programs. In this respect, breeders have to take into consideration the quantitative inheritance of these traits, thus more than one MQTL must be selected for crop improvement.

The genome sequence of wheat (IWGSC 2018) published recently in open access will be an excellent tool for research and for the breeding community. As reported in this work, the identification of putative candidate genes for the traits of interest will accelerate the breeding process through finely directed research of specific gene models.

## Materials and Methods

### Consensus map construction

In order to include the maximum number of studies for QTL projection, a consensus map, Wheat_Consensus_2018, was constructed by integrating three wheat maps using the MergeMap software^64^ available at http://www.mergemap.org (Supplementary Material 1). MergeMap takes into account the individual maps to calculate the consensus marker order in each linkage group (LG). Initially, each LG is converted to directed acyclic graphs (DAGs)^65^ that are then merged into a consensus graph on the basis of their shared vertices. MergeMap tries to resolve conflicts by deleting a minimum set of marker occurrences. Finally, each consensus DAG was linearized using a mean distance approximation. Equal weight was given to all genetic maps (weight = 1.0).

The selected maps were (1) the Wheat_Composite_2004 map composed of 4403 markers, including SSR, RFLP and AFLP, available at http://wheat.pw.usda.gov (Composite_2004); (2) the consensus SSR map developed by Somers *et al*.^20^ composed of 1235 SSR markers (SSR_2004); and (3) the integrated map developed by Marone *et al*.^13^ composed of 3669 markers, including DArT, SSR, EST, STS and RFLP (Integrated_2013).

### The root QTL database

The literature was retrieved using the keywords ‘wheat root QTL’ from the Web of Science server (http://apps.webofknowledge.com). The QTL database was created from 30 studies published from 2006 to 2017, three of them carried out in durum wheat. A summary of the QTL studies is reported in Table 1. Although some of the papers reported QTLs for different traits, we took into account only those involving root traits. A total of 64 traits were considered and grouped into 15 main categories (Supplementary Material 2).

The QTL database reported information on type of cross, parents used, number of progenies, name of QTL, trait, LOD score, proportion of PVE by each QTL, QTL position on the original linkage map, SI and flanking markers.

### QTL projection

To compare the QTL positions detected in the studies, the original QTL data were projected onto the Wheat_Consensus_2018 map developed in the present work. Flanking markers for each QTL were located on the consensus map and projected on it following a homothetic approach proposed by Chardon *et al*.^24^. When SI was not reported in the original studies, the distance between flanking markers was taken as the SI. When the QTL peak position was projected, the SI on the consensus map was estimated for a confidence interval of 95% using the empirical formula proposed by Guo *et al*.^66^.

The frequency of identification of a root QTL for every cM position on the Wheat_Consensus_2018 map was estimated using the QTL-overview index^24^. To highlight regions with significant peaks, the average value of the statistic and a threshold for high values, calculated as five times the average value, were plotted. The statistic increases in a region with (1) the number of QTLs from different experiments, (2) the proximity between QTL positions and (3) the precision of the individual QTL position estimation.

### QTL meta-analysis

QTL meta-analysis was conducted using BioMercator v.4.2^67,68^, available at https://urgi.versailles.inra.fr/Tools/BioMercator-V4, for each chromosome. Two different approaches were used according to the number of QTLs per chromosome. The approach of Goffinet and Gerber^7^ was used when the number of QTLs per chromosome was 10 or lower. According to these authors, for n individual QTLs, BioMercator tests the most likely assumption between 1, 2, 3, 4 and n underlying QTLs. Decision rules are based on an Akaike information criterion (AIC), and the one with the lowest AIC value is considered the best fit. However, when the number of QTLs per chromosome was higher than 10, the approach of Veyrieras *et al*.^69^ was used. In this case meta-analysis determines the best QTL model based on model choice criteria from AIC, AICc, AIC3, a bayesian information criterion and average weight of evidence. The best QTL model was selected when values of the model selection criteria were the lowest in at least three of the five models. Consensus QTLs from the optimum model were regarded as meta-QTLs (MQTLs).

### Graphical representation

The QTL-overview index and MQTLs, together with a heatmap of the number of projected QTLs per 25 cM bin, were represented graphically using ClicO FS^70^.

### Gene annotation

Gene annotation corresponding to the MQTL peaks was performed using the gene models for high-confidence genes reported for the wheat genome sequence^40^, available at https://wheat-urgi.versailles.inra.fr/Seq-Repository/Assemblies. When the closest marker was not located in the sequence, flanking markers were used to project the peak position in the physical sequence.

## Supplementary information

Supplementary Info

## Data Availability

Data generated or analysed during this study are included in this published article (and its Supplementary Information files). Datasets are also available from the corresponding author on reasonable request.
