# Supplementary material for: Discovering consensus genomic regions in wheat for root-related traits by QTL meta-analysis
Source: Sci Rep. 2019 Jul 22;9:10537. doi: 10.1038/s41598-019-47038-2 (PMC6646344; doi:10.1038/s41598-019-47038-2)

Discovering consensus genomic regions in wheat for root-related traits by QTL meta-analysis

Jose Miguel Soriano and Fanny Alvaro

Sustainable Field Crops Programme, IRTA (Institute for Food and Agricultural Research and Technology), Lleida, Spain

Correspondence:
josemiguel.soriano@irta.cat

Supplementary Material

Supplementary material 1. Consensus map: Wheat_Consensus_2018
Supplementary material 2. Description and category of the traits collected for QTL meta-analysis.
Supplementary material 3. QTL overview index. The horizontal solid line represents the average value for the overview index and the horizontal dotted line represents the threshold for high values.

Supplementary material 1. Consensus map: Wheat_Consensus_2018

Marker	Chromosome	Position (cM)
Gli-A1	1A	0
wPt-5374	1A	2.3
wPt-3698	1A	3.5
wPt-4765	1A	6.3
XksuD14b	1A	7.5
wPt-2872	1A	12.4
wPt-7726	1A	12.4
wPt-9266	1A	12.4
wPt-9317	1A	12.4
wPt-1873	1A	13.3
wPt-2311	1A	14.6
XksuH9a	1A	15.5
wPt-5167	1A	16.2
wPt-8504	1A	16.2
wPt-7567	1A	16.8
Gli-A3	1A	18.5
Xcdo580	1A	19.1
wPt-2527	1A	23.1
wPt-7030	1A	23.6
wPt-8455	1A	23.7
wPt-4029	1A	27.2
XksuD14	1A	28.9
wPt-7872	1A	29.4
wPt-2384	1A	31.1
Xbcd265a	1A	32.9
wPt-4886	1A	35.2
XWD004db	1A	37.5
wPt-8770	1A	37.6
wPt-1167	1A	38.5
wPt-9752	1A	38.5
wPt-6290	1A	38.6
wPt-6122	1A	38.7
wPt-6358	1A	40.1
Glu-A3	1A	41.6
wPt-6709	1A	41.6
XWC2153	1A	43.5
Xgdm33	1A	44.8
Hg	1A	45.5
M77/P64.2	1A	46.5
Xpsr963.1	1A	46.5
M62/P64.5	1A	47.5
XSi2	1A	47.5
Xfba165	1A	47.5

Xgpw2186	1A	47.5
Xpsr11(Glu-3)	1A	47.5
wPt-1862	1A	47.8
Xwg180	1A	48.5
wPt-5707	1A	49.3
wPt-3983	1A	49.4
Pm3	1A	49.8
XGlu3	1A	49.8
Xmta16y	1A	49.8
Xpsp2999	1A	49.8
P38/M47-5	1A	50.8
Xgpw2246	1A	50.8
wPt-8072	1A	50.9
wPt-9423	1A	51
Xgpw2276	1A	51.2
tin1	1A	51.2
Xgwm136	1A	51.2
Xcfa2153	1A	52.2
XgpwE5	1A	52.2
XgpwE8	1A	52.2
XgpwE9	1A	52.2
Xmta14c	1A	52.2
Xcdo431	1A	53.2
Xfbb196	1A	54.2
Pm3a	1A	54.9
XksuD14.2	1A	55.2
Xbcd808a	1A	55.4
wPt-3870	1A	56
P32/M54-4	1A	56.1
M51/P65.5	1A	57.1
Pm3e	1A	57.6
Xfba254	1A	58
Xfba393	1A	58
Mlar	1A	59.1
Xcdo1160	1A	59.3
Xfba286	1A	59.9
Lr10	1A	61.3
Xfbb160	1A	61.8
Xfbb234	1A	61.8
Xmwg60	1A	61.8
Xfbb319	1A	62.8
Xgpw2032	1A	62.8
Xgwm33.1	1A	62.8
H9/H10/H11	1A	63.8
Xfba285	1A	63.8

Xhor12	1A	63.8
Xbarc263	1A	63.8
wPt-2150	1A	64
Xcfa2226	1A	64.2
Xcfd15	1A	64.9
Xgwm33	1A	66.8
XGli3	1A	67
Xabc156	1A	67
Xpsp3151	1A	67.1
Xwmc818	1A	67.9
P40/M60-138	1A	69.1
P42/M50-2	1A	69.1
Xwmc104	1A	69.1
Xwmc329.1	1A	69.1
Xcdo426	1A	69.1
wPt-9757	1A	69.3
P40/M61-2	1A	70.1
Xcfa2158	1A	70.1
Xwmc33	1A	70.8
P78/M71.5	1A	71.1
XChs3	1A	71.1
Xgpw2005	1A	71.1
XksuE19	1A	72.1
Xwmc336	1A	72.5
XksuE18	1A	73.2
Xgwm264	1A	74.5
wPt-9429	1A	76
Xbcd2015	1A	76.2
Xwmc95	1A	76.2
wPt-0164	1A	76.9
Xgdm136	1A	77.2
Xbcd98	1A	77.3
wPt-3904	1A	78.3
Xbarc83	1A	79.9
Xbcd98.1	1A	79.9
Xwmc24	1A	80.7
XksuG9a	1A	81.5
XcsIH69	1A	82.3
XgbxR507	1A	82.3
Xgwm357	1A	82.8
M77/P64.13	1A	83.3
Xmwg2021.2	1A	83.3
Xmwg67	1A	83.3
Xbarc119	1A	84.2
wPt-2436	1A	84.4

wPt-2251	1A	84.6
Xgwm666	1A	86.8
Xgwm164	1A	86.9
XTri	1A	87.4
Xfba298	1A	87.4
Xpsr381	1A	87.4
Xbarc148	1A	87.4
XBCD175	1A	88.4
XWB148	1A	88.4
Xfba26	1A	88.4
Xwmc286	1A	89.4
P34/M51-324	1A	90.4
Xmwg645	1A	90.4
Xabg373	1A	90.4
Xbcd1072	1A	90.4
Xgwm108	1A	90.4
Xbcd368	1A	90.4
Xpsr161	1A	90.4
P35/M54-7	1A	91.4
P40/M61-10	1A	91.4
Xabg452	1A	91.4
Xmwg758	1A	91.4
Xp219draI	1A	91.4
Xrz244	1A	91.4
Xcfd59	1A	91.9
Xbcd1072.1	1A	92.3
Xfbb107	1A	92.3
XEm	1A	93.3
Xbcd12	1A	93.3
Xbcd200	1A	93.3
Xcdo105	1A	93.3
Xwmc84	1A	93.3
P34/M50-6	1A	94.3
Xcdo98	1A	94.3
XksuG9	1A	94.3
Xpsp3001-161	1A	94.3
Xpsp3027	1A	94.3
Xwmc084	1A	94.3
M60/P64.8	1A	95.3
M83/P65.1	1A	95.3
Xbarc162	1A	95.3
Xwg605a	1A	95.3
Xbcd98.2	1A	96.3
Xcnl5	1A	96.3
Xwg605b	1A	96.3

Xwmc120	1A	96.3
P39/M69-239	1A	97.3
Xbarc120	1A	97.3
Xfba118	1A	97.3
Xfbb274	1A	97.3
Xwmc744	1A	97.4
Xcfd30	1A	97.6
Xbarc28	1A	98.1
Xcfd30a	1A	98.1
P34/M51-341	1A	99.4
Xgpw2142	1A	99.4
Xgpw2277	1A	99.4
Xgwm135	1A	99.4
Xwmc278	1A	100.4
Xwmc630	1A	100.8
Xbcd446	1A	100.8
Xgpw1134	1A	100.8
Xpsp3003	1A	100.8
Xwmc826	1A	101
Xwmc611	1A	101.1
Xwmc11	1A	101.2
Xcfa2135	1A	101.8
Xgpw2115	1A	101.8
Xwg983	1A	101.8
Xwmc469	1A	101.9
wPt-6074	1A	102.3
Xcdo312	1A	102.9
Xbarc240	1A	102.9
Xwmc183	1A	102.9
Xcfd22	1A	103.1
Xwmc550	1A	103.6
Xcdo473	1A	104
wPt-1011	1A	104.8
Xcdo92b	1A	105
Glu-A1	1A	105.4
XksuD49	1A	105.4
Xbcd22	1A	105.9
XgbxGx74	1A	105.9
Xgpw2045	1A	105.9
Xgpw2259	1A	105.9
Xpsr1327	1A	105.9
Xbcd1407	1A	106.9
Xcdo278Dra	1A	106.9
Xmwg632	1A	107.1
wPt-0527	1A	107.2

Xwmc304	1A	107.3
M17/P77.15	1A	107.9
Xgbx3076	1A	107.9
Xpsr2019.1(Aba8)	1A	108.9
Xwmc312	1A	108.9
Xwmc93	1A	109.2
P42/M51-295	1A	110.2
P40/M47-3	1A	111.2
Xabc155	1A	111.2
Xgpw2069	1A	113.2
Xcmwg733	1A	115.2
Xgpw2006	1A	115.2
P42/M47-2	1A	116.2
Xbcd738	1A	116.2
Xpsr325	1A	116.2
Xbcd187	1A	118.2
Xbcd265	1A	118.2
Xmsu488(Lec)	1A	120.2
Xmta17	1A	120.2
Xbcd304b	1A	121.2
XksuE3	1A	121.2
Xpsr634	1A	121.2
Xbcd1930	1A	123.2
Xcdo1188b	1A	123.2
Xpsr162	1A	123.2
Xbcd808b	1A	124.2
Xcmwg706	1A	124.2
Xbcd1889	1A	125.2
XksuH14	1A	126.2
Xfba316	1A	128.2
XksuE8	1A	128.2
Xcfa2129	1A	129.2
Xpsp3003b	1A	129.2
Xwmc9	1A	129.9
wPt-2406	1A	133.3
wPt-2976	1A	133.3
wPt-4709	1A	133.3
wPt-5274	1A	133.3
wPt-6754	1A	133.3
wPt-8644	1A	133.3
wPt-4658	1A	133.6
wPt-6005	1A	133.6
XAdh3	1A	133.7
Xmwg676	1A	133.7
wPt-5577	1A	134

wPt-0432	1A	134.3
Xfba92	1A	134.7
XksuH9	1A	136.7
Xgwm106	1A	137.7
Xbcd808	1A	138.2
XksuG34	1A	138.8
P35/M49-1	1A	140.8
Xcdo89	1A	140.8
Xgwm497	1A	140.8
wPt-8016	1A	140.8
Xbcd1434RV	1A	141.8
Xcfa2056	1A	145.8
Xcnl5a	1A	145.8
Xfba311	1A	145.8
Xmwg710	1A	145.8
Xfbb142	1A	147.8
Xfba234	1A	148.8
Xdupw38	1A	150.5
Xwmc673	1A	150.5
Xfbb171	1A	151.6
Xwmc51	1A	152.4
Xwmc716	1A	153.2
wPt-0258	1A	154.4
wPt-0128	1A	154.7
XksuH9b	1A	155
P78/M92-4	1A	155.6
Xmwg733	1A	155.6
wPt-5316	1A	156.3
Xfba279	1A	158.3
Xpsr967	1A	159.3
Xbcd304	1A	160.3
Xpsr386	1A	160.3
Xwmc254	1A	160.3
Xwmc59	1A	160.3
Xfba266	1A	161.3
Xfba268	1A	161.3
XgbxG097	1A	161.3
XgpwE7	1A	161.3
Xmwg912	1A	162.3
Xwmc237	1A	162.3
wPt-2847	1A	164.9
M21/P76.5	1A	167.1
Xbarc213	1A	168.1
XksuE11b	1A	168.1
M71/P77.5	1A	169.1

Xwg241	1A	169.1
Eps-1Am	1A	170.1
XksuE11.1a	1A	178.1
Xcdo393	1A	179.1
P78/M69.4	1A	180.1
wPt-1078	1A	183.4
wPt-2927	1A	183.4
Xbarc287	1A	183.6
Xbarc158	1A	185.6
Xbarc17	1A	186.3
Xbarc145	1A	187.1
Xcfa2219	1A	193.3
Xgwm99	1A	194.8
Gli-B1	1B	0
wPt-0308	1B	1.2
wPt-304810	1B	2.8
Xbcd1434	1B	4
Xopw13-435	1B	4
Xpsr11(Glu-3).1	1B	4
Yr10	1B	4
Glu-B3	1B	5
Xfbb250	1B	5
Xmta16a	1B	5
Xmtd161	1B	5
Xpsp3000	1B	5
XksuD14	1B	6
TC69046	1B	6.1
P31/M52-2	1B	6.9
Xmta14	1B	7.9
Ogliada	1B	8.7
Xpsr12(Glu-1)	1B	8.9
Xcfd15	1B	10.9
Xpsr11(Glu-3).2	1B	10.9
XHplc2-3	1B	11.9
Xwhs179a	1B	11.9
XMGL077	1B	12.9
Xgpw1143	1B	12.9
Xhor12	1B	12.9
rPt-8399	1B	13.2
Xpsr963	1B	13.9
wPt-3477	1B	14.4
XksuG9	1B	14.7
M71/P77.9	1B	15
Xmwg938	1B	15
XgpwE12	1B	16

Xmta16	1B	16
Xopah20-1100	1B	16
wPt-8627	1B	16.6
Xgwm835	1B	16.8
wPt-2988	1B	16.8
wPt-8949	1B	17.4
wPt-8267	1B	17.6
wPt-1912	1B	17.8
wPt-1328	1B	17.9
wPt-7094	1B	18.2
wPt-4107	1B	18.9
Xgwm24	1B	19.1
wPt-1560	1B	19.3
wPt-2052	1B	19.3
Xgwm608	1B	19.8
M65/P64.4	1B	21.2
P34/M51-285	1B	21.2
Xfbb234	1B	21.2
Xfbb260	1B	21.2
Xgwm550	1B	21.2
Xwmc818	1B	22.6
Xgwm374a	1B	22.7
wPt-0359	1B	23
wPt-3465	1B	23
TC87195	1B	23.2
Xwmc51	1B	23.5
wPt-4366	1B	23.6
wPt-6592	1B	23.7
tPt-5675	1B	23.8
Xmwg60	1B	23.8
Xwmc49	1B	24.3
Xwmc798	1B	24.7
wPt-5347	1B	24.7
wPt-3753	1B	25.3
wPt-4434	1B	25.4
wPt-5067	1B	25.4
Xfbb160x	1B	25.4
Xbarc128	1B	25.5
Xgwm666d	1B	25.6
Xgwm1100	1B	25.8
Xwmc51b	1B	26.2
wPt-4811	1B	26.5
P41/M48-174	1B	26.7
Xwmc619	1B	26.8
wPt-0974	1B	27.6

Xmwg837	1B	27.9
TC85294	1B	28
wPt-0655	1B	29.3
wPt-7833	1B	29.5
wPt-3177	1B	30
Xbarc60	1B	30.8
wPt-3282	1B	31.6
wPt-5164	1B	31.6
wPt-2230	1B	31.8
wPt-2999	1B	32
wPt-3824	1B	32.1
wPt-5312	1B	32.1
wPt-6427	1B	32.1
wPt-6434	1B	32.1
wPt-7529	1B	32.1
wPt-8986	1B	32.1
Xwmc406	1B	32.3
Xwmc500	1B	32.3
wPt-1781	1B	32.5
wPt-5065	1B	32.5
wPt-9903	1B	32.5
wPt-4605	1B	32.7
wPt-2614	1B	32.9
Xbarc8	1B	34.1
Xbarc119	1B	34.3
wPt-8930	1B	34.7
wPt-5485	1B	35
Xgwm413	1B	36.5
wPt-0222	1B	36.6
wPt-5678	1B	36.6
Xwmc329	1B	36.7
Xgdm36	1B	36.8
wPt-0260	1B	36.9
wPt-2706	1B	36.9
wPt-2744	1B	36.9
wPt-5385	1B	36.9
wPt-5899	1B	36.9
wPt-7242	1B	36.9
wPt-8087	1B	36.9
wPt-2762	1B	37.1
wPt-5363	1B	37.1
wPt-5562	1B	37.1
wPt-6777	1B	37.1
wPt-7038	1B	37.1
wPt-9977	1B	37.1

wPt-3889	1B	37.2
Xgwm133	1B	38.2
Xgwm33b	1B	38.2
Rg1	1B	38.8
Xcdo99	1B	38.8
Xwmc222	1B	38.8
XWB152o	1B	39.8
Xgwm106	1B	39.8
Xgwm33	1B	39.8
P34/M50-10	1B	40.8
Xfba285	1B	40.8
Xgwm264	1B	40.8
Xcfa2158	1B	41.8
XgbxR698	1B	41.8
Xgwm49	1B	41.8
Xfbb237	1B	42.8
Xgpw2162	1B	42.8
Xwmc85	1B	42.8
Xcdo388	1B	43.8
Xcfd92	1B	43.8
Xgwm337	1B	44.8
Xgwm603	1B	44.8
M85/P65.5	1B	45.8
Xgpw1239	1B	47.8
Xmwg68	1B	47.8
Xmwg77	1B	47.8
Xabc156	1B	48.8
Xwmc391	1B	48.8
Xcfd59	1B	48.8
P78/M87.8	1B	49.8
XGli3.2	1B	49.8
XksuF43	1B	49.8
Xwmc500.2	1B	49.9
Xksu136	1B	54
XksuE18	1B	55
XksuE19	1B	56
Xmwg758	1B	56
Xmwg645	1B	57
Xbcd340	1B	58
Xabc155	1B	59
P40/M61-5	1B	60
P78/M43.7	1B	60
Xbcd1124	1B	60
Xcdo1188	1B	60
Xcfd20	1B	60

Xcsu109	1B	61
Xpsr688	1B	61
Xbcd1796	1B	62
Xbcd2	1B	62
Nor-B1	1B	63
Xcdo1340	1B	63
Xcmwg733	1B	63
Xwmc52	1B	64
Xfbb35	1B	65
XgbxG080	1B	65
Xglk431	1B	65
Xwmc230	1B	65
Yr9	1B	65
Xglk136a	1B	66
Xrz166	1B	66
P35/M48-2	1B	67
Xabg373	1B	67
Xbcd200	1B	67
Xglk163	1B	67
P32/M52-120	1B	68
Xcdo1473	1B	68
XS13M61(590)	1B	68
XS25M51(280)	1B	68
Xmgb2	1B	68
Xpsr957	1B	68
Xutv111	1B	68
Xutv1356	1B	68
Xutv618	1B	68
Xutv780	1B	68
Xwmc326	1B	68
wPt-1399	1B	68
wPt-3103	1B	68
wPt-4510	1B	68
wPt-4726	1B	68
wPt-5800	1B	68
wPt-6012	1B	68
wPt-6325	1B	68
Xbarc187	1B	68
Xgwm18	1B	68
Xutv1109	1B	68.1
wPt-5801	1B	68.4
XS13M61(370)	1B	69
wPt-8682	1B	69.4
wPt-1116	1B	69.6
wPt-6117	1B	70

wPt-6833	1B	70
wPt-8616	1B	70
Xwmc128	1B	70.2
Xgdm28	1B	70.3
Xgwm494	1B	70.3
Xwmc31	1B	70.3
Xgdm33	1B	70.3
Xgdm33b	1B	70.3
Xpsr596a	1B	70.7
Xutv1518	1B	71.1
Xbcd1449	1B	71.9
Xbcd442a	1B	71.9
Xbcd762	1B	71.9
Xcmwg645	1B	71.9
Xfba177	1B	71.9
Xmwg695	1B	71.9
Xwg605	1B	71.9
Xgwm498	1B	71.9
Nor1	1B	72
Xcdo127	1B	72.2
Xgwm947	1B	73
Xbcd338	1B	73.2
Xwmc156.2	1B	73.2
Xcfd59c	1B	73.2
Xwmc419	1B	73.2
Xcdo1173	1B	73.7
Xcdo618	1B	73.7
Xcdo92	1B	73.7
Xfbb283	1B	73.7
Xwg811	1B	73.7
Xdupw532	1B	73.7
Xpsr949	1B	73.7
wPt-306450	1B	74.1
Xwmc611	1B	74.5
Xcdo98.2	1B	74.6
XgbxG178	1B	74.6
XgbxR059	1B	74.6
Xcfd2	1B	75.6
Xmex3M8f	1B	75.6
Xwmc320	1B	75.6
Xwmc269	1B	75.9
Xwmc597	1B	76.3
Xgwm273	1B	76.5
QLr.sfr-1BS	1B	77.5
Xcfa2241	1B	77.5

Xfba28	1B	77.5
XgbxG263	1B	77.5
Xmwg837a	1B	77.5
Yr15	1B	77.5
Xutv1181	1B	78
Xcfd65	1B	78.5
Xgdm136	1B	78.5
Xgwm762	1B	78.6
Xbarc120	1B	78.8
Xbcd12	1B	78.9
Xcdo278	1B	78.9
XWC0065	1B	79.9
Xcmwg758	1B	79.9
Xfba171	1B	79.9
Xfbb121	1B	79.9
Xgwm11	1B	79.9
wPt-1374	1B	80.2
Xbcd98b	1B	80.6
Xbarc137	1B	80.6
Xfba319	1B	80.6
Xfbb251	1B	80.6
Xwg822	1B	80.6
Xwmc626	1B	81.1
CA679329a	1B	81.4
Xwmc216	1B	81.4
Xwmc213	1B	82.1
Xwmc813	1B	82.2
wPt-3084	1B	82.7
Xwmc694	1B	82.8
Xwmc156	1B	83.1
Xgwm582	1B	83.4
Xwmc265	1B	83.5
Xgwm374	1B	84
Xbarc240	1B	84.2
Xbcd386	1B	84.2
Xgbx3147	1B	84.2
BJ318987	1B	84.3
Xfbb180	1B	84.6
Xfbb67	1B	84.6
Xgwm403a	1B	84.6
XksuF43a	1B	84.6
Yr24	1B	84.6
wPt-5296	1B	84.7
P43/M62-3	1B	84.9
Xbcd1889	1B	84.9

Xwmc277	1B	84.9
Xbarc181	1B	84.9
Xcfa2129	1B	85.9
Xgwm131	1B	86.1
wPt-2389	1B	86.2
Xcfd48	1B	86.5
Xbarc83	1B	86.7
Xbarc174	1B	86.9
Xpsr2019.2(Aba8)	1B	86.9
Xutv786	1B	87.2
P37/M64-249	1B	87.9
Rf3	1B	87.9
XPc-B4e	1B	87.9
Xbarc302	1B	87.9
Xfba245	1B	87.9
Xmta17	1B	87.9
Xmwg68a	1B	87.9
Xgdm101	1B	88
P44/M54-1	1B	88.9
Xfbb329	1B	88.9
Xfbb90	1B	88.9
Xglk136	1B	88.9
Xpsr325	1B	88.9
Glu-B1	1B	89.9
Xcdo637	1B	89.9
Xfbb255	1B	89.9
Xmwg938b	1B	89.9
wPt-8168	1B	89.9
Xmag620	1B	90.7
Xmgb40	1B	90.7
Xwmc416	1B	90.7
wPt-3451	1B	90.8
wPt-6240	1B	90.8
wPt-9792	1B	91.2
Xutv1334	1B	91.8
Xpsr648	1B	92
wPt-0705	1B	92
wPt-2315	1B	92
wPt-303890	1B	92.2
Xwmc134	1B	92.5
Swes578	1B	93.6
wPt-3579	1B	93.8
Xcdo388a	1B	93.9
Xgdm126.1	1B	93.9
Xpsr967	1B	93.9

Xpsr162	1B	93.9
wPt-0202	1B	94.1
wPt-3227	1B	94.1
wPt-0506	1B	94.5
wPt-0463	1B	94.7
wPt-3566	1B	95.3
Xdupw214	1B	96.4
Xgwm403	1B	96.9
P31/M53-3	1B	98.4
Xfba34a	1B	98.4
XksuE11a	1B	98.4
CA651264	1B	99.4
M17/P65.1	1B	99.4
Xbcd1150a	1B	99.4
XksuA1	1B	99.4
Xwmc85a	1B	99.4
P39/M50-10	1B	100.4
Xbcd1150b	1B	100.4
Xbcd1150	1B	101.4
Xwmc206	1B	103.4
Xbcd265d	1B	104
Xgwm274	1B	104.1
wPt-5011	1B	104.1
Xgwm153	1B	104.8
Xbarc81	1B	105.1
P32/M54-5	1B	105.2
P35/M51-2	1B	105.2
P38/M47-9	1B	105.2
P41/M51-182	1B	105.2
Xbcd265	1B	105.2
Xcdo1160	1B	105.2
Xcdo393	1B	105.2
Xmwg733	1B	105.2
Xbcd304	1B	105.2
Xbcd310	1B	105.2
wPt-1247	1B	107.7
wPt-1818	1B	107.7
wPt-9409	1B	107.7
Xwmc631	1B	107.8
Xwmc766	1B	107.8
Xwmc673	1B	107.9
Xbarc188	1B	108.1
XksuG34	1B	108.2
wPt-1403	1B	108.3
wPt-9032	1B	108.5

wPt-6975	1B	108.6
CA679329b	1B	108.8
P78/M72.4	1B	108.9
rPt-7906	1B	109.2
Xbcd441	1B	109.9
XgbxG160	1B	109.9
Xgwm806	1B	110.2
Xgwm268	1B	111.2
XWB188h	1B	111.8
Xbarc61	1B	111.8
Xwhs179	1B	111.8
Xgwm124	1B	111.8
P78/M92.2	1B	112.8
Xcmwg733a	1B	112.8
Xmwg676	1B	112.8
Xcdo1196	1B	113.8
Xmwg69	1B	115.8
M67/P77.5	1B	116.8
Xbcd442	1B	116.8
Xgpw2071	1B	116.8
M51/P65.3	1B	117.8
Xutv1425	1B	118.3
BE419757	1B	118.4
Xfba178	1B	119.7
Xutv704	1B	119.7
wPt-0459	1B	120.1
Xgwm544	1B	121.3
Xbcd21	1B	121.4
Xglk259	1B	121.4
Xmwg984	1B	121.4
Xfbb275	1B	122.4
Xfba309	1B	123.4
wPt-2257	1B	124.1
Xfbb260a	1B	124.3
wPt-7066	1B	124.7
wPt-9809	1B	124.9
Xbcd508	1B	127.1
Xfba294	1B	127.1
wPt-0097	1B	127.2
Xfba134	1B	128.9
Xpsp3100	1B	128.9
Xbcd1261	1B	130.9
Xcdo1189	1B	130.9
Xbcd1514	1B	131.9
Xcfa2147	1B	132.9

wPt-3475	1B	132.9
wPt-0944	1B	133
wPt-2526	1B	133.5
wPt-4129	1B	133.9
wPt-4532	1B	134
F79	1B	134.6
wPt-5034	1B	134.8
wPt-5061	1B	134.9
rPt-2940	1B	135.2
wPt-5281	1B	135.5
XS13M61(430)	1B	135.9
wPt-5316b	1B	136.5
TC81688	1B	139.2
wPt-5907	1B	140.8
wPt-2205	1B	141.4
wPt-4688	1B	143.1
Xbcd1562	1B	144.4
F109	1B	144.6
wPt-8832	1B	149.7
rPt-5412	1B	150.3
Xwmc830	1B	150.6
Xwmc719	1B	150.7
Xwmc44	1B	153.2
Xhbe248	1B	170.4
TC88378	1B	174.6
Xcfa2292	1B	175.7
Xcdo346	1B	176.7
Xfba268	1B	178.7
Xfba34	1B	178.7
Xwmc367	1B	180.2
Xgwm259	1B	184.4
Xgwm328	1B	184.7
Xgpw1077	1B	185.7
Xgwm793	1B	185.7
wPt-3571	1B	186.3
Xfba266	1B	186.7
wPt-4721	1B	186.8
wPt-6142	1B	186.8
Xwmc728	1B	188.4
XksuE11	1B	188.4
Xmwg912	1B	189.4
XksuI27	1B	190.4
P36/M67-1	1B	191.4
Xwmc113	1B	191.4
Xgwm140	1B	191.4

Xbarc80	1B	193.9
Lr46	1B	194.9
Yr29	1B	194.9
wPt-9028	1B	195.2
wPt-5253	1B	195.3
wPt-8866	1B	195.4
wPt-2968	1B	195.9
wPt-5915	1B	196.3
wPt-1973	1B	196.4
wPt-5721	1B	196.6
wPt-3950	1B	196.8
tPt-6091	1B	197.3
wPt-1672	1B	197.3
wPt-1786	1B	197.4
wPt-4361	1B	197.5
Xgwm659	1B	198.2
wPt-2485	1B	198.7
CA677684a	1B	199.8
Xcwem32	1B	202.6
wPt-4651	1B	202.7
wPt-5577b	1B	203.2
wPt-1313	1B	203.3
wPt-1770	1B	203.3
wPt-7746	1B	203.3
wPt-8245	1B	203.3
Swes947	1B	204.6
Xpsr593	1B	228.5
CD9b	1B	274.9
D1.1_ctg10323	1D	0
D1.1_ctg10469	1D	0
D1.1_ctg18	1D	0
D1.1_ctg6064	1D	0
D1.1_ctg8441	1D	0
D1.1_ctg8730	1D	0
D1.1_ctg9093	1D	0
Lr21	1D	0
Lr60	1D	0
Xbcd1431c	1D	0
Xfbb322	1D	0
Xgwm155	1D	0
Xmtd161b	1D	0
Xbcd1661	1D	1
XgbxG035d	1D	1
Xwg184	1D	1
Xcfa2170	1D	2

Xfba167	1D	2
Xfbb293	1D	2
Xgpw2255	1D	3
Xwmc153	1D	3
Xwmc173	1D	3
Xfbb250a	1D	4
Xmwg570	1D	4
Xubp19.2	1D	4
D1.1_ctg3776_1	1D	5
P32/M49-1	1D	5
P34/M51-304	1D	5
P39/M49-178	1D	5
XksuE19	1D	5
D1.1_ctg11242	1D	6
D1.1_ctg19	1D	6
D1.1_ctg794	1D	6
D1.1_ctg9381	1D	6
Xcdo388b	1D	6
XgbxR698b	1D	6
Xmwg938b	1D	6
Xpsr11(Glu-3)	1D	6
Rg2	1D	7
Xfba199	1D	7
Xmwg68	1D	7
Xmwg837b	1D	7
Xabc156	1D	7
XksuD14a	1D	7
Xgwm147	1D	9.8
D1.1_ctg11208	1D	11
D1.1_ctg11431	1D	11
D1.1_ctg12342	1D	11
D1.1_ctg3156	1D	11
D1.1_ctg4584	1D	11
D1.1_ctg4680	1D	11
D1.1_ctg6495	1D	11
D1.1_ctg8678	1D	11
Xfbb234b	1D	11
Xfbb237a	1D	11
Xfbb67c	1D	11
XgbxG746	1D	11
Xgpw2067a	1D	11
Xhor12b	1D	11
XksuF43a	1D	11
Xmta16b	1D	11
Xbcd1434	1D	11

wPt-9181	1D	11
Glu-D3	1D	12.3
Gli-D1	1D	13.1
H22	1D	13.8
Xmwg60b	1D	13.8
Xwhs179.1	1D	13.8
Xwmc85	1D	13.8
Xfba329	1D	14.8
Xfbb196	1D	14.8
Xfbb260b	1D	14.8
Xfbb319b	1D	14.8
Xgpw1170	1D	14.8
Xgpw363	1D	14.8
Xgwm264	1D	14.8
Xgdm33	1D	16.7
Xgdm33c	1D	17.4
Xbarc149	1D	25.7
Xwmc147	1D	28.7
Xgwm33	1D	34.3
Xpsr1327	1D	34.8
Xwmc432	1D	34.8
P34/M511	1D	35.8
Xgwm33c	1D	35.9
P39/M49-8	1D	36.4
Xfba40b	1D	36.4
Xcfd15	1D	36.4
Xwmc336	1D	36.4
Xgwm33.3	1D	37.4
Xcfd61	1D	39.7
Xcnl7.1	1D	40.4
Xcfd58	1D	41.1
Xmwg837	1D	41.4
Xcfd21	1D	41.6
Sr33	1D	45.4
Xmta16x	1D	45.4
Xwhs179.2	1D	45.4
Xbarc152	1D	45.4
wPt-3707	1D	45.5
wPt-3790	1D	45.5
Ogliadc	1D	45.6
XgpwE8b	1D	45.6
Xpsp3000	1D	45.6
Xwmc222	1D	45.6
wPt-3738	1D	46.7
Xabc156b	1D	47.3

Xcfd92	1D	47.6
wPt-4196	1D	48.2
P32/M59-2	1D	50.8
P34/M47-3	1D	50.8
Xgwm106	1D	50.8
D1.1_ctg3776_2	1D	51.8
XSi_2	1D	51.8
XksuE19b	1D	51.8
XgAmmA	1D	52.8
XksuE18a	1D	54.8
P32/M55-3	1D	58.8
Xmta14a	1D	58.8
XksuD14B	1D	60.8
Xhor12d	1D	61.8
Xubp19.1	1D	66.8
D1.1_ctg5162	1D	69.8
D1.1_ctg5357	1D	69.8
XksuG9	1D	69.8
Xgwm848	1D	70.9
Xbarc119	1D	73.3
Xcdo89	1D	73.6
wPt-0413	1D	73.9
wPt-9380	1D	73.9
wPt-7038b	1D	74.2
Xgwm191	1D	74.4
Xbcd200	1D	74.6
wPt-5503	1D	75.3
P37/M64-288	1D	75.6
Xgwm337	1D	76.2
Xbarc229.1	1D	76.6
Xcdo580	1D	77.6
Xwmc489	1D	77.9
Pm24	1D	78.2
Xcfd59b	1D	78.2
Xcdo1173	1D	78.6
Xgwm603	1D	79
Xcfd59	1D	79.6
D1.1_ctg10420	1D	79.7
D1.1_ctg620	1D	79.7
D1.1_ctg7447	1D	79.7
D1.1_ctg8782	1D	79.7
P34/M50-8	1D	79.7
P36/M68-2	1D	79.7
P39/M39-5	1D	79.7
P40/M51-8	1D	79.7

P41/M61-7	1D	79.7
Xbcd22	1D	79.7
Xbarc99	1D	79.7
Xcfd72	1D	79.8
Xcfd65	1D	81.1
Xwmc429	1D	81.5
P35/M39-5	1D	82.5
Xbarc148	1D	85.1
Xgwm608	1D	85.3
Xbarc229	1D	85.5
XcsEND2-4	1D	85.6
Xbarc162	1D	85.6
Xgwm458	1D	86.3
Xbarc240	1D	86.9
Xwmc339	1D	87.3
Xwmc590	1D	88
D1.1_ctg5940	1D	89.6
D1.1_ctg839	1D	89.6
Xfbb377d	1D	89.6
Xbarc169	1D	90.6
Xwmc216	1D	91.4
wPt-7711	1D	91.4
wPt-8854	1D	91.4
wPt-0077	1D	91.6
Xcfd19	1D	91.8
Xbcd402	1D	93.1
wPt-3945	1D	94.1
P31/M52-3	1D	94.1
wPt-9857	1D	94.3
wPt-4988	1D	95
P36/M54-8	1D	95.1
D1.1_ctg3072	1D	96.1
D1.1_ctg4533	1D	96.1
D1.1_ctg4968	1D	96.1
D1.1_ctg8047	1D	96.1
D1.1_ctg9710	1D	96.1
Xfba383	1D	96.1
Xfba8	1D	96.1
Xpsr634	1D	96.1
Xfba118a	1D	97.1
wPt-3743	1D	97.6
Xglk558	1D	98.1
Glu-D1	1D	98.7
D1.1_ctg1364	1D	99.1
D1.1_ctg6012	1D	99.1

D1.1_ctg7989	1D	99.1
Xbcd1150Dra	1D	99.1
Xfba250b	1D	99.1
Xwmc36	1D	100.1
Xcfa2129	1D	100.3
Xcfd48	1D	100.4
D1.1_ctg11375	1D	101.1
Xmwg967	1D	101.1
Xbarc62	1D	101.1
Xgdm126	1D	102.1
Xcfd19a	1D	103
Xwmc93	1D	104
Xfbb194	1D	104.5
Xwmc261	1D	104.5
Xwmc673	1D	104.5
Xwmc732	1D	104.7
Xpsr544	1D	105.3
Xcfd48b	1D	105.7
Xcdo312	1D	106.3
Xgpw357	1D	107.3
Xgpw2118	1D	109.3
XksuD49	1D	109.3
wPt-6059	1D	109.8
Xgwm55	1D	110
D1.1_ctg7441	1D	110.3
Xgpw315	1D	110.3
XksuG55	1D	110.3
Xmta17c	1D	110.3
Xcfd63	1D	110.9
D1.1_ctg4643	1D	111.8
D1.1_ctg6587	1D	111.8
Xfbb90b	1D	111.8
XksuG2	1D	111.8
Xgwm642	1D	111.8
Xcfd28	1D	113.8
Xcfd83	1D	113.8
Xfba268	1D	113.8
Xfbb12	1D	113.8
P36/M47-5	1D	114.8
Xgpw360	1D	114.8
D1.1_ctg1648	1D	115.8
D1.1_ctg2680	1D	115.8
Xbcd1930	1D	115.8
Xmwg733c	1D	115.8
D1.1_ctg1075	1D	116.8

D1.1_ctg5094	1D	116.8
Xcdo105	1D	116.8
Xgwm820	1D	116.8
Xwmc813	1D	117.9
Xcmwg733.3	1D	117.9
Xcmwg695	1D	118.9
Xmwg676d	1D	119.9
Xcsd19(Adh)	1D	120.9
Xmwg701	1D	120.9
Xbarc66	1D	122.9
Xfbb275c	1D	122.9
Xpsr95_Em	1D	122.9
XATPase	1D	123.9
Xcmwg701	1D	125.9
wPt-10060	1D	126.5
wPt-1070	1D	126.5
wPt-1597	1D	126.5
wPt-2541	1D	126.5
wPt-2839	1D	126.5
wPt-3636	1D	126.5
wPt-4427	1D	126.5
wPt-6503	1D	126.5
wPt-7057	1D	126.5
wPt-7421	1D	126.5
wPt-1531	1D	126.6
wPt-1685	1D	126.6
wPt-2897	1D	126.6
wPt-1263	1D	126.7
wPt-4687	1D	126.8
wPt-6560	1D	126.8
wPt-7092	1D	126.9
Xbcd1261A	1D	127.9
Xmwg693	1D	127.9
Xpsp3037	1D	127.9
wPt-7437	1D	127.9
Xbarc271	1D	128.8
Xfba279a	1D	128.8
wPt-7697	1D	131.1
D1.1_ctg1298	1D	131.7
Xbcd508c	1D	131.7
Xbarc346	1D	137.7
P36/M35-3	1D	138.7
Xgwm232	1D	138.7
Xcfa2187c	1D	139.7
Xcmwg693	1D	139.7

P41/M35-1	1D	140.7
P32/M53-2	1D	141.7
P38/M54-6	1D	141.7
P41/M40-4	1D	141.7
P33/M49-3	1D	142.7
P34/M51-205	1D	142.7
P36/M49-3	1D	142.7
P36/M50-5	1D	142.7
P36/M54-7	1D	142.7
P37/M51-6	1D	142.7
P38/M50-2	1D	142.7
Xcdo393A	1D	142.7
P32/M52-145	1D	143.7
P34/M51-8	1D	143.7
P35/M76-2	1D	143.7
P42/M50-6	1D	143.7
Xcfd282	1D	143.7
Xcdo1420	1D	144.7
Xgpw1077	1D	145.7
Xgpw2196	1D	145.7
XksuE11.1D	1D	146.7
D1.1_ctg5083	1D	147.7
Xgpw2224	1D	147.7
XksuI27.1	1D	147.7
Xgdm126.2	1D	149.7
Xwg241b	1D	150.7
XgbxG097b	1D	151.7
Xcfa2147b	1D	157
Xcfd32	1D	157.7
Xcfa2147	1D	158
Xwmc609	1D	159
Xwmc405	1D	160.9
Xgdm111	1D	161.8
Xgwm241	1D	165.2
wPt-8545	1D	165.3
wPt-4180	1D	177.5
Xcfd27	1D	188.8
wPt-3157	1D	194.4
wPt-0003	2A	0
wPt-7901	2A	1.4
wPt-6687	2A	1.8
wPt-9793	2A	2.3
Xfba88	2A	30.4
Xwmc382	2A	31.4
Xcmwg682a	2A	32.4

XWB124pbb	2A	33.4
Xcdo456a	2A	33.4
Xfba70a	2A	33.4
Xfba82a	2A	33.4
Xwmc407	2A	33.4
Xcdo447a	2A	34.4
Xgwm210	2A	34.4
Yr17/Lr37/Sr38	2A	34.4
Xbarc1138a	2A	35.4
Xgpw1162	2A	35.4
Xgwm496	2A	35.4
Xgpw2125	2A	36.4
Xbcd718	2A	37.4
P78/M87.3	2A	38.4
XgbxG747b	2A	38.4
Xgwm614	2A	40.4
Lr17a	2A	41.1
wPt-0071	2A	41.1
Xwmc667	2A	43
Xpsr131	2A	43.2
Xgwm497	2A	43.9
Xfbb61	2A	44.9
Xwmc154	2A	45
wPt-6148	2A	45
wPt-8328	2A	45
Xbarc124	2A	45.9
Xcfd36	2A	45.9
Xfba198	2A	45.9
Xfba329	2A	45.9
Xfbb289	2A	45.9
Xbarc1138	2A	46.9
Xbarc212	2A	46.9
wPt-2147	2A	48.2
Xbcd348a	2A	48.4
Xcfa2201	2A	48.4
Xgbx3818y	2A	48.4
Xgpw2018	2A	48.4
Xwmc326	2A	49.3
wPt-8925	2A	50
wPt-9277	2A	50.1
wPt-7175	2A	50.3
tPt-2481	2A	50.4
tPt-4602	2A	50.5
Xcdo1090	2A	50.7
Xfba280	2A	50.7

XksuH9	2A	50.7
Xgwm636	2A	50.7
Xbcd1970a	2A	51.7
Xfbb72	2A	51.7
XksuD18	2A	51.7
Xgwm296	2A	51.7
Xcdo57	2A	52.7
Xfba3	2A	53.7
Xpsp3153	2A	54.7
Xgpw2127	2A	55.7
Xgwm359	2A	55.7
Xbarc231	2A	56.7
Xfba178	2A	56.7
P78/M92.10	2A	57.7
Xgpw2089	2A	57.7
Xbcd152a	2A	59.7
Xfbb226a	2A	59.7
Xbcd1184a	2A	60.7
Xpsr109(RbcS)	2A	60.7
Xpsr133	2A	60.7
Xpsr332	2A	60.7
Xwsu1(Dor6).1	2A	60.7
Yr32	2A	60.7
XgbxG036a	2A	61.7
Xcdo1281a	2A	62.7
XPAACTelo1	2A	63.7
Xbcd161a	2A	63.7
Xbcd1688a	2A	63.7
Xbcd855a	2A	63.7
Xcdo405a	2A	63.7
Xfba106a	2A	63.7
Xfba374a	2A	63.7
Xfbb329	2A	63.7
XgbxG281	2A	63.7
Xabc170	2A	65.7
Xfba272	2A	65.7
Xwmc69	2A	65.7
P78/M43.9	2A	67.7
Xbcd1184	2A	67.7
Xfba300	2A	67.7
TC90640	2A	69.7
TC90641	2A	69.7
Xbarc128	2A	69.7
Xdupw210	2A	69.7
Xmag3807	2A	69.7

Xcdo665	2A	69.7
tPt-1041	2A	69.8
wPt-4197	2A	69.8
wPt-6245	2A	69.9
wPt-8049	2A	69.9
wPt-4984	2A	70
wPt-9672	2A	70.1
Xgwm512	2A	70.3
Xmag3996	2A	70.3
wPt-1601a	2A	70.3
wPt-4533	2A	70.3
Xcdo1376a	2A	72.9
Xbcd152	2A	74.9
Xrz395a	2A	74.9
Xwmc728	2A	75.4
Xwmc177	2A	75.9
wPt-0102	2A	76
wPt-0277	2A	76
wPt-3611	2A	76
wPt-3744	2A	76
Xwmc598	2A	76.4
Xwmc630	2A	76.5
Xgwm71	2A	76.9
tPt-5584	2A	76.9
Xwmc602	2A	77.3
wPt-1368	2A	77.4
Xwmc149	2A	77.8
Xgwm328a	2A	78.8
wPt-5839	2A	79
wPt-0274	2A	79.1
wPt-3896	2A	79.5
Xgwm372a	2A	79.7
XksuG5a	2A	80.7
Xopag08-300	2A	80.7
Xwmc826	2A	81.2
wPt-7026	2A	81.4
CA658758	2A	82
Xfbb359a	2A	82.4
wPt-7285	2A	82.4
wPt-7306	2A	82.7
Xfbb226	2A	83.3
Xgbx3832	2A	83.3
wPt-4201	2A	83.5
M83/P65.2	2A	84.2
Xwsu1(Dor6).2	2A	84.2

Xwmc827	2A	85.4
XgbxG542	2A	85.9
Xgpw2111	2A	86.9
Xopal19-700	2A	86.9
Xgpw2204	2A	87.9
Xgwm294a	2A	87.9
Xwmc453	2A	87.9
wPt-9320	2A	88.4
Xbcd1688	2A	89
Xcdo405	2A	89
Xgpw2225	2A	89
Xgpw2253	2A	89
wPt-3037	2A	89.4
wPt-2435	2A	89.7
Xfba106	2A	90
Xwmc522	2A	90
Xmag2090	2A	90.2
Xwmc32	2A	90.5
wPt-2120a	2A	90.8
TC82001	2A	91.2
Xwmc474	2A	91.5
Xwmc296	2A	91.9
Xwmc792	2A	92.4
Xgwm339	2A	94
Xgwm515	2A	94.4
Xfba374	2A	94.6
Xfbb359	2A	94.6
Xgwm10	2A	94.6
Xgwm425	2A	94.6
Xgwm448	2A	94.7
Xgwm275	2A	94.8
Xgwm95	2A	95.2
Xcfd2	2A	96.2
Xbcd161	2A	97.2
Xcdo1376	2A	97.2
Xgwm122	2A	97.2
M85/P65.4	2A	98.2
Xcdo1281	2A	98.2
Xgpw1148	2A	98.2
Xpsr107	2A	98.2
Xpsr135	2A	98.2
Xgwm10a	2A	98.2
Xbcd855	2A	98.6
Xgwm249	2A	98.7
Xbarc309	2A	99.7

Xcdo447	2A	99.7
Xfba70	2A	99.7
Xfba82	2A	99.7
Xfbb212	2A	99.7
Xrz395b	2A	99.7
Xbcd1709	2A	100.2
Xgwm71b	2A	100.3
Xcdo456	2A	100.7
Xfba71	2A	100.7
Xfbb353a	2A	100.7
Xgbx4986	2A	100.7
Xcfa2164	2A	100.7
Xgpw2272	2A	101.7
Xgsy333	2A	101.7
Xpsp3088	2A	101.7
Xwmc246	2A	101.7
Xgwm558	2A	101.7
XksuF37a	2A	101.9
Xmgb225	2A	101.9
Xmgb5	2A	101.9
Xpsr388	2A	101.9
Xwmc63	2A	101.9
Xutv1340	2A	102
XS25M51(350)	2A	102.1
wPt-6461	2A	102.1
Xwmc51	2A	102.2
Xwmc702	2A	102.2
wPt-8621	2A	102.3
wPt-1203	2A	102.5
wPt-1217	2A	102.5
wPt-8243	2A	102.5
wPt-8826	2A	102.5
Xwmc644	2A	102.6
Xgdm101	2A	103
Xgwm1045	2A	103
Xgwm895	2A	103
Xmag1010	2A	103
Xwmc744	2A	103
wPt-3114	2A	103
Xgwm630	2A	103.1
Xgwm473	2A	103.4
Xwmc794	2A	103.9
Xwmc632	2A	104.6
Xgwm66	2A	104.9
M62/P64.3	2A	105

P78/M63.2	2A	105
XWC2263b	2A	105
Xbcd348	2A	105
Xpsr575.1	2A	105
Xwmc179	2A	105
Xgwm304	2A	105
Xfbb278	2A	106
Xgwm328	2A	106
Xwmc455	2A	107
wPt-7049	2A	107.1
Xgwm372	2A	107.5
Xglk554	2A	108.5
TC81096	2A	111.7
Xbarc15	2A	111.9
wPt-0921	2A	111.9
Xbcd543	2A	112.1
Xcfa2099	2A	112.1
Xcfa2043	2A	114.1
Xcmwg682	2A	114.1
Xwsu1	2A	114.1
P78/M69.9	2A	115.1
P32/M48-213	2A	117.1
Xfba276	2A	117.1
Xfbb353b	2A	117.1
Xbarc353	2A	117.1
Xwmc819	2A	117.3
M60/P64.6	2A	118.3
Xwmc261	2A	118.3
Xcfa2263	2A	118.6
Xbarc5	2A	118.9
Xcfa2058	2A	119.6
Xfbb353c	2A	119.6
Xgpw2206	2A	119.6
Xcfd6	2A	120.4
wPt-8071	2A	120.4
XksuG5b	2A	120.6
Xgwm47	2A	121.6
Xgwm445	2A	123.2
Xgwm817	2A	123.8
wPt-5865	2A	124.2
PmHNK54	2A	125.7
Xwmc109	2A	126.6
wPt-3244	2A	129.1
wPt-7056	2A	129.5
Xfba61	2A	130

Xfba62	2A	130
Xfba64	2A	130
Xfbb284	2A	130
XgbxG214	2A	130
Xwmc170	2A	130
Xgwm312	2A	130
wPt-2087	2A	131.5
PPO18	2A	132.2
Ppo-A1	2A	132.2
Xgwm372c	2A	132.2
Xgwm294	2A	132.2
Xcfd168	2A	141.1
Xcfd86	2A	147
Xfba345	2A	149.3
Xcfa2121	2A	150.3
Xgwm47c	2A	151.3
Xbcd1095	2A	152.3
Xbcd1307	2A	152.3
Xbcd1970	2A	152.3
XksuE16	2A	152.3
Xwmc35	2A	153.3
P33/M76-3	2A	154.3
Xpsr934	2A	154.3
Xgpw2229	2A	155.3
Xgpw2281	2A	155.3
XksuD22	2A	155.3
Xwmc181a	2A	155.3
Xbcd1086	2A	156.3
Xbcd292a	2A	157.3
Xcdo1410	2A	161.3
Xglk594	2A	161.3
P36/M41-2	2A	162.3
Xbarc353.1	2A	162.3
Xgpw2046	2A	163.3
Xfba314a	2A	164.3
Xgwm356	2A	164.3
Xfba385a	2A	166.3
XksuF11	2A	166.3
XksuD23	2A	167.3
XksuG5	2A	167.3
Xgwm265	2A	168.3
Xgwm526	2A	168.3
Pm4a	2A	169.3
P78/M87.6	2A	171.3
XksuF41	2A	171.3

Xwg645	2A	171.3
P78/M43.8	2A	173.3
P78/M71.7	2A	173.3
XWC0050pb	2A	173.3
Xbcd18	2A	173.3
Xcdo678	2A	173.3
M77/P64.7	2A	175.3
XksuF1	2A	176.3
Xbcd292	2A	177.3
Xgwm761	2A	177.9
Pm23(Pm4c)	2A	178
Xgwm526a	2A	178
Xcfd168a	2A	178.4
Xwmc198	2A	178.8
wPt-7024a	2A	178.8
Xbarc279	2A	178.8
Xcfd86a	2A	178.9
Xbcd1428	2A	179.7
Xbcd410	2A	179.7
XksuH16	2A	179.7
Xwmc181	2A	181.7
tPt-3136	2A	182.2
wPt-6064	2A	183
Xgwm1067	2A	183.2
Pm4d	2A	184.3
Xbarc76	2A	184.6
wPt-0638	2A	185.3
wPt-3728a	2A	185.3
Xwmc658	2A	185.4
Xgwm382	2A	185.7
wPt-7466	2A	185.7
Xgwm311	2A	187.3
Pm4b	2A	188
XgbxG303	2A	188.4
Xfba209	2A	189.3
Xfba385	2A	189.3
Xgpw4456	2A	189.4
XteLL	2A	190.3
Xcfd267	2A	191.7
XksuF11b	2A	192.3
Xfba8	2A	193.3
Xmag3579	2A	195.4
Xfba314	2A	199.3
Xpsr540	2A	201.9
Xcfa2086	2A	204.3

wPt-4026	2B	0
Lr16	2B	0.3
wPt-0746	2B	2.8
wPt-1663	2B	3.2
wPt-8970	2B	3.2
wPt-3388	2B	3.4
wPt-6158	2B	3.8
wPt-3565	2B	4
wPt-0477	2B	4.1
wPt-6223	2B	4.7
wPt-1813	2B	4.8
W01T03a	2B	5.2
Xbcd348	2B	5.4
Xglk302.1	2B	5.4
Xgwm5b	2B	6.5
wPt-9274	2B	7.1
wPt-1634	2B	7.6
Xwmc764	2B	8.3
wPt-1842	2B	9.8
BQ170801	2B	10
Xbarc45	2B	17.1
Xbarc35	2B	17.2
wPt-7970	2B	19
wPt-5960	2B	19.3
Xwmc661	2B	19.4
wPt-6805	2B	19.7
Xgwm210	2B	20.5
wPt-0100	2B	20.5
wPt-4916	2B	20.5
wPt-3517	2B	20.8
Xwmc489	2B	21.1
wPt-3459	2B	21.9
wPt-8737	2B	22
Xwmc382	2B	22.8
wPt-4453	2B	23.4
wPt-8235	2B	24.2
wPt-1601b	2B	25.6
wPt-2106	2B	25.6
wPt-5738	2B	25.9
XksuD18	2B	27.2
Xcdo456	2B	30.2
Xcmwg682	2B	30.2
Xfba82	2B	30.2
Xmwg682	2B	30.2
Xgwm614	2B	30.2

Sm1	2B	32.2
wPt-7995	2B	32.6
Xfbb171	2B	33
Xgdm93a	2B	33
wPt-10908	2B	33.1
XWB124pbc	2B	33.9
BJ227727	2B	34.6
Xmwg676	2B	34.7
wPt-5188	2B	35.6
wPt-6311	2B	35.6
Xbarc124	2B	35.9
wPt-6706	2B	36
Xfba70	2B	36.6
Xopx9-970	2B	36.6
P78/M59.1	2B	37.6
Xcdo447	2B	37.6
Xfba83	2B	37.6
P78/M68.2	2B	38.6
P78/M68.3b	2B	38.6
P78/M72.8	2B	38.6
Xgpw2039	2B	38.6
wPt-9958	2B	38.9
wPt-5567	2B	40.1
Xcfd238	2B	42.7
CA695634	2B	44
MlIW170	2B	45.8
Xopab2-1410	2B	45.9
wPt-1041	2B	47.1
XE1M1g	2B	47.7
Xfbb274.1	2B	47.7
Xfba308	2B	50.7
Xwmc83	2B	53.7
M86/P65.1	2B	55.7
Xbarc318	2B	56.7
Xwmc25	2B	58.7
wPt-8326	2B	60.6
wPt-8389	2B	60.9
wPt-8004	2B	61
Xgwm154	2B	62
wPt-10778	2B	62.4
Xfba280	2B	62.4
Xwmc154	2B	62.4
wPt-3569a	2B	63.9
Xwmc243	2B	64.4
Xgpw1099	2B	64.9

Xgwm1128	2B	68.1
Xwmc597	2B	68.3
Xwmc213	2B	69.6
tPt-4627	2B	70.6
wPt-5374b	2B	71.4
Xbarc200	2B	71.9
Xbcd18	2B	73.9
Xcfd11b	2B	74.9
Xrz69	2B	74.9
P78/M71.1	2B	75.9
Xgbx3793	2B	76.9
XWB007i	2B	77.9
Xfbb121	2B	77.9
Xfba4.1	2B	78.9
Xfbb40.2	2B	78.9
Xpsr593	2B	78.9
XgbxG035	2B	79.9
Xgwm257	2B	79.9
wPt-6246	2B	80.3
Xmag3976	2B	81.1
P78/M87.5	2B	81.5
Xbcd1434	2B	81.5
Xpsr126	2B	81.5
Xwmc257	2B	82
wPt-7695	2B	82.1
wPt-4301	2B	83.4
wPt-5707b	2B	84.7
wPt-3983b	2B	84.8
wPt-4997	2B	85
wPt-1489	2B	85.1
wPt-3561	2B	85.1
wPt-8072b	2B	85.1
wPt-9402b	2B	85.1
wPt-9423b	2B	85.1
QYld.crc-2B	2B	85.4
Xrz444.1	2B	85.4
Xwmc420	2B	85.4
Xgwm71	2B	85.4
Xbcd1184	2B	86.4
Xpsr146	2B	86.4
wPt-9668	2B	87.7
wPt-6932	2B	87.9
Xfba29a	2B	88.3
Xgpw1148	2B	88.3
Xgwm55a	2B	88.3

Xfba272.2	2B	89.3
Xfba29b	2B	91.3
Xfbb62	2B	91.3
Xwmc422	2B	91.3
Xcdo665	2B	92.3
Xgpw1030	2B	92.3
XgbxG142	2B	93.3
Xbcd445a	2B	94.3
Xcnl6a	2B	94.3
XgbxGx71	2B	94.3
XgbxR452	2B	94.3
Xgwm257b	2B	94.3
Xgwm388a	2B	94.3
Xgwm429	2B	96.3
Xwmc770	2B	98.5
Xbarc10	2B	98.6
Xgwm682	2B	102.3
wPt-4195	2B	102.7
wPt-5788	2B	102.9
wPt-5513	2B	103
Pm42	2B	103.1
wPt-5672	2B	103.9
Xmag464	2B	106.2
wPt-9644	2B	106.2
wPt-2600	2B	106.4
wPt-7320	2B	106.9
wPt-8513	2B	107
wPt-5556	2B	107.1
wPt-0408	2B	107.6
wPt-7158	2B	107.6
Xgwm148	2B	107.7
wPt-6567	2B	107.7
wPt-4125	2B	108.3
TC82742	2B	108.8
wPt-1064	2B	108.8
wPt-6192	2B	108.8
wPt-6199	2B	108.8
wPt-9098	2B	108.8
wPt-0615	2B	109
wPt-7757	2B	109
XgbxR739	2B	109.1
YrCN19	2B	109.1
Xgwm410	2B	109.1
Xwmc597b	2B	109.2
wPt-2120b	2B	109.9

wPt-6477	2B	111
Xfbb353	2B	111.5
Xmwg950	2B	111.5
Xgwm132	2B	111.7
Xgwm128	2B	111.9
Xfba374	2B	112.2
Xtam72.3	2B	112.2
Xwsu1	2B	112.2
Xwmc35	2B	112.2
wPt-3288	2B	112.4
Xpsr596b	2B	112.6
Xwmc434	2B	112.6
wPt-2314	2B	112.9
QEet.inra-2B	2B	113
QYr.sgi-2B.1	2B	113
XgbxG520	2B	113
Xgpw1109	2B	113
Xbarc13	2B	113
Xbarc7	2B	113.5
wPt-7779	2B	113.9
Xbarc183	2B	114.1
Xcdo405	2B	114.4
Xfba106	2B	114.4
Xgpw1073	2B	114.4
Xgpw2225	2B	114.4
Xgpw320	2B	114.4
Yr27	2B	114.4
wPt-6471	2B	115
Xcfa2043	2B	115.4
Xwsu1.1(Dor6)	2B	115.4
wPt-0335	2B	115.8
TC71236	2B	115.9
Xbarc55	2B	116.4
Xwmc261	2B	117
Xfbb47	2B	117.4
Xwmc272	2B	117.6
Ne2	2B	118.4
Xbarc361	2B	118.4
Xbcd152	2B	118.4
Xgwm630	2B	119.4
Xfba38	2B	120.6
Xbarc98	2B	120.7
Xwmc474	2B	120.9
Xbcd265	2B	121
Xbarc1147	2B	121.2

Xfbb185	2B	121.3
Xbarc160	2B	121.6
Xbarc91	2B	121.6
Xbcd1709	2B	121.6
XksuF11a	2B	121.6
Xbarc18	2B	121.6
Xcnl2	2B	122.6
Xfbb21	2B	122.6
Xfbb226a	2B	122.6
Xfbb75a	2B	122.6
Xtam18	2B	122.6
Xwmc179	2B	122.6
Xwmc344	2B	123
Xbarc167	2B	123.2
Xwmc27	2B	123.2
QLr.osu-2B	2B	124.2
Xfba64	2B	124.2
XksuD22	2B	124.2
Xgwm374	2B	124.2
Xwmc265	2B	125.3
Xbcd1119	2B	125.6
Xcdo388	2B	125.6
Xgwm319	2B	125.6
Qfhs.inra-2B	2B	126.6
Xfba199	2B	127.6
Xfbb335	2B	127.6
Xfbb4	2B	127.6
Xcfa2278	2B	127.8
Xbarc349	2B	128.5
Xgwm403	2B	128.5
Xbcd445b	2B	128.8
Xcnl6b	2B	128.8
XksuF37	2B	128.8
Xwmc477	2B	128.8
Sr36	2B	129.8
Xbcd260	2B	129.8
Xcdo370	2B	129.8
XgbxG553	2B	129.8
Xwmc245	2B	130.8
Xwmc592	2B	131.1
Xwmc498	2B	131.3
wPt-6278	2B	131.4
Xgwm271	2B	131.5
Xgwm55	2B	132.8
Xgwm55b	2B	133.7

Xbarc128	2B	134.2
wPt-0473	2B	134.7
Xgwm129	2B	135.4
Xbarc1139	2B	135.8
XWG996	2B	137.3
Xgwm388	2B	137.3
Xgwm337	2B	137.4
CA594434a	2B	138
Xglk594	2B	138.5
QGw1.inra-2B	2B	139.5
Xopp2-510	2B	139.5
BM134420-239	2B	139.5
wPt-0950	2B	139.7
Xfbb284a	2B	140.5
wPt-3132	2B	140.6
wPt-2854	2B	140.7
wPt-7404	2B	140.7
wPt-7200	2B	140.8
Xfba345	2B	141.5
Xfba61	2B	141.5
Xfba62	2B	141.5
XgbxG329	2B	141.5
Xfbb324	2B	142.5
Yr5	2B	142.5
wPt-0694	2B	142.8
Xbarc101	2B	143.5
Xbcd1779	2B	143.5
XksuF15	2B	143.5
Xwsu2(Pk)	2B	143.5
wPt-9736	2B	144.3
CA724675	2B	144.9
Xcdo669	2B	145.1
Xwmc363	2B	145.6
Xwmc441	2B	145.6
Xcfd70c	2B	145.8
wPt-4968	2B	145.8
Xwmc149a	2B	146.4
Xcfd70	2B	146.7
Xwmc500	2B	147.2
wPt-1140	2B	147.2
wPt-5878	2B	147.7
Xwmc51	2B	148.4
wPt-4002	2B	148.4
wPt-4199	2B	148.6
wPt-5128	2B	148.6

Xcdo684	2B	148.9
Xgwm120	2B	148.9
Xgwm1027	2B	149
wPt-7892	2B	149.2
wPt-8040a	2B	149.7
Xgwm191	2B	149.7
Xmxe3M2b	2B	150.7
Xwmc51h	2B	150.7
BU099658	2B	150.8
wPt-3701	2B	150.8
wPt-4812	2B	151.5
Xgwm877	2B	151.8
tPt-6105	2B	151.9
wPt-7765	2B	151.9
wPt-7859	2B	151.9
wPt-7161	2B	152
wPt-8856	2B	152.2
wPt-5736	2B	152.3
wPt-7350	2B	152.4
wPt-8284	2B	152.4
wPt-9336	2B	152.4
wPt-6565	2B	152.5
Xfbb113a	2B	152.8
wPt-10715	2B	153
wPt-2397	2B	153
Xbcd292	2B	153.2
wPt-1650	2B	153.4
Xfba209	2B	153.8
Yr7	2B	153.8
Xwmc360	2B	153.8
wPt-3042	2B	153.8
wPt-305355	2B	153.9
wPt-6522	2B	154.4
wPt-8569	2B	154.5
Xcfd73	2B	154.7
wPt-3436	2B	154.7
wPt-305036	2B	154.9
wPt-7317	2B	155.1
wPt-0906	2B	155.8
wPt-0794	2B	156.2
wPt-0891	2B	156.6
Xmwg2025	2B	156.7
wPt-2274	2B	157.1
rPt-6122	2B	157.3
Xbcd512	2B	157.4

wPt-11421	2B	157.4
Xbcd307	2B	157.6
Xgwm16	2B	157.6
wPt-3632	2B	157.6
wPt-6643	2B	157.6
Xgwm47	2B	157.8
wPt-2724	2B	158.2
wPt-10548	2B	158.8
Xgwm130	2B	159.1
wPt-2293	2B	159.2
wPt-8693	2B	159.4
wPt-8340	2B	160
Xwmc435	2B	160.4
wPt-0189	2B	160.5
wPt-9654	2B	160.6
wPt-1294	2B	160.7
CA662535	2B	160.8
Xbcd410	2B	161
Xpsr370b	2B	161.2
Xfba359a	2B	162.1
Xgwm501	2B	162.1
wPt-8521	2B	163.2
wPt-2135	2B	163.3
wPt-10440	2B	163.5
wPt-3378	2B	163.5
wPt-5779	2B	163.5
wPt-7360	2B	163.5
wPt-5680	2B	163.6
wPt-3929	2B	163.7
wPt-0489	2B	163.8
TC72953	2B	164
wPt-3651	2B	164
wPt-0049	2B	165.8
wPt-4368	2B	165.9
wPt-4917	2B	165.9
Xwmc175	2B	166.1
wPt-2266	2B	166.6
wPt-1646	2B	166.8
wPt-7305	2B	166.8
Xwmc175a	2B	166.9
wPt-5242	2B	168.6
Xgwm1300	2B	168.7
BJ253815	2B	169.3
Sr9a	2B	170.2
Xcdo678a	2B	170.2

Xfba117	2B	170.2
Xfba276	2B	170.2
CA681959b	2B	171.6
Xmwg660a	2B	174.3
XpKabaG	2B	174.3
Xwmc332	2B	174.3
Xbcd1095A	2B	175.3
Xbcd135	2B	175.3
Xfba385a	2B	175.3
Xfba102	2B	176.3
Xfba116	2B	176.3
XgbxG087	2B	176.3
XksuF1	2B	176.3
XksuF41	2B	176.3
Xmwg546	2B	176.3
Xcfd267	2B	177.3
Xwmc434a	2B	177.3
Xglk558	2B	180.3
XksuD23	2B	181.3
Xfba314	2B	182.3
Xcdo36	2B	183.3
Xfba310	2B	183.3
Xfbb284b	2B	183.3
Xcdo244	2B	184.2
Xfbb226b	2B	184.2
Xfbb75b	2B	185.2
XksuF11b	2B	185.2
Xwg645	2B	185.2
Xwmc627	2B	185.3
wPt-9257	2B	185.3
wPt-4223	2B	185.7
wPt-0510	2B	186.6
wPt-0567	2B	187.5
Xbcd1231	2B	188.5
Xfbb278	2B	188.5
Xgwm619	2B	190.5
Xgdm93b	2B	191.5
Xksu558	2B	192.5
Lr50	2B	193.5
wPt-8776	2B	193.8
Xcdo678b	2B	194.3
Xfba359b	2B	195.3
Pm33	2B	196.3
Xfba385b	2B	196.3
Xwmc149	2B	196.3

TC89976	2B	196.5
Xwmc361	2B	197.6
wPt-7506	2B	199.1
wPt-2622	2B	201.1
PmJM22	2B	201.6
Rht4	2B	204.1
Xwmc317	2B	204.1
Xwmc817	2B	204.8
wPt-6894	2B	204.9
wPt-0471	2B	205.3
Xcmwg660	2B	205.7
Xmwg660b	2B	205.7
PmPS5B	2B	205.9
MlAB10	2B	206.1
Xbarc159	2B	208.7
Xwg338	2B	209.1
Xfbb113	2B	209.4
Xwmc445	2B	209.4
Xgwm382	2B	215.7
MlZec1	2B	218.3
Xwmc356	2B	218.9
Xgwm526	2B	222.4
Xwmc602	2B	225.9
Pm6	2B	263.3
XW21a	2D	0
XWB124pba	2D	0
Xcfd77	2D	5
Xbarc124	2D	8
Iw2	2D	9
D1.1_ctg10347	2D	10
D1.1_ctg13285	2D	10
D1.1_ctg1672	2D	10
D1.1_ctg4187	2D	10
D1.1_ctg5156	2D	10
D1.1_ctg5751	2D	10
D1.1_ctg5966	2D	10
D1.1_ctg7416	2D	10
D1.1_ctg8	2D	10
D1.1_ctg9710	2D	10
Xfbb274	2D	10
D1.1_ctg5649	2D	11
Xfba83a	2D	11
XgbxG218b	2D	11
Xgwm37	2D	11
XksuE3b	2D	11

Xwmc818	2D	11.1
Xwmc574	2D	11.4
Xcfa2040	2D	12.1
Xcmwg682a	2D	12.1
Xfba349a	2D	12.1
Xfbb189b	2D	12.1
XgbxG747a	2D	12.1
Xcfd56	2D	13.7
Xcfd65	2D	13.7
Xcfd51	2D	16.4
Xbarc90	2D	17.3
D1.1_ctg3038	2D	18
QEet.inra-2D	2D	18
Xbcd18a	2D	18
Xcdo456a	2D	18
Xgpw294	2D	18
Xmwg682	2D	18
Xcfd36	2D	18
Xwmc111	2D	18.3
Xbcd1970	2D	19.3
wPt-6419	2D	19.5
Lr39	2D	20.5
Xfba272	2D	20.5
Xwmc25	2D	23.8
Xwmc503	2D	25.8
Xgwm261	2D	28.5
P39/M49-121	2D	29.1
Xgwm296	2D	29.1
Xgwm210	2D	30.9
Xcfd11	2D	31.9
Xcfd53	2D	32.9
Xwmc112	2D	33.6
Xabc165a	2D	33.8
XksuA1a	2D	33.8
wPt-2644	2D	33.8
P32/M37-6	2D	33.8
wPt-9997	2D	33.9
D1.1_ctg9144	2D	34.8
Xpsr908	2D	34.8
Xwmc431	2D	34.8
QFhs.pur.2D	2D	35.8
Xfba400	2D	35.8
Rht8	2D	36.8
Xgpw332	2D	36.8
Xcdo366	2D	37.7

QHd.ksu-2D	2D	37.7
P37/M39f	2D	38.7
Xfba349b	2D	38.7
Xfba88	2D	38.7
Xfba4	2D	39.7
Xgwm455	2D	39.7
Xbarc297	2D	40.5
wPt-8319	2D	41.4
Tg1	2D	41.6
wPt-3202	2D	43.2
wPt-6343	2D	43.2
Xcdo1379	2D	44.6
Xwmc470	2D	44.6
Xpsr370a	2D	45.1
Xbcd611	2D	45.9
Xwmc087	2D	45.9
wPt-0298	2D	46.9
wPt-2781	2D	46.9
wPt-6003	2D	46.9
wPt-7375	2D	46.9
Xgwm122	2D	48.6
Xgwm484	2D	48.6
D1.1_ctg161	2D	49.6
D1.1_ctg4862	2D	49.6
Xbcd102	2D	49.6
Xcfd255	2D	49.6
Xcdo1479	2D	50.6
Xwmc453	2D	50.6
wPt-9749	2D	50.6
Xcfd43	2D	50.8
wPt-0619	2D	53.1
Xwmc190	2D	54
wPt-4144	2D	54
Xbarc168	2D	54.3
Xgwm102	2D	55.8
D1.1_ctg7448	2D	56.4
Xbcd262	2D	56.4
Xfbb279	2D	56.4
wPt-1991	2D	57
Xbcd718	2D	57.6
Xwsu1	2D	58.6
Xwsu1.2(Dor6)	2D	58.6
Xbcd1970b	2D	59.6
Xcfd2	2D	59.6
BE497718-260	2D	60.6

Xbcd18b	2D	60.7
Xcdo405	2D	60.7
Xcfa2262	2D	60.7
Xfba83	2D	60.7
wPt-8524	2D	61.7
Xgpw361	2D	62.6
Xgwm515	2D	62.7
Xcdo456b	2D	63.4
Xfba65	2D	63.4
XgbxG212	2D	63.4
Xgwm515b	2D	63.4
XgbxG536	2D	64.4
Xbarc292	2D	65.4
Xfba38	2D	65.4
XgbxR618	2D	65.4
Xgpw346	2D	65.4
P78/M72.4a	2D	66.4
Xgpw2088	2D	66.4
Xgpw349	2D	66.4
Xbcd260	2D	68.4
Xcmwg682	2D	68.4
Xgwm249	2D	69.4
Xw2Ib	2D	70.4
XgbxR635	2D	71.4
D1.1_ctg216_1	2D	73.4
Xfba74a	2D	73.4
XgbxG145	2D	73.4
Xtam8a	2D	73.4
P35/M48-4	2D	74.4
Xbcd120	2D	75.4
XksuA1b	2D	75.4
Xwmc18	2D	75.4
Acph-D2	2D	76.2
D1.1_ctg6265-1	2D	76.2
Xbcd111	2D	76.2
Xfba341a	2D	76.2
Xwmc149	2D	77.2
Xbarc11	2D	78.2
Xgwm30	2D	82.2
Xcfd17	2D	83.3
Xgwm358	2D	83.6
Xbarc145	2D	84.2
Xcfd116	2D	84.4
Xwmc630	2D	85
Xwmc245	2D	85.8

Xwmc144	2D	85.9
Xcfd160a	2D	87.6
Xcfd193	2D	87.6
Xcfd160	2D	87.8
Xwmc601	2D	87.9
Xfbb99	2D	88.8
Xwmc797	2D	90
wPt-1554	2D	90
Xgwm157	2D	92
Xgwm539	2D	94.8
Xcfd73	2D	96.1
Xcfd73b	2D	97.3
Xcfd233	2D	98.3
wPt-0330	2D	98.9
Xcfd62	2D	99.3
Xbarc228	2D	100.2
wPt-6574	2D	101
wPt-6752	2D	101.3
Xgwm296b	2D	101.9
Xwmc41	2D	102.2
Xwmc181	2D	104.1
Xcfd16	2D	104.4
Pm43	2D	104.6
wPt-3728b	2D	105.3
Xcfd168b	2D	107.4
Xcfd270	2D	107.4
D1.1_ctg5879	2D	109.4
Xfbb164b	2D	109.4
Xgpw1184	2D	109.4
Xgpw1247	2D	109.4
XgbxG575b	2D	110.4
D1.1_ctg216-2	2D	111.4
Xfba74b	2D	111.4
Xfba111a	2D	112.4
D1.1_ctg4236_1	2D	113.4
D1.1_ctg6265_2	2D	113.4
Xfba341b	2D	113.4
Xfba61a	2D	113.4
Xfba62a	2D	113.4
Xfba64a	2D	113.4
Xfbb122a	2D	113.4
Xfbb284a	2D	113.4
Xgwm790	2D	113.4
Xfbb32a	2D	114.4
Xtam8b	2D	115.4

Xwmc035a	2D	115.4
Xgwm608	2D	116.4
Xpsr331.1	2D	116.4
Xfbb68a	2D	117.4
Xmwg526a	2D	117.4
Xrz444a	2D	119.4
D1.1_ctg6289_1	2D	120.4
Xfba209a	2D	120.4
Xfbb9a	2D	120.4
D1.1_ctg5940_1	2D	121.4
D1.1_ctg839_1	2D	121.4
Xfbb377a	2D	121.4
D1.1_ctg8097_1	2D	122.4
Xcfd44	2D	122.4
Xfba116a	2D	122.4
Xcfd168	2D	122.6
Xwmc243	2D	124.2
M86/P65.3	2D	124.3
Xcdo1008	2D	124.3
M51/P65.0	2D	125.3
Xgbx3832a	2D	125.3
Xgwm349	2D	127.7
Xksud23a	2D	128.7
Xcfd239	2D	128.8
D1.1_ctg3738_1	2D	129.5
Xfba311a	2D	129.5
Xfbb251a	2D	129.5
Xgwm877.2	2D	129.5
Xfbb72a	2D	131.5
Xksu558	2D	131.5
Xcfd161	2D	132.5
Xwmc445	2D	132.5
D1.1_ctg6265	2D	134.5
Xfba341c	2D	134.5
Xfba61b	2D	134.5
Xfbb284b	2D	134.5
D1.1_ctg4236_2	2D	135.5
D1.1_ctg8097_2	2D	135.5
Xfba111b	2D	135.5
Xfba62b	2D	135.5
Xfba64b	2D	135.5
Xfbb122b	2D	135.5
Xfbb32b	2D	135.5
Xgpw2321	2D	135.5
Xbarc219	2D	137.5

Xgpw308	2D	137.5
wPt-4413	2D	138.4
Xgpw313a	2D	138.4
Xgpw300	2D	139.4
Xgpw338	2D	139.4
Xgwm301a	2D	139.4
Xrz444b	2D	139.4
Xgpw354	2D	140.4
XWC0050pa	2D	141.4
Xgwm311.1	2D	141.4
Xmwg526b	2D	141.4
Xwmc167	2D	142.4
Xwmc175	2D	144.5
Xgwm382	2D	145.7
D1.1_ctg3434	2D	146.7
D1.1_ctg5940_2	2D	146.7
D1.1_ctg839_2	2D	146.7
XBCD410	2D	146.7
Xfbb377b	2D	146.7
P35/M53-3	2D	147.7
P38/M49-7	2D	147.7
P40/M51-1	2D	147.7
P41/M48-61	2D	147.7
P41/M61-3	2D	147.7
Xgwm320	2D	148.7
D1.1_ctg2905	2D	149.2
D1.1_ctg7196	2D	149.2
XksuH9	2D	149.2
Xbarc59	2D	149.2
Xbarc159	2D	149.9
Xwmc817	2D	151.9
Xglk558	2D	152.6
Xfbb68b	2D	154.6
Xgwm349b	2D	154.6
XksuD23b	2D	154.6
Xgwm301	2D	154.6
Xcfd50	2D	155.6
Xfba314.2	2D	156.6
P31/M54-4	2D	158.6
XksuH16	2D	158.6
D1.1_ctg6289_2	2D	161.6
D1.1_ctg8097	2D	161.6
Xfba116b	2D	161.6
Xfbb9b	2D	161.6
P42/M60-6	2D	162.6

Xfbb72b	2D	162.6
Xcdo18	2D	163.6
Xcdo36	2D	163.6
Xfba311b	2D	168.6
D1.1_ctg3738_2	2D	169.6
Xfbb251b	2D	169.6
Xabc165b	2D	174.3
Xfba209b	2D	180.6
Xbarc57	3A	0
wPt-7217	3A	14.3
P41/M61-1	3A	17
Xsun2.2	3A	17
P31/M57-2	3A	18
P41/M51-322	3A	18
Xgpw1106b	3A	18
Xksu683	3A	18
P36/M54-6	3A	19
P39/M49-377	3A	19
XgpwE11c	3A	19
Xpsr311a	3A	20
Xfbb370	3A	21
Xglk683	3A	21
Xbarc294	3A	22
Xbarc321	3A	22
Xbarc310	3A	23
Xwmc11	3A	23
wPt-1816	3A	24.9
wPt-2813	3A	24.9
wPt-8753	3A	24.9
Xabc172a	3A	26.1
Xbcd15	3A	26.1
QPhs.ocs-3A.1	3A	27.7
Xwmc532	3A	28.6
Xfbb293a	3A	29.1
Xfbb237a	3A	31.1
Xmwg11	3A	31.1
Xtam47.1	3A	31.1
Xcfd79	3A	32.5
Xbcd1773a	3A	33.1
Xmwg12a	3A	33.1
P37/M58-165	3A	34.1
Xtam61.2	3A	34.1
Xcdo395	3A	36.1
Xgwm369	3A	36.1
Xbcd1428	3A	37.1

Xcdo482	3A	38.1
Xtam63a	3A	38.1
P41/M41-4	3A	39.1
Xfba91	3A	39.1
wPt-9369	3A	39.7
Xbcd22	3A	40.6
wPt-7608	3A	40.6
Xgwm218	3A	41.7
Xbarc284	3A	42.7
Xfba366	3A	42.7
Xgpw2142b	3A	42.7
Xbcd1278	3A	44.7
Xcdo718A	3A	44.7
Xgpw2132	3A	44.7
P37/M58-3	3A	45.7
wPt-6854	3A	50
Xabc172b	3A	51.8
P35/M55-6	3A	52.8
P35/M50-1	3A	53.8
Xwmc050	3A	53.8
Xbarc179	3A	54.8
Xfbb237b	3A	54.8
Xmwg11b	3A	54.8
P36/M53-134	3A	55.8
Xmwg12	3A	55.8
Xmwg14	3A	56.8
Xfba091	3A	57.8
wPt-304671	3A	58.9
P42/M50-1	3A	59.7
XksuA6	3A	59.7
Xgpw2118b	3A	60.7
P32/M59-7	3A	61.7
Xcdo638	3A	61.7
XgbxG406	3A	61.7
tin3	3A	61.7
Br-A1	3A	62.7
Xgpw2016	3A	62.7
XksuA6.1	3A	62.7
wPt-6204	3A	62.9
wPt-9049	3A	62.9
P37/M53-3	3A	63.4
XWC2163ea	3A	63.4
Xpsr930	3A	63.4
Xpsr598	3A	63.4
Xcdo1345	3A	64.4

Xfbb293b	3A	64.4
wPt-1681	3A	64.5
wPt-4143	3A	64.5
wPt-7756	3A	64.8
P42/M49-2	3A	65.4
Xabg471.1	3A	65.4
Xbcd1823	3A	65.4
wPt-2698	3A	65.8
Xcdo1164	3A	66.1
Xpsr903a	3A	66.1
P41/M40-1	3A	67.1
Xbcd706	3A	67.1
Xmwg22.1	3A	67.1
Xbcd1532	3A	68.1
Xcdo1435	3A	68.1
Xabg460	3A	69.1
Xgpw297	3A	69.1
P40/M61-3	3A	70.1
Xpsr903b	3A	70.1
Xbarc356	3A	70.1
P39/M50-152	3A	71.1
XksuB8	3A	71.1
Xgpw2169	3A	72.1
Xgwm2	3A	72.1
BJ213673c	3A	73.1
Xbarc19	3A	73.1
Xbarc324	3A	73.1
Xgpw2266	3A	73.1
Xbarc45	3A	73.1
wPt-9562	3A	73.3
wPt-4352	3A	73.6
P44/M61-4	3A	73.8
Xcdo54	3A	73.8
Xfba127.1	3A	73.8
wPt-2755	3A	74.8
P37/M39-4	3A	74.8
Xbcd1145	3A	74.8
Xfba175	3A	74.8
Xbcd1127	3A	75.8
Xpsr311b	3A	75.9
wPt-7992	3A	75.9
Xgwm133	3A	76.1
wPt-1596	3A	76.5
wPt-2938	3A	76.5
S-A1	3A	76.8

Xcdo718B	3A	76.8
Xfbb332	3A	76.8
Xwmc379	3A	76.8
Xwmc50	3A	77.3
wPt-9154	3A	77.9
Xwmc505	3A	78.1
Xabg396	3A	78.2
Xbcd366	3A	78.2
Xbcd828	3A	78.2
wPt-1036	3A	78.2
Xgwm720	3A	78.9
wPt-2967	3A	79.3
wPt-5486	3A	79.9
Xgwm32	3A	80.1
Xwmc640	3A	80.2
W01T03c	3A	80.3
wPt-0714	3A	80.3
Xmag620b	3A	80.6
Xcfa2164b	3A	80.7
Xgwm156	3A	81.1
Xwmc664	3A	81.2
Xgwm5	3A	81.6
Xgwm4	3A	81.7
Xgwm666a	3A	82
Xgwm674	3A	82.6
Xgwm30	3A	83.1
Xwmc651	3A	83.2
Xbarc67	3A	84.2
Xcdo1174	3A	84.2
Xmwg802	3A	84.2
Xwmc388a	3A	84.4
Xcfd193	3A	85
Xgwm403	3A	86.2
Xwmc627	3A	86.2
Xwmc489	3A	86.2
Xcfa2134	3A	88
wPt-5886	3A	88.9
Xcfa2234	3A	89
XATPase	3A	89.4
Xcdo118	3A	89.4
Xwmc527	3A	89.5
Xgwm1042	3A	89.5
Xwmc695	3A	90.1
Xwmc269	3A	91.3
Xwmc527b	3A	91.3

Xwmc695a	3A	91.3
Xwmc269b	3A	92.3
Xwmc428	3A	94.3
Xdupw227	3A	94.5
TC74823a	3A	95.1
wPt-6891	3A	96.4
wPt-7890	3A	96.4
Xwmc264	3A	99.3
wPt-9215	3A	101.7
P35/M54-5	3A	102.3
P42/M49-3	3A	102.3
Xcfa2262	3A	102.3
wPt-5084	3A	102.3
wPt-4859	3A	102.6
wPt-4939	3A	102.7
wPt-11619	3A	102.9
wPt-2202	3A	103
wPt-6218	3A	103.6
wPt-5786	3A	104.2
Xgwm494	3A	104.3
Xbarc25	3A	104.8
XgbxG034	3A	104.8
Xgwm162	3A	105.3
F129	3A	105.4
QHt.riso-3A	3A	106
Xfbb277.2	3A	106
Xwmc286	3A	106
wPt-6422	3A	106.9
Xcdo281	3A	107.1
XksuH2	3A	107.1
Xwg177	3A	107.1
tPt-7209a	3A	107.7
wPt-5943a	3A	107.7
Xgwm1038	3A	107.9
Xmwg30a	3A	108.2
Xpsr156	3A	108.2
Xfbb271	3A	109.2
Xwmc96	3A	109.8
Xgwm751	3A	110
Xbcd452	3A	110.2
Xgpw2109	3A	110.2
Xbarc69	3A	110.8
Xgwm497	3A	110.9
Xbcd115	3A	111.3
Xbcd2044	3A	111.3

Xcfa2193	3A	111.7
wPt-4077	3A	112.2
Xmwg961	3A	112.4
Xtam33	3A	112.4
wPt-4725	3A	113.5
Xopx-09.2	3A	113.5
M17/P77.12	3A	115.5
XWC2234c	3A	115.5
Xfbb353.1	3A	115.5
Xpsp3047	3A	115.5
XgbxG499	3A	116.5
wPt-9268	3A	116.5
Xbarc12	3A	117.1
Xwmc173	3A	117.9
Xbcd1145b	3A	118.6
Xtam63b	3A	118.6
Xcfd2	3A	118.7
Xwmc559	3A	120
tPt-1002	3A	120.6
Xgwm155	3A	122
Xwmc153	3A	124.2
wPt-3697	3A	125.3
Xwmc215	3A	126.2
wPt-4407	3A	127.3
Xbcd372	3A	129.8
Xcdo460	3A	131
Xfba167.1	3A	132.7
Xabc172c	3A	133.7
Xcfa2076	3A	134.7
Xwmc169	3A	136.3
Xwmc388b	3A	136.9
Xwmc594	3A	142
Xgwm666b	3A	143
wPt-1864	3A	148
wPt-2866	3A	148
P78/M69.3	3A	149.3
wPt-1688	3A	149.9
M71/P77.1	3A	152.9
P78/M69.8	3A	152.9
Xgwm480	3A	152.9
wPt-3816	3A	156
wPt-2659	3A	156.2
tPt-7492	3A	157.4
wPt-5125	3A	157.4
wPt-5133	3A	157.4

Xwmc206	3A	165.9
Xfbb293.1	3A	167.9
wPt-9422	3A	168.8
Xpsr1203.1	3A	170.3
Xbcd1773b	3A	171.3
Xfbb293	3A	171.3
wPt-1694	3A	172.7
wPt-4398	3A	172.7
wPt-9238	3A	172.7
wPt-2492	3A	175.1
QFhs.inra-3A	3A	176.3
Xmwg30b	3A	176.3
Xbarc314	3A	176.3
Xfba347	3A	178.3
Xbarc197.1	3A	179.3
Xgpw2270	3A	179.3
Xgwm391	3A	179.3
TC77302	3A	179.5
Xfbb260	3A	180.3
wPt-3978	3A	181.9
Xbcd1431.1	3A	182.3
Xfbb250	3A	183.3
Xgwm247	3A	183.3
QPhs.occsu-3A	3A	184.3
R-A1	3A	184.3
Xfba242	3A	184.3
Xwg184	3A	184.3
Xfbb322b	3A	185.3
XgbxG242	3A	189.3
Xgwm107	3A	193.3
Xmwg570	3A	193.3
wPt-2144	3A	193.6
wPt-9160	3A	193.6
wPt-0398	3A	196
Xpsr78	3A	197.3
Xcdo113	3A	203.3
Xbcd358	3A	221.3
XE5M5h	3A	225.3
P78/M92.3	3B	0
Xfba189a	3B	2
M21/P76.3	3B	3
P78/M43.11	3B	4
XWB075j	3B	5
XWB133j	3B	5
XWB147j	3B	5

XgpwE11a	3B	5
Xpsr345.1	3B	5
Xpsr913	3B	5
P78/M59.2	3B	6
XE3M3	3B	6
Stb2	3B	7
XMXE8M3m	3B	7
Xbcd907.1	3B	7
Xfbb147a	3B	9
XksuG30a	3B	9
QSng.sfr-3B	3B	10
Sr2	3B	10
Xbarc75	3B	10
Xbarc180	3B	10.2
TC89014	3B	10.5
Xwmc430a	3B	11.4
Xgwm389	3B	11.8
Xwmc674	3B	12.3
Xfba190.2	3B	13.9
Xfbb156a	3B	13.9
Xgwm533a	3B	13.9
Xmag2095	3B	14.4
XksuG53	3B	14.7
Xbarc133	3B	15.7
Xbarc147	3B	15.7
Xfba311	3B	15.7
wPt-7264	3B	16.7
wPt-7225	3B	17.1
wPt-5697	3B	17.3
P78/M69.5	3B	17.4
Xcfd79b	3B	17.4
Lr27	3B	18.4
Xfbb166.1	3B	18.4
Xpsr375.5	3B	18.4
Xpsr567.2	3B	18.4
wPt-10503	3B	20.6
M62/P64.7	3B	20.8
QFhs.ndsu-3BS	3B	20.8
Xgwm493	3B	20.8
F103	3B	21
wPt-10291	3B	21.2
wPt-10388	3B	21.2
Xbarc87	3B	21.5
wPt-6129	3B	21.5
wPt-10239	3B	22

Xcfd79	3B	22.3
Xwmc754	3B	22.3
Xbarc92	3B	23.2
wPt-1516	3B	23.6
Xblt4.1	3B	23.9
Xcdo460	3B	23.9
Xcdo583a	3B	23.9
wPt-1132a	3B	25
Stb14	3B	25.1
Xbcd1418	3B	25.1
Xbcd809a	3B	26.1
Xtam47.2	3B	26.1
Xwmc43a	3B	26.1
wPt-11454	3B	26.3
wPt-10372	3B	26.7
XATPase.1	3B	27.3
Xfbb185	3B	27.3
tPt-1079	3B	27.8
Xbcd1380A	3B	28.6
wPt-7341	3B	29.3
wPt-8855	3B	29.3
P38/M61-4	3B	29.9
Xbcd1278	3B	29.9
wPt-7984	3B	30.2
wPt-8079	3B	30.9
Xglk683/Sun2	3B	31.2
XksuE3	3B	31.2
wPt-10333	3B	31.8
Xtam61.1	3B	32.4
Xmag501	3B	32.7
Xwmc430b	3B	32.8
Xgpw1107	3B	33.5
wPt-2757a	3B	34.2
wPt-1081a	3B	34.3
wPt-3609	3B	34.3
Xwmc597	3B	35.2
Xgwm533b	3B	35.7
wPt-7212	3B	35.7
wPt-3598	3B	35.8
wPt-9410	3B	35.8
wPt-5064	3B	35.9
wPt-0302	3B	36.9
wPt-3536	3B	36.9
wPt-10714	3B	38
Xfbb1851	3B	39

Xcfd28	3B	40
Xwmc623	3B	40.9
Xbarc102	3B	41.5
Xglk538a	3B	41.5
wPt-7132	3B	43
wPt-9012	3B	43
wPt-4653	3B	43.1
Xwmc808	3B	43.5
wPt-9010	3B	45
wPt-4842	3B	45.2
tPt-6487	3B	45.6
wPt-4739	3B	46
Xwmc679	3B	46.1
wPt-6335	3B	46.9
wPt-7860	3B	47.2
wPt-8828	3B	47.6
wPt-10436	3B	47.7
wPt-5432	3B	48.6
Xwmc500	3B	49.1
Xcdo395	3B	49.2
wPt-2961	3B	49.3
wPt-9107	3B	50.1
wPt-6742	3B	51.7
Xwmc43	3B	52.1
Xwmc51	3B	52.4
wPt-0672	3B	53.2
wPt-10005	3B	53.7
wPt-10230	3B	53.7
wPt-10277	3B	53.7
wPt-4431	3B	53.7
wPt-8990	3B	53.7
wPt-9250	3B	53.7
Xwmc78	3B	54.2
XWB068j	3B	55.2
Xgpw1108a	3B	55.2
Xwmc007	3B	55.2
M60/P64.11	3B	56.2
P78/M87.2	3B	56.2
Xgpw1145	3B	56.2
Xgpw1146a	3B	56.2
Xfba91.2	3B	57.2
Xgpw1201b	3B	57.2
wPt-3046	3B	57.7
wPt-8686	3B	57.8
wPt-1406	3B	58.1

M83/P65.10	3B	58.2
Xfba91	3B	58.2
Xwmc540a	3B	58.6
wPt-10052	3B	58.7
wPt-10541	3B	58.7
wPt-10729	3B	58.7
wPt-11103	3B	58.7
wPt-11194	3B	58.7
wPt-11276	3B	58.7
wPt-11443	3B	58.7
wPt-2567	3B	58.7
wPt-2784	3B	58.7
wPt-3410	3B	58.7
wPt-6008	3B	58.7
wPt-7357	3B	58.7
wPt-8643	3B	58.7
wPt-9495	3B	58.7
wPt-1349	3B	58.8
wPt-10958	3B	58.9
Xscu3H03	3B	59
Xwmc85.2	3B	59
rPt-5853	3B	59.1
tPt-8143	3B	59.2
wPt-2013	3B	59.3
wPt-4476	3B	59.3
wPt-5215	3B	59.3
wPt-6066	3B	59.3
tPt-9273	3B	59.4
wPt-4292	3B	59.4
wPt-8592	3B	59.6
wPt-9982	3B	59.6
wPt-5244	3B	59.8
wPt-10540	3B	59.9
wPt-10969	3B	59.9
wPt-8356	3B	59.9
wPt-10389	3B	60
wPt-2720	3B	60
wPt-4777	3B	60
wPt-5495	3B	60
wPt-7677	3B	60
wPt-8126	3B	60
Xbarc218	3B	60
Xgwm144	3B	60
wPt-10420	3B	60.1
wPt-10677	3B	60.1

wPt-9088	3B	60.2
wPt-10552	3B	60.3
Xgwm383	3B	60.4
wPt-0995	3B	60.5
wPt-2403	3B	60.5
wPt-2748	3B	60.5
wPt-5716	3B	60.5
wPt-7015	3B	60.5
wPt-7024b	3B	60.5
wPt-7142	3B	60.5
wPt-7229	3B	60.5
wPt-7266	3B	60.5
wPt-9432	3B	60.5
Xgwm566	3B	60.6
Xfbb24	3B	61.4
Xgpw1120	3B	61.4
Xbarc173	3B	61.5
wPt-9310	3B	61.5
wPt-10080	3B	61.6
wPt-1159	3B	61.7
wPt-2936	3B	61.7
wPt-4209	3B	61.7
wPt-8579	3B	61.7
wPt-9510	3B	61.7
wPt-6239	3B	62.2
Xcfa2241	3B	62.8
wPt-0644	3B	62.8
wPt-5390	3B	63.5
wPt-7614	3B	63.9
Xbarc139	3B	64.2
wPt-1625	3B	64.3
wPt-4597	3B	64.3
Xgwm685	3B	64.7
Xfbb142	3B	65.3
Xpsp3030.1	3B	65.3
M60/P64.5a	3B	66.3
Xgwm156	3B	66.3
Xwmc69	3B	66.3
wPt-9170	3B	66.7
CA499601	3B	70
wPt-6047	3B	70
wPt-9579	3B	70.1
wPt-5105	3B	70.3
wPt-5640	3B	70.4
wPt-7934a	3B	70.4

wPt-2377	3B	70.8
wPt-3760	3B	70.9
Xgwm264	3B	71.2
wPt-0438	3B	71.2
Xwmc231	3B	71.5
Xgwm284	3B	71.8
Xbarc68	3B	72.3
Xgwm72	3B	72.3
Xpsr689	3B	72.3
wPt-6973	3B	72.3
Xwmc815	3B	72.4
Xwmc675	3B	72.5
Xwmc505	3B	72.7
Xcfd6	3B	73.1
Xwmc777	3B	73.1
wPt-2119	3B	73.1
Xwmc612	3B	73.6
Xpsr902	3B	73.7
wPt-0695	3B	73.8
Xwmc625	3B	73.9
XgbxG392	3B	74.1
Xpsp3144	3B	74.1
Xbarc73	3B	74.2
Xwmc693	3B	74.5
Xbcd809	3B	74.6
wPt-5906	3B	74.7
Xwmc544	3B	74.8
Xwmc695	3B	74.8
Xgwm285	3B	75.1
Xwmc446	3B	75.1
Xgwm274	3B	75.3
Xwmc615	3B	75.6
Xwmc366	3B	75.8
Xwmc1	3B	75.9
Xwmc533	3B	76.4
Xwmc762	3B	76.5
Xwmc751	3B	76.7
XksuG59	3B	76.8
Xgwm644	3B	76.9
P40/M54-3	3B	77.5
P78/M69.2	3B	77.5
Xopa10-500	3B	77.5
Xcfd4a	3B	77.5
Xgwm376	3B	77.5
Xcfd4	3B	77.7

XPc-A12a	3B	79.4
Xfbb315	3B	79.4
XgbxR080	3B	79.4
Xpsr903	3B	79.4
Xwmc395	3B	79.4
Xgwm77	3B	79.4
P34/M51-67	3B	80.4
Xabg471	3B	80.4
Xcnl8	3B	80.4
Xbarc187	3B	80.7
Xwmc307	3B	80.8
wPt-6802	3B	80.8
Xbcd1418a	3B	81
P41/M39-1	3B	81
Xcdo1164	3B	81
Xfbb117.1	3B	81
Xpsr156	3B	81
S-B1	3B	81.5
P35/M61-8	3B	82
Xfba189	3B	82
XATPase.2	3B	83
Xcdo328	3B	83
Xgwm888	3B	83
Xwmc527a	3B	83
tPt-0353a	3B	83
rPt-5396	3B	83.1
wPt-4127	3B	83.1
wPt-7502	3B	83.1
Xwmc653	3B	83.2
Xgwm107	3B	83.6
wPt-4082	3B	83.6
Xfbb110	3B	83.8
Xfbb235	3B	83.8
XksuH7	3B	83.8
Xpsr170a	3B	83.8
Xwmc471	3B	84
Xwmc182	3B	84.3
wPt-3327	3B	84.4
P34/M48-5	3B	84.9
P34/M50-10	3B	84.9
P36/M48-6	3B	84.9
Xbcd1283	3B	84.9
Xbcd1380	3B	84.9
Xbcd1380B	3B	84.9
Xfba213	3B	84.9

Xfbb156b	3B	84.9
Xfbb283	3B	84.9
XgbxG469	3B	84.9
Xgwm112	3B	84.9
Xgwm533	3B	84.9
Xpsp3112.1	3B	84.9
M77/P64.5	3B	85.9
Xfba214	3B	85.9
Xfba220	3B	85.9
Xfbb348	3B	85.9
Xopf3-960	3B	85.9
M67/P77.3	3B	86.9
Xcnl3d	3B	86.9
Xfbb177	3B	86.9
Xpsp3081b	3B	86.9
Xgwm131	3B	86.9
Tsn2	3B	87.9
XgbxG773	3B	87.9
Xbarc164	3B	87.9
wPt-1940	3B	88
wPt-7786	3B	88
Xwmc527	3B	88.2
TC74823b	3B	88.3
wPt-5769	3B	88.3
Xwmc418	3B	88.4
Xwmc334	3B	88.6
Xwmc827	3B	89
wPt-4599	3B	89.1
Xcfa2134	3B	89.3
Xbarc358	3B	89.4
wPt-6718	3B	89.5
Xbarc145	3B	89.6
Pm41	3B	90.4
Xwmc787	3B	90.5
Xcfd283	3B	94.2
Xgwm4b	3B	94.8
wPt-8886a	3B	94.9
Xcfd283a	3B	95.1
rPt-7068	3B	96.2
wPt-2280	3B	96.2
wPt-7688	3B	96.7
wPt-0280	3B	96.8
wPt-8141	3B	96.8
Xbarc229	3B	97.2
tPt-3599	3B	97.2

wPt-10270	3B	97.2
wPt-10437	3B	97.2
wPt-10442	3B	97.2
wPt-10600	3B	97.2
wPt-11304	3B	97.2
wPt-7145	3B	97.2
wPt-8964	3B	97.2
wPt-8412	3B	97.6
wPt-4719	3B	98.4
Xwmc291	3B	98.7
Xmwg818	3B	99.7
QTwt.crc-3B	3B	100.7
Xbarc344	3B	100.7
Br-B1	3B	101.7
M60/P64.13	3B	101.7
Xcdo583b	3B	101.7
Xfba242	3B	101.7
Xcdo718	3B	101.7
M77/P64.1	3B	103.7
M86/P65.4	3B	103.7
P78/M92.0	3B	103.7
P78/M92.6	3B	103.7
XksuG62	3B	103.7
P78/M92.5	3B	104.7
XksuF34	3B	104.7
Xmwg69	3B	104.7
Xfbb147	3B	105.7
P41/M48-4	3B	106.7
Xbcd141	3B	106.7
Xbcd147	3B	106.7
Xbcd1555	3B	106.7
Xfbb168	3B	106.7
Xgwm108	3B	106.7
wPt-0846	3B	107.2
wPt-10029	3B	107.2
wPt-6981	3B	107.2
wPt-9694	3B	107.4
wPt-1191	3B	107.6
TC87011	3B	108.4
wPt-4693	3B	109
wPt-10071	3B	109.5
wPt-0065	3B	109.7
Xcfa2170	3B	110.2
wPt-0384	3B	110.3
wPt-10785	3B	110.3

wPt-11301	3B	110.3
wPt-8480	3B	110.3
wPt-3856	3B	110.4
wPt-8238	3B	110.9
Xwmc54	3B	111.1
rPt-5523	3B	111.9
tPt-4541	3B	112.5
wPt-6961	3B	112.5
P36/M35-1	3B	112.9
P37/M53-4	3B	112.9
P39/M50-4	3B	112.9
XgbxG037b	3B	112.9
wPt-6785	3B	113.8
P34/M50-5	3B	114.3
P35/M37-7	3B	114.3
P35/M37-9	3B	114.3
Xbcd21	3B	114.3
XgbxG065	3B	114.3
XgbxG199	3B	114.3
Xbarc84	3B	114.3
wPt-1311	3B	114.5
wPt-10505	3B	114.9
wPt-5072	3B	114.9
wPt-5833	3B	114.9
wPt-7968	3B	114.9
wPt-8318	3B	114.9
P32/M36-1	3B	114.9
P32/M59-11	3B	114.9
Xfbb378	3B	114.9
wPt-8396	3B	115.4
wPt-6187	3B	115.8
P38/M42-4	3B	115.9
Xfbb274	3B	115.9
Xmwg11a	3B	115.9
wPt-10201	3B	116
wPt-3921	3B	116.1
Xbarc206	3B	117
P31/M50-3	3B	118.4
XWB084j	3B	118.4
Xfba133a	3B	118.4
wPt-10185	3B	118.5
wPt-3725	3B	118.6
wPt-2416	3B	118.7
P39/M50-3	3B	119.4
Xpsr1205	3B	120.4

Xbarc7	3B	121.4
Xabc174	3B	122.4
Xwmc687	3B	123.1
XWB077j	3B	123.5
P40/M60-3	3B	124.5
Xwmc326	3B	124.5
Xgwm4	3B	124.5
Xbarc77	3B	127.4
wPt-9826	3B	127.7
Xfba167a	3B	128
Xfba171	3B	128
wPt-8096	3B	129.1
Xfba360	3B	130
Xwia858.1(Glb35)	3B	130
P78/M69.7	3B	131
Xpsr386.4	3B	131
Xwmc206	3B	131.1
wPt-4412	3B	132.4
wPt-9158	3B	132.6
wPt-9714	3B	132.6
P32/M37-2	3B	132.9
P32/M37-4	3B	132.9
P32/M37-5	3B	132.9
XSsia	3B	132.9
Xbcd131	3B	132.9
wPt-9882	3B	133.8
Xbg131	3B	133.9
wPt-5704	3B	135.1
Xcdo105	3B	136
wPt-0668	3B	136.4
wPt-8276	3B	136.5
tPt-5389	3B	136.8
wPt-9892	3B	136.9
wPt-9989	3B	137.2
wPt-2172	3B	137.4
wPt-10680	3B	137.5
wPt-7416	3B	137.6
wPt-6725	3B	138
Xfba310	3B	138.1
Xfbb293	3B	138.1
Xtam63b	3B	139.1
wPt-11262	3B	139.5
wPt-10247	3B	139.6
wPt-10562	3B	139.6
wPt-10336	3B	139.7

wPt-10706	3B	139.7
wPt-10812	3B	139.7
wPt-11152	3B	139.7
wPt-3254	3B	139.7
wPt-11691	3B	139.9
wPt-10851	3B	140
wPt-10925	3B	140.5
wPt-4991	3B	140.9
wPt-1884	3B	141
Xmag695	3B	141.1
wPt-0021	3B	141.1
wPt-3342	3B	141.1
wPt-3480	3B	141.1
wPt-8959	3B	141.1
wPt-9786	3B	141.1
wPt-5295	3B	141.9
Xgwm299	3B	142
M17/P65.2	3B	144.1
Xgwm114	3B	144.1
Xpsr931	3B	145
wPt-8021	3B	148.5
Xwmc236	3B	151.9
Xfba217	3B	153.9
Xfbb316	3B	153.9
Xfba235	3B	155.9
Xpsp2151	3B	155.9
tPt-1093	3B	155.9
wPt-4194	3B	155.9
wPt-5947	3B	155.9
wPt-8559	3B	155.9
Xfba8	3B	156.9
Xfba167b	3B	157.9
tPt-7209b	3B	158.6
wPt-10000	3B	158.6
wPt-10025	3B	158.6
wPt-10240	3B	158.6
wPt-10428	3B	158.6
wPt-10555	3B	158.6
wPt-10829	3B	158.6
wPt-10890	3B	158.6
wPt-11082	3B	158.6
wPt-11088	3B	158.6
wPt-11330	3B	158.6
wPt-3153	3B	158.6
wPt-4517	3B	158.6

wPt-7802	3B	158.6
wPt-9185	3B	158.6
wPt-9766	3B	158.6
wPt-8845	3B	158.9
Xpsp3001.2	3B	158.9
wPt-8752	3B	159.4
wPt-5268	3B	160.1
Xgwm181	3B	160.9
Xgwm547	3B	161.9
wPt-6956	3B	162
wPt-5943b	3B	162.7
Xgwm751b	3B	163.4
wPt-2491	3B	164.9
Qsst.msub-3BL	3B	166.8
Xfba133b	3B	166.8
Xgwm247	3B	166.8
Xwmc261	3B	167.4
Xwmc274	3B	167.5
Xwmc632	3B	168.3
Xpsr604	3B	168.5
Xpsr454	3B	168.8
Xmwg11b	3B	170.2
wPt-10303	3B	172.7
wPt-11000	3B	172.7
wPt-11253	3B	172.7
Xgwm340	3B	173
wPt-4225	3B	173.4
wPt-10589	3B	173.8
wPt-0990	3B	174.7
wPt-6000	3B	175.4
R-D1	3B	178.1
wPt-1472	3B	180.9
wPt-7301	3B	184.2
D1.1_ctg787_1	3D	0
Xcdo549a	3D	0
Xcfd35	3D	0
XWC0035q	3D	2
Xwmc155b	3D	2
wPt-1336	3D	2.3
M65/P64.6	3D	3
M71/P77.4	3D	3
wPt-6358b	3D	3.1
Xgwm71	3D	3.3
Xwmc630	3D	3.3
wPt-9401	3D	5.8

wPt-1741	3D	6.1
wPt-1081b	3D	6.5
wPt-2757b	3D	6.7
Xcfd127	3D	7
Xcfd152	3D	7
Xcnl2a	3D	10.7
Xgwm161	3D	10.7
Xwmc674	3D	10.7
Xgwm183a	3D	14.7
Xcfd141	3D	19.7
Xbarc321.2	3D	22.7
Xgpw1106a	3D	22.7
XgpwE11b	3D	22.7
XWD173q	3D	24.7
Xcfd55	3D	25.7
D1.1_ctg1734_1	3D	28.7
Xbcd907a	3D	28.7
D1.1_ctg5743	3D	29.7
Xabc171x	3D	29.7
Xcfd79a	3D	29.7
Xfbb278.1	3D	29.7
XgbxG265	3D	29.7
Xwmc43	3D	30.3
Xwmc11	3D	30.8
Xfba190	3D	31.8
Xfbb370	3D	31.8
Xgwm161b	3D	31.8
D1.1_ctg4173	3D	33.8
Xbcd1802	3D	33.8
Xbarc128	3D	34
Xgpw322	3D	36.1
Xgwm2	3D	36.1
Xcfd70a	3D	40.1
Xcfd79	3D	45.1
Xgwm183	3D	47.6
Xgpw1007	3D	52.6
Xbarc8	3D	53.6
D1.1_ctg787_2	3D	54.6
Xcdo549b	3D	54.6
Xcfd64	3D	54.6
Xglk538c	3D	57.6
Xcfd34	3D	58.6
D1.1_ctg7512	3D	60.6
Xfba241	3D	60.6
D1.1_ctg12778-1	3D	61.6

D1.1_ctg4333_1	3D	61.6
XksuA6.2	3D	61.6
Xbcd1532	3D	64.6
Xcdo1435	3D	66.6
D1.1_ctg6151_1	3D	67.6
D1.1_ctg6228_1	3D	67.6
D1.1_ctg9045	3D	67.6
Xcdo407	3D	67.6
Xfba91a	3D	67.6
Xwmc529b	3D	68.6
D1.1_ctg6151_2	3D	69.6
D1.1_ctg6228_2	3D	69.6
Xfba91	3D	69.6
Xmwg22.2	3D	69.6
Xgwm2b	3D	70.6
Xgwm314	3D	70.6
Xgwm383	3D	72
Xgwm383b	3D	76.6
Xgwm664	3D	83.7
Xwmc505d	3D	83.9
D1.1_ctg12778_2	3D	84.9
D1.1_ctg4333_2	3D	84.9
XksuA6	3D	84.9
Xpsr394	3D	85.9
Xwmc443	3D	85.9
Xwmc375.1	3D	86.9
Xcfd2d	3D	87.9
Xgpw297b	3D	87.9
S-D1	3D	88.9
Xgwm114a	3D	90.7
Xbarc42	3D	90.9
Xbarc52	3D	91.1
Xwmc492	3D	92.5
Xcfd201	3D	94.4
Xgdm136	3D	96.4
Xgdm136c	3D	96.4
Xgdm99	3D	98.8
Xgwm707	3D	99.4
Xcfd223	3D	99.8
D1.1_ctg290	3D	100.6
D1.1_ctg4430	3D	100.6
Xcfd4a	3D	100.6
Xfba213	3D	100.6
Xfba214	3D	100.6
Xfbb324	3D	100.6

Xgpw1146b	3D	100.6
Xbarc68	3D	100.6
Xgwm497	3D	100.8
Xgwm52	3D	102.4
Xgwm456	3D	102.7
Xgpw1168	3D	103.3
Xgpw1251a	3D	103.3
Xgwm191	3D	103.3
Xgwm341	3D	103.3
Xgpw333	3D	104.3
Xopb8-550	3D	104.3
QGwt.crc-3D	3D	105.3
Xbarc6	3D	105.3
Xmwg688	3D	105.3
D1.1_ctg3640	3D	107.3
XWB042q	3D	107.3
Xbcd134	3D	107.3
Xcfd71	3D	107.4
Xwmc741	3D	107.4
Xwmc505	3D	108.9
Xcfd2	3D	109.1
Xcfd62	3D	109.4
Xcfd70	3D	109.6
Xabc176	3D	110
Xwmc533	3D	111.3
Xwmc656	3D	111.3
D1.1_ctg8418_1	3D	111.8
Xfbb23.1	3D	111.8
XgbxG305	3D	111.8
Xcfd193	3D	111.9
Xbarc125	3D	113.3
P78/M49.5	3D	113.4
Xcfd4	3D	113.8
Xcdo1406	3D	114.3
Xopab9-300	3D	116.3
M83/P65.9	3D	117.3
Xbcd288	3D	117.3
Xfba159	3D	118.3
Xgwm645	3D	118.3
Xwmc435	3D	118.6
Xcfd9	3D	120.2
Xwmc529	3D	120.3
Xwmc418	3D	123.9
Xcnl5b	3D	125
Xwmc631	3D	125.3

D1.1_ctg8418_2	3D	127.2
P78/M43.13	3D	127.2
Xfbb23.2	3D	127.2
Xfbb237a	3D	127.2
XgbxR187	3D	127.2
Xwia858.2(Glb35)	3D	128.2
D1.1_ctg1734_2	3D	130.2
D1.1_ctg6064	3D	130.2
D1.1_ctg8441	3D	130.2
P35/M50-3	3D	130.2
Xbcd907b	3D	130.2
Xcnl4.1	3D	130.2
Xpsr1203.2	3D	130.2
Xpsr170a	3D	130.2
D1.1_ctg5766	3D	131.2
P78/M59.3	3D	131.2
Xfba330	3D	131.2
D1.1_ctg10420	3D	132.2
D1.1_ctg620	3D	132.2
Xbcd22a	3D	132.2
Xgbx3864	3D	132.2
D1.1_ctg5319	3D	133.2
D1.1_ctg8783	3D	133.2
Xbcd1555a	3D	133.2
Xbcd372B	3D	133.2
Xgpw1251b	3D	133.2
D1.1_ctg1235	3D	134.2
Xbcd515	3D	134.2
D1.1_ctg13757	3D	135.2
Xfba027	3D	135.2
XksuD19	3D	135.2
Xcfd213a	3D	137.2
Xgwm3	3D	137.2
Xwmc549	3D	137.3
D1.1_ctg551	3D	139.8
D1.1_ctg9079	3D	139.8
XksuH15	3D	139.8
Xwg110	3D	139.8
Xcfd211	3D	141.8
Xgwm3b	3D	141.8
D1.1_ctg787_3	3D	142.8
Xcdo549c	3D	142.8
Xcfd219b	3D	143.8
Xgpw1062	3D	143.8
D1.1_ctg5041	3D	144.8

Xgpw1149	3D	144.8
XksuG59	3D	144.8
Xfbb117a	3D	145.8
Xwmc552	3D	146.8
Xfba310c	3D	147.8
Xbcd361	3D	148.8
H26	3D	149.8
Xabc174b	3D	149.8
Xgwm4b	3D	149.8
Xpsr904	3D	149.8
D1.1_ctg9019	3D	150.8
H32	3D	150.8
XksuE14a	3D	150.8
Xbarc270	3D	151.8
Xcnl2b	3D	151.8
D1.1_ctg3726	3D	152.8
D1.1_ctg5172	3D	152.8
D1.1_ctg5195	3D	152.8
Xfba27	3D	152.8
Xfba389	3D	152.8
Xfbb147e	3D	152.8
Xfbb269	3D	152.8
Xfbb316a	3D	152.8
Xbarc323	3D	155.8
Sr24	3D	156.8
Xabc172a	3D	156.8
Xcnl4.2	3D	156.8
Xfbb293	3D	156.8
Xgpw307	3D	156.8
Xmwg12	3D	157.8
Xfbb237b	3D	158.8
Xgwm114b	3D	158.8
Xcdo482	3D	160.8
Xmwg11b	3D	160.8
Xbcd451	3D	162.8
Xgdm72	3D	183.9
Xbarc71	3D	223.8
Xwmc179	4A	0
XS13M59(390)	4A	5.3
Xpsp3028	4A	6
P34/M50-5	4A	7
Xfbb1	4A	7
Xfba40a	4A	10
Xfba78a	4A	12
P40/M49-3	4A	13

Xmgb299	4A	13.7
Xgwm4	4A	15
Xpsr39	4A	16.6
Xwmc516	4A	19.2
P35/M57-2	4A	19.2
Xbcd1738a	4A	19.2
M21/P76.4	4A	20.2
Xmwg58	4A	20.2
M77/P64.6a	4A	21.2
Xbcd1652	4A	21.2
Xcdo959.1	4A	21.2
XksuF8a	4A	21.2
Ba2	4A	22.2
Xcdo1387	4A	22.2
Xgwm165	4A	23.2
Xpsr927.2	4A	23.2
Xrz574	4A	24.2
Xbarc206	4A	24.3
Xdupw238	4A	24.7
Xbarc138	4A	25
Xbarc106	4A	25.2
Xfbb332	4A	25.2
Xfba147	4A	27.2
Xwmc420	4A	27.3
Xfba320	4A	28.9
Xgwm30	4A	28.9
Xfba211a	4A	29.9
Xfba359	4A	29.9
Xwg464	4A	29.9
Xfba43	4A	31.9
Xgpw2283	4A	32.9
XgbxR247	4A	35.9
Xbcd8a	4A	36.9
Xgwm610a	4A	37.9
Xgpw2302	4A	38.9
Xfbb176	4A	39.9
Xfbb227	4A	39.9
Xwmc89	4A	40.9
Xwmc491	4A	41.2
Xwmc680	4A	41.2
Xwmc48	4A	41.4
Xcfd71	4A	42
Xgpw1010	4A	42
wPt-7524	4A	42
Xwmc173	4A	42.2

Xgwm601	4A	42.6
Xgwm48	4A	42.8
Xwmc15	4A	42.8
Xwmc446	4A	42.8
Xgwm44	4A	44.7
Xcfa2256	4A	44.8
Xbgl1485(Ger)	4A	44.9
XvutPDI	4A	44.9
Xwmc757	4A	45.1
Xmgb7	4A	45.7
Xwmc96	4A	48.2
Xwg876	4A	48.5
Xgwm610	4A	51.3
Xwmc617	4A	53.5
Xgwm192	4A	63.5
Xgwm464	4A	63.5
Xfba307	4A	65.2
Xbcd808	4A	67.1
Xgpw2140	4A	67.1
Xgpw2166	4A	67.1
Xabg484	4A	68.1
Xbcd1738	4A	68.1
XksuH9a	4A	69
Xfba40b	4A	70
Xfba78b	4A	71
Xpsr593.2	4A	73
Xgwm4b	4A	75
Xfba147b	4A	77
Xgpw2122	4A	77
Xfba211b	4A	78
Xmwg634.2	4A	78
Xwg622	4A	80
Xbcd402	4A	81
Xgwm397	4A	81
wPt-8489	4A	82.3
P41/M60-3	4A	82.5
XWD004da	4A	82.5
Xbcd8	4A	82.5
Xgpw2138	4A	82.5
Xbcd873	4A	84.5
Xfba65	4A	84.5
wPt-5455	4A	85.2
P32/M59-9	4A	85.5
Xgpw2279	4A	85.5
P35/M47-15	4A	86.5

P36/M49-9	4A	86.5
Xcdo948	4A	86.5
Xpsr1327	4A	87.3
P36/M47-3	4A	87.5
Xgwm269.2	4A	87.5
rPt-8568	4A	87.9
wPt-3638	4A	88
Phs1	4A	88.4
Xgwm622	4A	89.1
XksuG12	4A	89.4
P34/M58-2	4A	90.4
Xag12b	4A	90.4
Xfbb278	4A	90.4
CA499463	4A	90.7
Xdupw190	4A	90.8
Xglk752	4A	91.3
wPt-8479	4A	92
Xglk315	4A	92.3
Xgwm397b	4A	92.3
wPt-6303	4A	92.3
wPt-2788	4A	93.1
Xpsr59a	4A	93.3
Xpsr914	4A	94.3
Xwmc513	4A	94.3
Xcsl102(NBS-LRR)	4A	95.3
Xglk331	4A	96.3
Xgwm111	4A	96.3
Xmgb12	4A	98.5
Xglk600b	4A	99.5
XksuE3	4A	99.5
Xpsr934	4A	99.5
Xglk370	4A	100.5
Xgwm894	4A	101.4
Xgwm937	4A	102
Xpsp1316	4A	102.5
Xpsr1316	4A	102.7
Xdupw4	4A	103.4
Xwmc650	4A	104.4
Xbcd130a	4A	104.7
Xglk354	4A	104.7
Xgpw2244	4A	105.7
Xabg390	4A	107.7
Xcdo506	4A	107.7
wPt-2221	4A	108.2
Xgwm265	4A	111

Xbarc170	4A	112.8
Xutv1343b	4A	113.1
wPt-4660	4A	114.1
Xutv434	4A	115.4
wPt-1357	4A	115.8
Xwmc468	4A	120.5
Xwmc258	4A	121.7
Xgwm1081	4A	126
Xglk128	4A	126.1
Xgwm637	4A	126.4
Xmwg710a	4A	127.8
Xpsr115	4A	131
Xgwm118	4A	131.4
XS25M49(350)	4A	136.4
wPt-9169	4A	137.8
wPt-6515	4A	138
Xwmc707	4A	138.4
wPt-0162	4A	138.7
wPt-0817	4A	140.5
wPt-6330	4A	141.4
Xwmc161	4A	146.6
Xwmc161a	4A	147.7
Xgwm565	4A	149.5
Xgpw2228	4A	149.7
Xgpw2331	4A	149.7
Xmwg549.1	4A	149.7
Xutv1343a	4A	149.9
Xgwm494	4A	152.1
Xcfd257	4A	154.3
wPt-2247	4A	154.5
Xpsr580a	4A	159.3
XgbxG557	4A	160.1
wPt-2542	4A	160.7
wPt-3239	4A	161.1
wPt-5249	4A	161.4
wPt-7289	4A	161.4
wPt-1091	4A	162
wPt-6728	4A	162.9
wPt-5428	4A	163.7
wPt-5857	4A	163.8
wPt-3810	4A	164.5
Xwmc718	4A	165.1
Xbcd1670	4A	165.5
Xfba4a	4A	165.5
Xgwm162	4A	165.5

wPt-7939	4A	166
Xmwg710.1	4A	166.4
wPt-1262	4A	166.7
wPt-7558	4A	166.8
wPt-1550	4A	167.2
Xwmc760	4A	167.3
Xwmc597	4A	167.4
wPt-7926	4A	167.4
Xak466.2(Nra)	4A	167.5
Xcdo780a	4A	167.5
Xgwm834	4A	167.5
XgbxG036	4A	167.8
Xwg232.7	4A	168.4
wPt-3655	4A	169
Xpsr115.1	4A	169.3
P78/M59.6	4A	170.3
P36/M54-1	4A	171.3
Xpsr115.2	4A	171.3
Xcfd88	4A	172.3
Xgwm350.2	4A	172.3
wPt-6997	4A	173
wPt-1701	4A	173.1
Xgwm274	4A	173.3
XgbxG542	4A	173.7
Xwmc262	4A	174.1
wPt-2345	4A	176.2
wPt-9728	4A	176.2
Xwmc698	4A	177.5
XksuD9	4A	178
wPt-2129	4A	178.3
Xcdo475	4A	179.9
Xbarc343	4A	180.7
wPt-3766	4A	182.5
Xbcd130	4A	182.6
P35/M54-9	4A	183.1
P36/M53-338	4A	183.1
P42/M51-76	4A	183.1
Xfba231	4A	183.1
wPt-7280	4A	183.2
wPt-5749	4A	183.6
wPt-9833	4A	183.7
wPt-0105	4A	184
wPt-5825	4A	184
Xbcd135a	4A	185.2
Xpsr470(Wx)	4A	185.2

Xwmc2	4A	185.2
Xwmc283	4A	185.2
XgbxG035b	4A	186.2
wPt-5543	4A	187.4
wPt-4596	4A	188.7
P40/M60-221	4A	188.8
QMat.crc-4A	4A	188.8
Xfbb154	4A	188.8
P34/M48-2	4A	189.8
P40/M47-1	4A	189.8
P41/M61-4	4A	189.8
Xbcd135b	4A	189.8
Xfbb114a	4A	189.8
P34/M50-12	4A	190.8
XgbxR221	4A	190.8
Xgbx3480a	4A	191.8
M60/P64.10	4A	192.8
M67/P77.6	4A	192.8
XksuE3b	4A	192.8
P38/M47-7	4A	193.8
P41/M47-11	4A	193.8
wPt-1961	4A	194.7
wPt-8000	4A	194.7
P34/M51-90	4A	194.7
P36/M48-3	4A	194.7
Xabc465b	4A	194.7
Xcdo665C	4A	194.7
XgbxG141	4A	194.7
Xgpw356	4A	194.7
P31/M52-4	4A	195.7
P41/M47-12	4A	195.7
Xpsr490.2(Ss1)	4A	195.7
wPt-3061	4A	196
P31/M49-2	4A	196.7
P33/M49-8	4A	196.7
P78/M49.6	4A	196.7
TC85050	4A	197.1
M51/P65.6	4A	197.6
M60/P64.1	4A	197.6
P35/M61-5	4A	197.6
Xpsp3119	4A	197.6
P31/M57-1	4A	198.6
P39/M49-189	4A	198.6
P41/M51-157	4A	198.6
XWB078d	4A	198.6

Xwmc500	4A	198.6
Xcdo545	4A	198.7
Xgwm959	4A	199.6
wPt-7491a	4A	200.1
Xmag3886	4A	200.5
wPt-7168	4A	201
wPt-5003a	4A	201.1
wPt-3796	4A	201.3
wPt-2780	4A	201.5
Xmag974	4A	201.6
wPt-6440a	4A	201.7
wPt-2331	4A	201.9
wPt-9196	4A	201.9
Xwmc232	4A	202
wPt-8144	4A	202
wPt-9162	4A	202
wPt-1743	4A	202.1
wPt-2122	4A	202.1
Xgpw7056	4A	202.3
wPt-3150	4A	202.4
wPt-5735	4A	202.4
wPt-0538	4A	202.5
wPt-6502	4A	202.9
wPt-9183	4A	202.9
wPt-7821	4A	203.3
wPt-2909	4A	204.2
wPt-2006	4A	205.4
wPt-2301	4A	205.5
wPt-3108	4A	205.5
wPt-8167a	4A	205.5
wPt-8886b	4A	205.5
wPt-3349	4A	205.6
wPt-2084	4A	205.8
wPt-3795	4A	206.1
wPt-7807	4A	206.4
wPt-8886	4A	207.1
wPt-5003b	4A	207.5
wPt-6440b	4A	207.5
wPt-6404	4A	207.7
wPt-2794a	4A	207.8
wPt-1155a	4A	208
wPt-4241	4A	208.1
wPt-2291	4A	208.3
Xcfd30	4A	208.8
wPt-3449	4A	208.9

wPt-2983	4A	209.1
wPt-8167b	4A	209.1
wPt-2794b	4A	209.9
wPt-7354	4A	210.2
wPt-8271	4A	210.3
wPt-3095	4A	211.2
wPt-7104	4A	211.3
wPt-5099	4A	211.9
Xbarc70	4A	212.2
Xbarc78	4A	212.5
wPt-5172	4A	213
P42/M47-1	4A	214.9
Xcdo545.2	4A	214.9
wPt-9305	4A	215
P33/M49-6	4A	216.2
P35/M47-2	4A	216.2
P40/M55-6	4A	216.2
P40/M60-307	4A	216.2
XgpwE4	4A	216.2
Xpsr119b	4A	216.2
wPt-1362	4A	216.2
GBSS	4A	217.2
wPt-3506	4A	217.5
wPt-3491	4A	218.9
wPt-4487a	4A	219.4
P36/M47-1	4A	220.2
P41/M60-1	4A	220.2
Xtam72d	4A	220.2
Xgwm160	4A	220.2
wPt-4680	4A	220.2
wPt-0610	4A	221.4
wPt-5055	4A	221.5
wPt-0798	4A	221.8
wPt-1155b	4A	221.8
wPt-1520	4A	221.8
wPt-4424	4A	221.8
wPt-5112	4A	222
wPt-4620a	4A	222.4
wPt-0833a	4A	222.5
wPt-5434	4A	222.5
wPt-9150	4A	222.5
wPt-9954	4A	222.5
wPt-0992a	4A	222.6
wPt-3876	4A	222.7
Xmag1140	4A	222.8

Xmag3733	4A	222.8
wPt-3859	4A	222.9
wPt-2836	4A	223.2
wPt-1007	4A	223.3
wPt-3729	4A	223.3
wPt-2533	4A	223.4
F84	4A	223.7
Xwmc104	4A	224.1
wPt-0601	4A	224.3
Xcfd2	4A	224.4
wPt-9598a	4A	224.6
wPt-6757	4A	224.7
wPt-7405	4A	225
wPt-8091	4A	225
wPt-9103a	4A	225
wPt-0032	4A	225.3
wPt-2951	4A	225.3
wPt-3389	4A	225.3
wPt-5260	4A	225.3
wPt-0763	4A	225.8
wPt-6176	4A	225.8
wPt-7981	4A	225.8
rPt-0238	4A	226.3
wPt-0150	4A	226.3
wPt-5354	4A	226.3
wPt-6688	4A	226.3
Xwmc776	4A	226.5
wPt-6603	4A	227
wPt-9418	4A	227.2
wPt-8657	4A	227.5
P31/M58-1	4A	227.8
P32/M48-2	4A	227.8
P40/M61-7	4A	227.8
P78/M92.8	4A	227.8
Xbcd130b	4A	227.8
Xpsr160	4A	227.8
wPt-7919	4A	227.8
Xpsr392	4A	227.8
P32/M48-228	4A	228.8
P32/M48-4	4A	228.8
P32/M62-2	4A	228.8
P33/M55-1	4A	228.8
P35/M54-3	4A	228.8
Xabg378b	4A	228.8
Xabg704b	4A	228.8

Xpsr160a	4A	228.8
Xfbb194	4A	228.8
P31/M53-4	4A	229.8
P31/M60-3	4A	229.8
P33/M54-1	4A	229.8
P41/M47-8	4A	229.8
P33/M60-3	4A	230.8
Xfba282	4A	230.8
Xbcd1285	4A	232.8
Xpsr573	4A	232.8
wPt-9675	4A	234.5
P78/M68.y	4A	234.6
wPt-2151	4A	234.8
Xgbx3832b	4A	235.4
Xfbb114b	4A	236.4
P78/M68.x	4A	237.4
Xwmc313	4A	238.4
wPt-4064	4A	239.1
wPt-9059	4A	239.3
wPt-11389	4A	239.9
Xwmc722	4A	240.6
wPt-5489	4A	241.5
Stb12	4A	242.4
Stb7	4A	242.4
Xwmc219	4A	242.4
Xwmc497	4A	243.4
M65/P64.7	4A	244.4
Xpsr160.1	4A	244.4
XksuD9b	4A	245.4
wPt-1482a	4A	245.4
Xgwm350	4A	249.5
Xbarc327	4A	254.4
Xbcd1975	4A	254.4
wPt-9342	4A	258.4
Xpsr392.1	4A	260.4
Xfba243	4A	261.4
BQ169752	4A	263.3
QHt.inra-4A	4A	263.4
Xbarc52	4A	264.4
Xcdo780b	4A	268.4
Xbarc315	4A	272.4
Xbarc184	4A	278.4
Xbcd129	4A	283.4
Xbarc153	4A	297.4
Xbcd588	4A	303.4

Xfba253	4A	306.4
wPt-9824	4B	0
wPt-0246	4B	6
wPt-4699a	4B	6
wPt-9118	4B	6.3
wPt-8650a	4B	7.8
wPt-6869c	4B	9.3
wPt-1046	4B	9.7
wPt-5559	4B	9.7
wPt-1272	4B	11.9
wPt-1931	4B	40.9
P36M50b	4B	59.8
wPt-1708	4B	93.8
wPt-3908	4B	95.2
Xbarc10	4B	96
wPt-0608	4B	98.1
Xfba177a	4B	99.6
Xbarc68	4B	100.2
Xgwm664	4B	100.5
Xwmc679	4B	100.5
wPt-11203	4B	100.5
wPt-4537	4B	100.5
Xbcd402A	4B	100.6
Xblt801	4B	100.6
Xwmc652	4B	100.8
P34/M47-5	4B	102.6
Xfbb255a	4B	102.6
Xbarc193	4B	102.6
Xfbb67a	4B	103.6
Xwmc692	4B	103.8
tPt-5519	4B	103.8
wPt-9393	4B	103.8
TC80528b	4B	103.9
wPt-7365	4B	103.9
Xcfd39	4B	104.1
TC67416	4B	104.8
Xwmc617	4B	104.8
wPt-6149	4B	105.2
Xbcd446a	4B	105.6
Xgwm193	4B	105.6
wPt-3101	4B	106.1
Xfbb22a	4B	106.5
Xmwg2025a	4B	107.5
XgbxG260	4B	108.5
Xfba78a	4B	109.5

Xfbb24a	4B	109.5
Xgwm112	4B	109.5
Xwmc657	4B	109.5
Xgwm165	4B	109.5
XgbxG192c	4B	112.5
Xcdo795	4B	113.5
Xpsp3163	4B	113.5
Xbcd1250	4B	116.5
Xbarc292	4B	117.5
XgbxG057	4B	117.5
Xgwm925	4B	117.6
Xgwm608	4B	117.8
Xcdo1128	4B	118.3
Xgwm856	4B	118.3
Xgwm898	4B	118.3
Xbarc25	4B	118.4
Xwmc238	4B	118.4
Xbcd1262	4B	119
wPt-8892	4B	119.1
wPt-6123	4B	119.2
Xbcd2026	4B	119.8
Xbcd749	4B	119.8
Xcdo938	4B	119.8
Xfba8	4B	119.8
Xfbb121	4B	119.8
Xabg484	4B	120.8
Xmtd13	4B	120.8
Xwg110.2	4B	120.8
Xcsu25	4B	121.8
Xgpw1108b	4B	121.8
Xgpw1201a	4B	121.8
Xgpw2271b	4B	121.8
Xrz251	4B	121.8
Rht1	4B	121.8
Xrz672	4B	123.1
Xwmc826	4B	123.1
Xfba147	4B	124
Xcfa2091	4B	125
P31/M57-6	4B	126
P32/M53-3	4B	126
P32/M59-10	4B	126
P33/M54-4	4B	126
P40/M55-9	4B	126
P40/M60-126	4B	126
P44/M68-160	4B	126

Xbcd1051	4B	126
Xwmc48	4B	127
P38/M54-4	4B	127.1
Rht	4B	127.1
Rht-B1	4B	127.1
Xgwm1007	4B	127.1
Xgwm888	4B	127.1
Xwmc206.3	4B	127.1
Xcdo669b	4B	127.1
Xwmc491	4B	127.1
Xwmc511	4B	127.1
Xcfd2	4B	127.1
Xdupw23	4B	127.1
Xutv1386	4B	127.1
Xwmc206	4B	127.1
tPt-0602	4B	127.1
wPt-5497	4B	127.1
wPt-7392	4B	127.1
Xgwm495	4B	127.1
Xwmc89	4B	127.1
Xwmc254	4B	127.6
Xgwm107	4B	128.4
Xwmc419	4B	128.7
Xwmc695	4B	128.7
Xgwm113	4B	128.8
Xwmc16	4B	128.8
Xgwm66	4B	129.5
Xbcd1265	4B	130.5
Xbcd402B	4B	130.5
Xfba1	4B	130.5
Xfbb24b	4B	130.5
XE5M5m	4B	131.5
Xgwm495b	4B	131.5
CA694714	4B	131.7
Xgwm710	4B	131.7
Xgwm162.2	4B	132.4
QPhs.ocs-4B.1	4B	133.4
Xfba78b	4B	133.4
Xgwm368	4B	134.4
Xgwm540	4B	134.4
wPt-0872	4B	134.4
wPt-4931	4B	134.6
Xbarc20	4B	134.7
Xbcd110	4B	134.9
Xcfd283b	4B	135

Xmgb343	4B	135
Gai1	4B	135.2
Xpsr622	4B	135.3
XS25M49(365)	4B	135.5
XS13M59(450)	4B	135.7
CA663888	4B	135.9
XS13M60(360)	4B	136.4
Xwmc546	4B	136.4
BF484674-297	4B	137
Xgwm513	4B	137.5
Xfbb22b	4B	139
Xpsp3078	4B	139
Xgwm192	4B	139
M62/P64.4	4B	140
XksuC2	4B	140
Xwg282	4B	140
Xcfd283	4B	140.5
Xcdo1401	4B	141
XS13M61(170)	4B	141.2
Xgwm1084	4B	141.5
wPt-1505a	4B	141.5
Xgwm149	4B	141.7
Xcnl7.2	4B	142.7
Xfba41	4B	142.7
XksuG10	4B	142.7
Xpsp3030.2	4B	142.7
Xfbb178	4B	143.7
Xmwg2025b	4B	143.7
Xgwm375	4B	143.7
Xfbb58	4B	144.7
Xcdo1312	4B	145.7
Xgwm513b	4B	145.7
XksuE2	4B	145.7
Xcfd22	4B	146.7
wPt-7062	4B	147.1
Xgwm251	4B	147.7
wPt-6209	4B	147.8
wPt-4870	4B	148.1
Xwmc710	4B	148.3
wPt-7412	4B	148.4
Xwmc310	4B	148.5
Xbarc163	4B	149.8
Xcdo20.1	4B	150.3
Xfbb182	4B	151.3
Xgwm6a	4B	151.3

Xgwm930	4B	151.4
wPt-3991	4B	151.5
wPt-8107	4B	151.6
wPt-11493	4B	152.1
wPt-3255	4B	152.1
wPt-9223	4B	152.1
wPt-7858	4B	152.2
Xwmc349	4B	152.7
Xwg114.1	4B	152.8
Xwmc413	4B	153.7
Xbarc60	4B	154.8
Xgwm165b	4B	154.8
Xbarc109	4B	157.9
Xwmc47	4B	160.8
Xgwm538	4B	161.3
P40/M54-2	4B	164.3
P41/M51-299	4B	165.3
Xwmc125	4B	167.3
Xwb163k	4B	168.3
XWB060k	4B	170.3
Xfbb67b	4B	172.3
Xfbb255b	4B	175.3
wPt-0391	4B	179.3
Xgwm6b	4B	180.3
Xfba177b	4B	183.3
Xbcd402c	4B	188.3
wPt-5564	4B	189.8
wPt-8292	4B	191.3
wPt-3804	4B	191.5
wPt-5996	4B	191.5
Xpsr375.4	4B	194.3
Xdupw43	4B	198.7
M65/P64.8	4B	200.3
XWD043k	4B	204.3
Xwmc617	4D	0
D1.1_ctg11889_1	4D	10
D1.1_ctg5162_1	4D	10
D1.1_ctg5357_1	4D	10
D1.1_ctg5368_1	4D	10
Xbcd265a	4D	10
Xwmc285	4D	10
Xbcd1889	4D	11
Xfbb3	4D	13
Xgpw2046a	4D	13
Rht2	4D	13.8

Xgpw2066	4D	13.9
Xgpw2180	4D	17.9
D1.1_ctg1648	4D	19.9
D1.1_ctg2680	4D	19.9
Xbcd1930b	4D	19.9
D1.1_ctg5162_2	4D	21.9
D1.1_ctg5357_2	4D	21.9
D1.1_ctg5368_2	4D	21.9
D1.1_ctg7063	4D	21.9
Xbcd808B	4D	21.9
Xgwm888	4D	22.9
D1.1_ctg5649	4D	23.9
XksuE3c	4D	23.9
D1.1_ctg2905	4D	27.9
D1.1_ctg7196	4D	27.9
XksuH9	4D	27.9
Xwmc574	4D	29.2
Xwmc818	4D	29.3
D1.1_ctg2040	4D	29.9
D1.1_ctg6274	4D	29.9
Xmwg706	4D	29.9
Xmwg676b	4D	30.9
Xgwm497	4D	33.9
Xwmc89	4D	36.9
XksuH14	4D	37.4
Xwmc720	4D	37.4
Xwmc48	4D	38.5
Xcfd106	4D	40.4
Xcfd160	4D	40.4
Xgwm213	4D	40.4
Xgwm608	4D	41.2
Xwmc52	4D	41.4
Xbarc98	4D	42.3
Xcfd193	4D	42.5
Xbarc91	4D	42.8
Xwmc473	4D	43.4
Xgwm264	4D	46.4
XgbxG102	4D	51.4
Xgpw1209a	4D	51.4
Xmwg634	4D	51.4
Xcfd81b	4D	54.4
XgbxG081	4D	54.4
XgbxG129	4D	54.4
XgbxG152	4D	54.4
XgbxG192b	4D	54.4

XgbxG328	4D	54.4
Xgpw2271a	4D	54.4
Xgpw336	4D	54.4
Xgwm608a	4D	54.4
Xblt101.t7	4D	55.4
Xwmc419	4D	55.4
Rht-D1	4D	57.4
D1.1_ctg3738_1	4D	58.4
Xfbb251a	4D	58.4
Xcfd23	4D	58.4
Xgwm133	4D	58.6
Xcfd71	4D	58.7
Xwmc182	4D	59.5
Xwmc489	4D	59.9
Xgwm165a	4D	60.6
Xpsp3103	4D	60.6
Xwmc457	4D	60.6
Xcdo669	4D	62.6
Xbarc225	4D	69.6
Xfba211	4D	69.6
D1.1_ctg11889_2	4D	71.6
D1.1_ctg5162_3	4D	71.6
D1.1_ctg5357_3	4D	71.6
D1.1_ctg5368_3	4D	71.6
Xbcd265b	4D	71.6
Xbarc308	4D	72.6
Xwmc48b	4D	72.6
Alt2	4D	73.6
Xbarc217	4D	74.6
Xfbb13	4D	74.6
Xbarc288	4D	76.6
Xbcd327	4D	77.6
Xbarc334	4D	78.6
XksuF8.2	4D	79.6
D1.1_ctg3738_2	4D	80.6
Xfbb251b	4D	80.6
Xfbb336	4D	81.6
Xcfd89	4D	82.6
Xgwm192	4D	82.6
Xgwm193	4D	82.6
Xgpw342	4D	85.2
Xgwm165	4D	85.2
Xwmc33	4D	90
Xwmc331	4D	90
Xbcd1117	4D	91.4

Xwmc206	4D	92.4
Xwmc399	4D	92.5
D1.1_ctg4222	4D	94.5
D1.1_ctg6238	4D	94.5
D1.1_ctg6392	4D	94.5
Xbcd15.1	4D	94.5
Xcfd39b	4D	96.1
Xfbb178a	4D	96.5
Xcfd39	4D	97.5
Xgpw311a	4D	99.5
XgpwE2	4D	100.5
AltBH	4D	102.5
D1.1_ctg9337_1	4D	102.5
Xcdo1312a	4D	102.5
D1.1_ctg6064_1	4D	106.5
D1.1_ctg8441_1	4D	106.5
Xbcd1431.1	4D	106.5
Xcfd84	4D	106.5
Xcdo1081	4D	107.5
Xwmc622	4D	109
D1.1_ctg3701	4D	111.5
D1.1_ctg4161	4D	111.5
Xmwg710a	4D	111.5
Xbarc200	4D	113.2
Xcfd54a	4D	113.5
Xbarc48.4	4D	114.5
D1.1_ctg10323	4D	116.5
D1.1_ctg10469	4D	116.5
D1.1_ctg18	4D	116.5
D1.1_ctg8730	4D	116.5
D1.1_ctg9093	4D	116.5
Xfbb322b	4D	116.5
D1.1_ctg1187	4D	120.5
D1.1_ctg5687	4D	120.5
D1.1_ctg8300	4D	120.5
Xfbb226e	4D	120.5
Xgwm194	4D	120.5
Xfba177b	4D	123.5
Xgpw345	4D	123.5
D1.1_ctg6064_2	4D	126.5
D1.1_ctg8441_2	4D	126.5
Xbcd1431.2	4D	126.5
XksuS11	4D	126.5
Xwmc74	4D	127.1
D1.1_ctg6064_3	4D	127.5

D1.1_ctg8441_3	4D	127.5
Xbcd1431d	4D	127.5
Xcdo949	4D	127.5
Xwmc74b	4D	127.5
X_beta-Amy-1	4D	128.5
Xwmc825	4D	129.3
Xpsr375.1	4D	131.4
Xgwm624	4D	133.4
Xgwm609	4D	135.8
XgbxG623	4D	138.8
Xcdo1528b	4D	141.8
Xcdo394b	4D	141.8
Xgwm538	4D	144.8
Xwg114b	4D	147.8
Xfbb182	4D	149.8
Xgpw2328b	4D	150.8
Xgpw311b	4D	150.8
Xgwm149	4D	152.8
Xgwm251	4D	155.8
XgbxG276	4D	157.8
Xcdo20b	4D	159.8
Xgwm6	4D	161.8
Xfbb58a	4D	163.8
Xgbx3581b	4D	165.8
D1.1_ctg9337_2	4D	168.8
Xcdo1312b	4D	168.8
XksuE2	4D	168.8
XksuG10	4D	169.8
Xcdo1401	4D	170.8
Xfba41	4D	170.8
Xfbb178b	4D	170.8
Xcfa2173	4D	173.4
Xbarc10	5A	0
Xpsr644	5A	21.7
Xwmc47	5A	26.1
P32/M62-7	5A	30.3
Xcnl6b	5A	30.3
Xpsr170c	5A	30.3
Xwmc713	5A	30.3
Xbcd873a	5A	31.3
Xpsr945	5A	32.1
Xfbb276b	5A	32.3
Xfbb277c	5A	32.3
Xpsr549	5A	32.3
Xbarc122	5A	33.3

Xbarc135	5A	33.3
XgbxR665	5A	34.3
Xglk163a	5A	34.3
Xbarc316	5A	37.3
Xwmc169	5A	37.3
Xgwm205a	5A	37.3
Xgdm109	5A	38.6
Xgwm154a	5A	39.4
Xwmc654	5A	40.3
Xcfa2104	5A	42.2
wPt-1165	5A	44.8
Xwmc51	5A	49.6
BF202040-164	5A	51.8
wPt-2768	5A	52.2
wPt-4131	5A	52.4
wPt-9887	5A	54.4
XS13M60(570)	5A	65.3
Xutv711	5A	69.5
Xgwm443a	5A	71.4
XgbxG625	5A	72.4
Xmag694	5A	75.7
Xwmc489	5A	76
P41/M61-2	5A	77.6
Xcfa2190	5A	77.6
Xpsr644a	5A	78.6
XS13M50(295)	5A	79.9
P78/M92.1	5A	81.3
Xbcd1871.1	5A	81.3
wPt-0605	5A	81.6
Xpsr120	5A	82.5
Xmgb341	5A	83
Xabg397	5A	83.2
Xbarc303	5A	83.2
Xcdo785	5A	83.4
Xcfd17b	5A	84.3
Xgpw358b	5A	84.3
Xwg232.3	5A	84.3
Xubp3	5A	84.5
XWB117e	5A	85.1
Xpsr326.1	5A	85.1
Xpsr386.3	5A	85.1
wPt-3924	5A	85.9
XWB180e	5A	86.1
Xfba352	5A	86.1
Xpsr945a	5A	86.1

TC91851	5A	86.3
Xcdo749.1	5A	87
Xfba131	5A	87
P34/M47-2	5A	88
P78/M43.10	5A	88
Xmgb340	5A	88.5
Xbcd1355	5A	88.8
Xpsr911a	5A	89.6
Xbarc358	5A	90.3
Xgwm328	5A	90.4
P36/M50-1	5A	91.8
Xpsr574	5A	91.8
F118	5A	92.3
Xbarc1	5A	92.3
Xgwm293	5A	92.4
Xbarc197	5A	93.5
Xcdo1335	5A	95
wPt-3884	5A	95
Xcdo341a	5A	95
Xgwm415	5A	95
Xwmc752	5A	95
Xgwm129	5A	95.1
P78/M43.3	5A	96.1
P78/M92.7	5A	96.1
XWB141e	5A	96.1
Xglk424	5A	96.1
Qfhs.ifa-5A	5A	97.1
Xpsr150	5A	97.1
Xwmc2.194	5A	97.1
Xcfa2250	5A	97.1
Xwmc150	5A	97.6
Xwmc705	5A	98.5
Xbarc117	5A	98.7
wPt-9452	5A	98.7
Xmgb191	5A	98.8
Xbarc186	5A	99.2
wPt-3620	5A	99.4
Xwmc2.169b	5A	100.3
Xwmc2.54	5A	100.3
Xbarc56	5A	100.9
Xgwm786	5A	100.9
Xbcd1335b	5A	102.4
Xpsb85	5A	102.4
Xwg564a	5A	102.4
Xwmc805	5A	102.4

Xmgb63	5A	102.7
Xbcd21	5A	102.9
Xmag3794	5A	103.2
Aadh-A1	5A	103.3
Xbarc180	5A	104.1
Xgwm304	5A	104.3
P41/M49-76	5A	104.4
BJ262177a	5A	104.9
Xbarc141	5A	105.4
XksuG44	5A	106.3
Xgwm156a	5A	106.6
Xbarc360	5A	108
Xmag4060	5A	108.6
wPt-3509	5A	109
wPt-2543	5A	109.4
wPt-6825	5A	109.5
Xgwm186	5A	109.7
wPt-4248	5A	109.8
XksuH1	5A	110
wPt-8226	5A	110.1
Xcdo57	5A	110.4
Xmag1159	5A	110.4
Xcdo412	5A	111.2
M85/P65.2	5A	111.9
P41/M47-10	5A	111.9
Xbarc165	5A	111.9
Xwmc446	5A	111.9
Xcnl3b	5A	112.9
Xwg184	5A	113.9
P41/M48-184	5A	115.9
Xglk612	5A	115.9
Xgwm205	5A	115.9
Xpsr912	5A	115.9
Fr-A2	5A	116.9
Xgwm154	5A	116.9
Xgpw2311	5A	117.9
Xglk317c	5A	118.9
XksuD16	5A	118.9
Xglk614a	5A	119.9
Xbcd926	5A	120.9
Xmwg624	5A	120.9
Xpsr128	5A	120.9
Xbcd157b	5A	121.9
M71/P77.8	5A	122.9
Xcdo666	5A	122.9

Xfbb2	5A	122.9
Xcdo1090A	5A	123.9
Xpsr911	5A	124.9
Xbcd1088	5A	125.9
Xbcd981	5A	125.9
Xpsr1202	5A	125.9
Xpsr806.1	5A	125.9
Xbcd1874a	5A	126.9
Xbcd1949	5A	126.9
Xcdo57B	5A	126.9
Xmwg522	5A	126.9
Xcfa2121	5A	128.1
wPt-5588	5A	129.3
Xbarc100	5A	129.6
Xwmc795	5A	130.1
Xgwm564	5A	130.3
Xgwm96	5A	134.3
Xwmc492	5A	135
wPt-7185	5A	137.1
Xbcd1235	5A	137.4
Xbarc330	5A	137.5
Xbcd508	5A	138.7
Xbarc40	5A	143.4
P78/M43.9a	5A	146.7
Xbcd1235.1	5A	146.7
Xgwm156b	5A	146.7
P31/M55-3	5A	147.7
QEet.fcu.5AL	5A	147.7
Xpsr637	5A	147.7
Xbcd1235a	5A	148.7
tPt-0353b	5A	149.1
wPt-7201	5A	149.1
Xmwg914a	5A	149.6
Xpsr120a	5A	149.6
Xgwm617	5A	150.2
P33/M60-5	5A	150.6
Xpsr120b	5A	150.6
Xfba166a	5A	151.6
Xpsp3003	5A	151.6
Xfbb255x	5A	152.6
P35/M54-6	5A	153.6
Xpsr967.3	5A	153.6
Xbcd183	5A	153.6
P32/M51-7	5A	154.6
P38/M54-8	5A	155.6

Xgwm121.2	5A	155.6
Xwmc415	5A	156.6
Xcfd2	5A	157.7
Xcfd2g	5A	158.3
Xwmc630f	5A	158.7
Xwmc630	5A	159.5
Xwmc475	5A	160.7
Xbarc151	5A	167
F128	5A	167.4
Xwmc388	5A	167.6
Xgwm666	5A	168
Xgwm443	5A	168.9
Xwmc96	5A	171.4
Ksum137	5A	172.1
Xcfa2163	5A	177
Xcfa2155	5A	180.2
Xcfa2141a	5A	181.3
P40/M61-9	5A	182.7
Xpsr2021.1(Aba2)	5A	182.7
Xmwg514	5A	182.7
Xfba190.1	5A	183.7
Xpsr575.2	5A	183.7
P33/M52-4	5A	184.7
XgbxG722b	5A	184.7
Xwmc445	5A	185
Xwmc96b	5A	185.2
Xgwm639	5A	185.7
Xrz395.1	5A	185.7
Xpsr426	5A	185.8
Xbarc232a	5A	186.2
Xbarc232	5A	187.5
Xcfa2185	5A	190.3
P41/M49-338	5A	192.1
Vrn1	5A	192.1
Xwmc327	5A	192.1
Xbarc230	5A	193.1
Xcdo1326	5A	195.1
Xfba68	5A	196.1
P31/M58-2	5A	197.1
Xpsr426.1	5A	197.1
Xbarc319	5A	198.1
Xfbb209.1	5A	199.1
Xgpw1086	5A	199.1
P40/M54-4	5A	200.1
Xfbb199	5A	200.1

Xgpw2059	5A	201.1
M85/P65.0	5A	203.1
Xgpw2328a	5A	203.1
Xrab16	5A	203.1
Xfba351a	5A	204.1
wPt-9834	5A	204.4
wPt-7255	5A	205.5
Xwmc216.3eb	5A	205.8
P38/M59-147	5A	207.8
Xbcd1235b	5A	207.8
Xgpw2120	5A	209.8
Xgpw2136	5A	209.8
Xgpw2181a	5A	209.8
M60/P64.4	5A	211.8
Xabg391	5A	211.8
Xgpw2172	5A	211.8
Xcdo457	5A	212.8
P35/M55-4	5A	213.8
Xbcd1235.2	5A	213.8
Xgpw2273a	5A	213.8
Xpsr145	5A	213.8
P33/M60-1	5A	215.8
Xabg366	5A	215.8
Xpsr1194	5A	215.8
Xwmc110	5A	218.1
Xpsr918	5A	223.7
wPt-9800a	5A	226.9
Xcdo1312c	5A	227.4
Xfbb249	5A	228.4
Xgwm126	5A	228.5
Xfbb330	5A	229.5
Xcfd39	5A	230.5
Xcfa2149	5A	231
Xpsr918b	5A	231.3
Pm2026	5A	232.4
Xwmc577	5A	232.5
Xcdo20a	5A	232.6
Xcdo1528a	5A	233.6
Xcdo394a	5A	233.6
Xwg114a	5A	233.6
Xgwm179	5A	233.6
Xgwm114	5A	235.4
Xwmc2.180	5A	235.5
Xgwm595	5A	238.5
Xpsr1201b	5A	238.5

Xwmc524	5A	240.5
wPt-5231	5A	241.9
Xwmc727	5A	244.3
Xgwm6.2	5A	252.1
Xgwm6b	5A	252.1
Xmwg2112	5A	254.1
Nax2	5A	255.1
Xgwm291	5A	255.1
Xgwm410	5A	257.8
B1	5A	259.6
M65/P64.9a	5A	261.6
Xgwm410.1	5A	261.6
P43/M62-5	5A	262.6
beta-Amy-1	5A	268.6
Xcfd5	5B	0
BE404594-175	5B	3.2
Xwmc773	5B	8.2
Xwmc630	5B	12.5
Xbarc240	5B	13.1
Xbarc21	5B	14.2
Xcfd60	5B	15.4
Xwmc47	5B	25
wPt-3439c	5B	29.6
Xwmc728	5B	43.4
Xcfd20	5B	44.4
Xgwm234	5B	44.5
wPt-6136	5B	45.4
wPt-1302	5B	45.7
wPt-9800b	5B	50.7
wPt-0033	5B	54
Xdupw115	5B	54.8
Lr52	5B	56.5
P39/M50-4	5B	56.5
Xbcd873a	5B	56.5
Xfbb276a	5B	57.5
Xgwm443	5B	57.7
Xpsr170a	5B	58.5
P32/M47-1	5B	62.5
P31/M49-3	5B	64.5
Xgpw1056	5B	64.5
P35/M52-3	5B	65.5
wPt-0819	5B	66.4
P31/M57-3	5B	66.5
P32/M48-234	5B	66.5
P42/M60-4	5B	66.5

Xcdo749a	5B	66.5
Xpsr326a	5B	66.5
Xpsr929a	5B	66.5
wPt-7861	5B	67.6
P38/M59-114	5B	68.7
Xgwm443.2	5B	68.7
wPt-1420	5B	69
wPt-9666	5B	69
wPt-8604	5B	69.1
wPt-9724	5B	69.9
Xfba232a	5B	70.7
wPt-1261	5B	71.3
Xfbb238	5B	72.8
Xgpw1072	5B	72.8
P78/M72.3	5B	73.8
Xcdo1335	5B	73.8
Xfba393a	5B	73.8
Xfba342a	5B	74.8
Xwmc149	5B	75.8
Pm16	5B	77.8
Xcfa2121a	5B	78.5
Xgdm146	5B	80.8
Xwmc274	5B	81.6
wPt-3931	5B	81.6
Xwmc813	5B	81.6
Xwmc740	5B	82.2
Xgwm159a	5B	83
Xfbb161	5B	83.1
Xfbb277.1	5B	83.1
Xgwm133	5B	83.1
P33/M52-9	5B	86.1
Xfbb292	5B	86.1
Xgwm66a	5B	87.1
Xbcd873b	5B	87.4
Xgwm540	5B	87.4
Xwmc247	5B	88.4
Xgwm191	5B	89
Xcdo959.2	5B	90.5
Xgwm843.1	5B	90.5
P36/M48-2	5B	91.5
XWD115l	5B	91.5
wPt-5346	5B	92.4
wPt-5914	5B	92.4
wPt-6348	5B	92.4
Xgsy60	5B	92.5

Xwmc333.2	5B	92.5
wPt-5175	5B	92.5
P39/M53-2	5B	93.7
Xopf8-170	5B	93.7
Xwmc376	5B	93.7
Xwmc682	5B	93.7
Xwmc386	5B	93.8
Xpsr326	5B	96.9
Xpsr929	5B	96.9
Xbarc4	5B	97.2
CA707573	5B	98.3
Xbarc340	5B	99.5
wPt-5688	5B	99.5
wPt-9814a	5B	99.6
wPt-5737a	5B	99.8
wPt-6052	5B	99.8
wPt-2041	5B	100
tPt-0228	5B	100.2
Xmwg561	5B	101.7
wPt-2586	5B	101.8
Xgwm544	5B	102.1
wPt-6263	5B	103.5
Xwmc616	5B	103.8
Xwmc73	5B	104.2
Xcfa2070	5B	104.8
Xwmc363	5B	105.3
Xfbb121.2	5B	106.3
Xfba367.1	5B	107.3
Xwmc188	5B	107.3
Xbarc32	5B	108.3
P39/M49-82	5B	110.3
P42/M51-116	5B	110.3
Xgwm293	5B	110.3
Xmtd116	5B	111.3
Xbarc88	5B	111.4
Xgwm274	5B	111.5
Xgwm68a	5B	112.2
Xbarc216	5B	112.4
Xgwm67	5B	112.5
Xwg232.2	5B	113.3
Xpsr170b	5B	114.3
Xpsr304	5B	114.3
M71/P77.13	5B	115.3
P38/M61-3	5B	115.3
Xcfd219a	5B	115.3

Xfba393b	5B	115.3
XgbxG060	5B	115.3
Xabg705	5B	116.3
Xcdo1338x	5B	117.3
Xfba342b	5B	117.3
Xbcd1871.2	5B	118.3
Xcdo749b	5B	119.3
XgbxG198	5B	119.3
Xgwm66b	5B	119.3
Xpsr929b	5B	119.3
Xbcd1024	5B	120.3
Xfba232b	5B	121.3
Xgwm159	5B	121.3
Xbarc109	5B	122.3
Xgwm68b	5B	122.3
wPt-1457a	5B	122.5
wPt-1505b	5B	122.6
Xgwm247	5B	122.8
M51/P65.4	5B	122.9
Xbarc89	5B	123.1
wPt-3569b	5B	123.5
wPt-1951	5B	123.8
Xbarc176	5B	123.9
wPt-8106a	5B	124
wPt-6902	5B	124.1
Xgpw1102	5B	124.3
Xpsr118	5B	124.3
Xpsr326b	5B	124.3
Xgwm564	5B	124.5
Xpsr128	5B	124.5
wPt-9467a	5B	124.5
wPt-5514	5B	124.7
Ne1	5B	125.3
Xmwg561.1	5B	125.3
wPt-0103a	5B	125.3
XgbxG541	5B	125.6
Xpsr128.1	5B	125.6
Xpsr574	5B	125.6
Xgwm335	5B	125.6
Xgwm213	5B	125.9
wPt-8890	5B	126
Xwmc435	5B	127.2
Stb1	5B	127.9
Xabc164	5B	127.9
Xwg564b	5B	127.9

Xwg889	5B	127.9
Xbarc74	5B	127.9
P78/M69	5B	128.9
XWB109l	5B	128.9
Xbcd157.1	5B	128.9
Xgpw358a	5B	128.9
Xwg909	5B	128.9
Xwmc745	5B	129.4
Xfba127c	5B	129.9
Xgwm831	5B	129.9
Xmta6	5B	129.9
Xgwm777	5B	130.2
wPt-4996	5B	130.4
wPt-0935	5B	130.5
QGpc.ndsu-5B.3	5B	131
XksuA1	5B	131
Xgwm371	5B	131.5
P78/M49.3	5B	132
Xcdo412	5B	132
Xfba65	5B	132
Xtam72c	5B	132
Xcfd2h	5B	132.7
XWB074l	5B	133.1
Xcfd2a	5B	133.1
Xcfd2	5B	133.2
Xgwm499	5B	134
P40/M55	5B	134.2
wPt-4936	5B	135
P37/M49-4	5B	135.2
Xgwm639	5B	136
wPt-7167	5B	136.1
Xbcd1140	5B	136.5
Xwg530	5B	136.5
Xbcd351	5B	136.5
Xgwm1073	5B	137
Xwmc405	5B	137.1
P31M48i	5B	137.6
Xwmc759	5B	138.2
wPt-4246	5B	138.5
wPt-2318	5B	138.7
Xdupw395a	5B	138.8
P37/M47-3	5B	139.3
Xfbb121a	5B	139.3
Xgpw1082	5B	139.3
Xwmc415	5B	139.3

wPt-3661	5B	139.3
wPt-3457	5B	139.8
wPt-7101	5B	139.9
P37/M51-3	5B	140.1
P40/M55-4	5B	140.1
P40/M61-11	5B	140.1
Xpsr725	5B	140.1
Xpsr911	5B	140.1
wPt-4628	5B	140.1
Xgwm530	5B	140.2
wPt-5851	5B	140.2
wPt-9356	5B	140.2
Ml3D232	5B	140.6
wPt-1250a	5B	140.6
wPt-3503a	5B	140.6
Xbcd508.1	5B	141
Xcdo1192	5B	141
wPt-9613	5B	141.9
P35/M55-2	5B	142
Xabg473.1	5B	142
Xmwg52	5B	143
Xwmc537	5B	143.4
P32/M51-4	5B	144
P35/M52-2	5B	144
XgbxG739	5B	144
P41/M60-6	5B	145
Xcdo1168	5B	145
Xgwm554b	5B	145
Xcdo400	5B	145
Xpsr911b	5B	145
P44/M68-109	5B	146
P37/M54-1	5B	147
P33/M60-4	5B	148
Xmwg922	5B	148
wPt-6135	5B	148.6
Fr-B2	5B	149.8
P31/M56-3	5B	150.8
Xmwg914	5B	150.8
P32/M59-8	5B	152.8
P34/M58-3	5B	152.8
Xcdo346A	5B	152.8
Xpsp3037	5B	152.8
Xbcd307.2	5B	153.8
Xgwm554	5B	153.8
BQ237037-30	5B	154

Xcfd7	5B	159.4
Xgpw7356	5B	160
wPt-0498	5B	160
P38/M51-1	5B	160.9
PmAS846	5B	161.4
Xcfd7a	5B	161.6
Xbcd9	5B	162
Xpsr806.2	5B	162
wPt-9454	5B	162.7
Xfba166.1	5B	163.1
Xfba348	5B	163.1
Xopal7-470	5B	163.1
Xwg583a	5B	164.1
Xfba332.1	5B	166.1
Xfbb237.1	5B	166.1
wPt-5928	5B	166.8
wPt-5604	5B	166.9
wPt-8054	5B	166.9
Xpsr120.2	5B	167.2
Pm36	5B	167.6
BJ261635	5B	168.1
wPt-1733	5B	168.1
wPt-2453	5B	168.1
wPt-4367	5B	168.1
wPt-6989	5B	168.1
wPt-9300	5B	168.1
Xgwm271b	5B	170
Xwmc289a	5B	170
Xgwm271a	5B	170.2
Xcfd156	5B	170.4
Xfba237	5B	170.4
Xwmc326	5B	171
wPt-6014	5B	171.8
Xwmc75	5B	172.3
Xfba259c	5B	173.3
M65/P64.8a	5B	174.3
Xfba332.2	5B	174.3
Xbcd1030	5B	176.3
P41/M47-5	5B	179.3
XgbxG722a	5B	179.3
Xcdo504	5B	180.3
Xpsr2021.2(Aba2)	5B	180.3
P42/M60-1	5B	181.3
XgbxG723	5B	181.3
Xglk510b	5B	181.3

Xcdo465b	5B	182.3
P32/M59-4	5B	183.3
Xbcd450B	5B	185.3
PA41/M47-4	5B	186.3
Vrn-B1	5B	187.3
P43/M59-1	5B	190.3
QYld.ndsu-5B	5B	190.3
Xgwm604	5B	190.3
Xcdo348	5B	191.3
Xfbb328	5B	191.3
Xgwm408	5B	191.3
Xwmc810	5B	195.6
QGpc.ndsu-5B.1	5B	196.5
QGw1.inra-5B	5B	196.5
Xcdo1326.1	5B	196.5
Xpsr426.2	5B	197.5
Xwmc500g	5B	197.5
rPt-7889	5B	199
Xwmc500	5B	200.8
Xbarc140	5B	201.9
Xcdo584	5B	202.9
tPt-1253	5B	203.3
wPt-8931	5B	203.8
Xfba351.2	5B	203.9
Xbarc156	5B	204.9
Xpsr157	5B	204.9
Xwmc99	5B	204.9
Xcfa2121b	5B	206
Xbarc308.1	5B	206.1
Xwg908a	5B	206.1
wPt-2707	5B	206.3
wPt-1482b	5B	206.7
wPt-3076	5B	206.7
wPt-9103b	5B	206.7
wPt-9598b	5B	206.7
Xbarc142	5B	207.4
Xbcd221	5B	207.4
XgbxG521	5B	207.4
wPt-8094	5B	207.4
Xbarc25b	5B	208.1
Xwmc734	5B	209.1
Xgpw2243a	5B	209.4
Xgdm116	5B	210
Xwmc160	5B	210.3
P40/M55-7	5B	211.1

XgbxR570	5B	211.1
Xbarc232	5B	211.1
wPt-4577	5B	213.7
Xcfd86b	5B	216.8
P34/M47-4	5B	218.8
P34/M50-1	5B	220.8
XSsib	5B	220.8
Xwmc235	5B	220.8
Xcfd86	5B	221.3
Xwmc28	5B	221.5
P39/M49-135	5B	222.5
P32/M52-243	5B	223.5
Xfbb323	5B	223.5
Xwmc27	5B	223.5
Xwmc118	5B	224.5
Xwmc508	5B	225.5
Xgwm790	5B	226.1
wPt-8418a	5B	233.8
wPt-7561	5B	234.4
Xwmc640	5B	235.8
P32/M52-374	5B	237.6
Xbarc69	5B	237.6
wPt-1348	5B	241
wPt-1179	5B	241.7
Xbarc59	5B	243
Xwmc430	5B	243.1
Xwmc783	5B	246.4
Xpsp3065	5B	247.9
Xwg583b	5B	248.9
Xopaf5-650	5B	252.9
Xgwm497	5B	254.7
wPt-7665	5B	259.4
wPt-9116	5B	262.1
Xwmc258	5B	263.3
Xgwm118	5B	263.9
wPt-0054	5B	264.1
wPt-5429	5B	264.9
wPt-1500	5B	265.6
wPt-8920a	5B	265.9
wPt-0295	5B	266
wPt-4402	5B	266
wPt-7708	5B	266.9
wPt-4723	5B	267.6
wPt-3922	5B	267.7
wPt-5373	5B	268.9

wPt-5168	5B	269.7
Xabc310.1	5B	271.9
TC86533	5B	272
QGpc.ndsu-5B.2	5B	273.9
wPt-0484	5B	273.9
Xwg908b	5B	276.9
wPt-7036	5B	279.4
wPt-8553	5B	281.2
wPt-0837	5B	290.6
Xpsr580b	5B	307.1
Xpsr143	5B	325.8
Xwmc233	5D	0
wPt-1400	5D	0.1
Ha	5D	3.4
Pina-D1	5D	3.4
Xcfd165	5D	3.4
Xmta10	5D	3.4
Xmta9	5D	3.4
Xbarc130	5D	3.4
Xbarc140	5D	3.4
Xcfd18	5D	4
Xgwm190	5D	8.5
Xgpw326	5D	9.5
Xgwm192	5D	11.5
Xfba114	5D	12.5
wPt-2856	5D	16
Xcfa2104	5D	20
Xfba393b	5D	20
Xcfd189	5D	20.9
wPt-5766	5D	23.2
Xbarc205	5D	23.4
Xgwm205	5D	25.1
Xfbb238	5D	25.8
PmD57-5D	5D	32.6
Xcfd78	5D	36.4
Xfba137	5D	37.4
XgbxR958	5D	38.4
Xgdm68	5D	39.4
Xbarc44	5D	41.4
XksuD30	5D	41.4
Xopai5-540	5D	41.4
Xwmc150	5D	41.4
Xwmc606a	5D	41.6
Xbarc143	5D	41.9
Xcfd37	5D	43.2

Xcfd67	5D	43.4
Xgwm358	5D	43.9
Xgwm16	5D	44.3
Xgwm159	5D	45
Xwmc608	5D	45.1
Xwmc318	5D	45.3
Xcfd81	5D	46.1
Xcfd74	5D	46.3
Xwmc805	5D	47
P41/M51-309	5D	47.2
Xgpw302	5D	47.2
Xpsr326b	5D	48.2
Xgdm153	5D	49
Xcmwg770	5D	49.1
Xwmc799	5D	49.6
Xcfd266	5D	50
P39/M49-339	5D	50.6
Xcfd40	5D	50.9
Xbarc49	5D	51.9
Xgdm138	5D	53.2
Xgwm561c	5D	54.2
Xmwg561c	5D	54.2
P35/M57-3	5D	56.2
Xgpw341	5D	56.2
D1.1_ctg4076	5D	57.2
Xcdo412B	5D	57.2
Xcfd8	5D	59.2
Xwmc405	5D	59.4
Xbcd1874	5D	60.1
Xgwm583	5D	60.4
D1.1_ctg12395	5D	61
D1.1_ctg233	5D	61
D1.1_ctg6927	5D	61
Xfbb156a	5D	61
PmY212	5D	61.1
Xgdm136	5D	61.6
D1.1_ctg605	5D	62.3
Xbarc286	5D	62.3
Xcdo57B	5D	62.3
Xgwm494	5D	62.3
XWC0008s	5D	63.3
Xcfd4c	5D	64.3
Xgwm639	5D	64.3
Xgdm43	5D	66.3
Xgpw303	5D	67.3

Xgwm182	5D	68.2
Xwmc630	5D	69
Xcfd26	5D	69.1
Xfba166b	5D	69.1
XWC0057s	5D	70.1
Xbarc347	5D	70.1
Xcfd101	5D	70.1
Xcfd7	5D	71.1
Xcfd3	5D	71.4
Xwmc818	5D	71.4
Xwmc574	5D	71.7
Xcfd12	5D	72.3
Xpsr806.3	5D	72.3
Xcfd102	5D	73
Xcfd57	5D	73
XgbxG083	5D	73.2
Xgpw5207	5D	73.2
Xgwm174	5D	73.5
Xcdo1508	5D	76.5
Xbarc320	5D	77.5
Xwmc289	5D	78.1
D1.1_ctg3162	5D	78.4
Xfbb26	5D	78.4
D1.1_ctg4878	5D	79.4
P36/M61-170	5D	79.4
Xabg3	5D	79.4
XWC0007ls	5D	80.4
Xwmc289b	5D	80.4
Ibf-D1b	5D	81
Pm35	5D	81
Xgwm121	5D	81.1
Xbarc361.2	5D	81.6
Xgwm271	5D	81.6
Xfba209c	5D	82.6
Xrz395.2	5D	83.6
M77/P64.8	5D	84.6
Xbcd450.2	5D	84.6
PmY201	5D	85.3
XgbxG333	5D	85.5
Xbarc93	5D	86.5
XgbxG070	5D	86.5
XgbxG722c	5D	86.5
XgbxR697	5D	86.5
Xgpw298	5D	86.5
P32/M48-237	5D	88.5

Xwmc215	5D	89.5
Xwmc264	5D	91.4
Xgwm292	5D	91.5
Xmwg900	5D	92.5
Xwmc95	5D	93.5
Xgwm212	5D	94.1
Xwmc434	5D	94.6
Xwmc434b	5D	95.2
Xcfd156	5D	96.6
Sr30	5D	97
Xcfd29	5D	98
Xbarc322	5D	99
Xcfd19	5D	99.9
QFhs.inra-5D	5D	100.1
Xwmc788	5D	100.3
Xcfd183	5D	102.9
Xmwg922	5D	104.6
Xwmc160	5D	104.6
Xwmc636	5D	104.6
Xwmc97	5D	105.1
Xcfd2i	5D	106
Xcfa2141	5D	106.9
Xbarc232	5D	108.9
M62/P64.9a	5D	109.2
Xwmc357	5D	109.2
Xgdm133	5D	111.6
Xcfd2	5D	113
Xbcd1103	5D	115.5
Xgdm63	5D	115.5
Xwmc206	5D	116.4
D1.1_ctg19	5D	119.4
D1.1_ctg8851	5D	119.4
P38/M47-1	5D	119.4
Xcdo346	5D	119.4
Xbarc110	5D	120.4
Xgpw343	5D	120.4
Xcfd86	5D	121.4
Xcfd283	5D	122.3
Xwmc640	5D	124.6
Xcfd10	5D	128.9
Xcfd2b	5D	130.3
Xopaf6-655	5D	130.3
XgbxR678	5D	131.3
Xwmc161b	5D	133.7
Xfbb100	5D	134.7

Xfbb213	5D	134.7
Xwmc161	5D	135.1
P38/M47-3	5D	135.9
Xgwm99.2	5D	135.9
Xwmc96	5D	137.9
Xgwm469	5D	138.5
Xbarc177	5D	139.7
Xbcd1670b	5D	139.7
Xwmc765	5D	140.3
Pm34	5D	142.4
P78/M92.9	5D	144.4
Xbarc144	5D	145.4
Xbcd1421	5D	145.4
Xbcd87a	5D	145.4
XWB144s	5D	146.4
Xfba364	5D	146.4
Xcfd25b	5D	147.4
Xfba11a	5D	147.4
Xgpw323	5D	147.4
Xgpw335	5D	147.4
Xpsr375.3	5D	147.4
Xpsr567.1	5D	147.4
Xgwm269	5D	147.4
Xgwm565	5D	147.6
D1.1_ctg3736	5D	148.6
D1.1_ctg8080	5D	148.6
M77/P64.10	5D	148.6
Xbcd197	5D	148.6
Xbcd87b	5D	148.6
Xcdo506	5D	148.6
Xfba11b	5D	148.6
Xgpw2323	5D	148.6
Xcdo1373a	5D	149.6
Xgwm272	5D	150.6
Xwmc443	5D	151.3
Xgwm654	5D	152.6
Lr1	5D	154.3
P35/M57-1	5D	155.6
P78/M87.7	6A	0
Xpsr899	6A	11
Xbcd342.1	6A	12
Xpsr889.2	6A	13
wPt-1377	6A	16.5
BQ805704	6A	16.6
XksuG8	6A	17.6

wPt-1664	6A	17.6
Xbcd1821.1	6A	18.6
Xfba152	6A	19.1
Xfbb222	6A	19.1
Xpsr167	6A	19.1
Xfbb47	6A	19.3
Xgwm459a	6A	19.5
wPt-0832	6A	20
wPt-9382	6A	20.1
XgbxG036b	6A	21.5
Xgpw2023	6A	21.5
Xfba65	6A	22.5
Xfbb194	6A	22.5
wPt-0562a	6A	23.3
Xgpw2295	6A	24
Xgpw2332	6A	24
Xutv1391	6A	24.6
Xbcd21a	6A	29.2
Xcdo476b	6A	29.2
Xfbb147	6A	29.2
Xgwm459	6A	29.2
Xfbb209	6A	30.2
Xpsr967	6A	32.2
XgbxR593	6A	33.2
Xgwm334	6A	36.2
Xwg487	6A	36.2
XCp3.2	6A	37.2
XA2437b	6A	38.2
Xwmc388	6A	38.2
wPt-4589	6A	38.3
wPt-4255	6A	38.5
Xmwg67.1	6A	39.1
wPt-3468	6A	39.6
wPt-4047	6A	39.9
Xcfd1	6A	39.9
Xcmwg652	6A	39.9
wPt-5395	6A	40.1
wPt-5633	6A	40.1
wPt-1742	6A	40.2
wPt-9832	6A	40.5
wPt-9306	6A	40.6
Xfbb166	6A	40.7
Xpsp302.3	6A	40.7
Xfbb255b	6A	41.7
XksuG48	6A	41.7

Xpsr8(Cxp3)	6A	41.7
Xmta15	6A	42.7
Xmtd184	6A	43.7
Xpsr10(Gli-2).1	6A	43.7
Xpsr10	6A	47.7
Xpsr119a	6A	47.7
wPt-4270	6A	47.7
XS25M55(90)	6A	48
wPt-6520	6A	48.2
wPt-7754	6A	48.3
wPt-0864	6A	48.5
wPt-6396	6A	48.5
P35/M61-6	6A	49.3
wPt-8006	6A	49.4
wPt-2636	6A	50
P42/M52-1	6A	51.3
Xfba85.1	6A	51.3
Xmwg652	6A	51.3
wPt-4017	6A	52.2
wPt-7330	6A	52.2
wPt-7840	6A	52.2
wPt-7938	6A	52.2
wPt-9075	6A	52.2
P39/M50-8	6A	52.3
Xabg466	6A	52.3
Xfba307a	6A	52.3
wPt-0495	6A	52.8
P40/M49-4	6A	53.3
Xmtd15	6A	53.3
wPt-2153	6A	54.2
wPt-5705	6A	54.9
Xfba152a	6A	55.3
Xgpw2082	6A	55.3
wPt-7565	6A	55.6
Xmag1200a	6A	56
XksuH4.2	6A	56.3
wPt-0689a	6A	57
wPt-3524	6A	57
wPt-8117	6A	57
wPt-8256	6A	57
wPt-9759	6A	57.2
Xmxe3M2o	6A	57.4
wPt-9679	6A	57.4
wPt-5652	6A	57.8
wPt-6904	6A	57.8

wPt-8848	6A	57.8
Xmwg573	6A	57.9
wPt-7027	6A	58
wPt-8266	6A	58.1
tPt-2833	6A	58.5
tPt-0877	6A	58.9
XS13M62(220)	6A	59
P38/M50-4	6A	59.3
Xmag4048	6A	60.4
Xgwm1040	6A	61.6
Xmwg573a	6A	62.3
Xpsr10(Gli-2).2	6A	62.3
wPt-7663	6A	62.5
P31/M54-5	6A	63.3
P33/M54-8	6A	63.3
P42/M50-4	6A	63.3
Xabg378a	6A	63.3
Xpsr949b	6A	63.5
CA681959a	6A	64.3
P41/M49-126	6A	65.2
Xutv1034	6A	65.7
wPt-9692	6A	66.2
wPt-3803	6A	67
P35/M61-4	6A	67.3
wPt-2822	6A	67.3
Xbarc206	6A	67.6
wPt-10629	6A	67.6
wPt-0259a	6A	67.7
wPt-3605a	6A	67.7
wPt-9584	6A	67.8
wPt-10558	6A	67.9
wPt-11347	6A	67.9
tPt-6278	6A	68.2
wPt-7486	6A	68.2
wPt-9205	6A	68.2
wPt-0338	6A	68.5
wPt-3965	6A	68.6
wPt-7623	6A	68.8
P38/M59-85	6A	73.7
Xcdo1315	6A	73.7
P33/M52-1	6A	76.7
wPt-7127	6A	77.7
Xcdo270.1	6A	77.8
wPt-9113	6A	79.6
Gli-A2	6A	79.7

P38/M59-122	6A	80.4
P39/M49-174	6A	80.4
P40/M60-153	6A	80.4
P41/M61-5	6A	80.4
wPt-7906	6A	81.3
XksuH4	6A	81.8
TC84464	6A	82
Xgwm825	6A	82
Xgpw2216	6A	82.2
Xgwm494a	6A	82.2
BE427655	6A	82.3
TC85125	6A	82.6
CA716967	6A	82.7
NP234852	6A	83
Xfba148	6A	83.2
csHPLC3	6A	83.2
BQ246417	6A	83.8
XE5M5p_R	6A	84.2
Xwmc630	6A	84.4
Xutv1035	6A	84.9
TC85303b	6A	85.1
Xgwm497	6A	85.5
Xgwm1009	6A	85.7
wPt-3091	6A	86.8
wPt-5931a	6A	87.3
wPt-7599a	6A	87.3
wPt-8833a	6A	87.3
TC84481a	6A	87.4
wPt-7445	6A	87.6
BJ261821	6A	87.8
XS25M60(380)	6A	88
wPt-5834	6A	88
Xfba85	6A	88.2
Xpsr627	6A	88.2
wPt-0959a	6A	88.6
Xbarc23	6A	88.7
wPt-2563	6A	89
wPt-0139	6A	89.1
Xmag1200b	6A	89.6
wPt-0357a	6A	90.1
XP39M37(250)	6A	90.7
Xmgb339	6A	91.9
wPt-9829	6A	91.9
wPt-10645	6A	92
wPt-11607	6A	92.1

P36/M47-6	6A	92.4
Xwmc182	6A	93
Xutv707	6A	93.3
P33/M54-3	6A	94
P35/M48-3	6A	94
P38/M61-7	6A	94
P41/M50-5	6A	94
Xpsr312	6A	94.1
Xmag1415	6A	94.3
Xfba234.1	6A	95
Xfbb145a	6A	95
BF483631	6A	95.5
Xutv1471	6A	96
Xbarc37	6A	96.7
Xrsq805	6A	97.5
Xgwm82	6A	97.6
Xgwm786	6A	97.9
Xbarc48	6A	98
Xbarc146	6A	98.1
Xwmc753	6A	98.1
Xwmc243	6A	98.1
Xbarc195	6A	98.3
Xcfd190	6A	98.5
Xcfd80	6A	98.5
Xwmc398	6A	98.6
wPt-5311	6A	99
Xwmc672	6A	99.2
Xutv1469	6A	99.4
Xbarc171	6A	99.5
Xpsp3152	6A	101.5
P31/M50-2	6A	102.5
Xcdo29	6A	102.5
Xgpw1150	6A	102.5
Xgpw2118c	6A	102.5
Xwmc145	6A	103.3
Xfbb95a	6A	103.4
Xgwm356	6A	103.9
Xwmc748	6A	104.1
Xwmc786	6A	104.2
Xwmc150	6A	104.7
Xwmc256	6A	104.7
Xwmc807	6A	105.3
Xgbx3321a	6A	105.5
XgbxG549	6A	106.5
XgbxG728	6A	106.5

Xbarc3	6A	107.9
Xbarc113	6A	108.4
XgbxR004	6A	108.4
Xcfd82	6A	109.4
Xbarc107	6A	110.4
Xpsr463	6A	111.4
XWB003df	6A	112.4
M60/P64.2	6A	114.4
M85/P65.3	6A	114.4
P40/M47-6	6A	114.4
Pm31	6A	114.4
XWB171f	6A	114.4
Xcdo1428	6A	114.4
Xrsq805.1(Embp)	6A	114.4
Xpsp3029.1	6A	115.4
Xpsp3071	6A	115.4
Xbcd506x	6A	116.4
Xfba397	6A	116.4
Xfbb95b	6A	116.4
Xgwm494b	6A	116.4
Xmta11	6A	116.4
Xwmc335	6A	116.5
P37/M47-6	6A	117.2
Xfbb215	6A	117.2
Xbarc204	6A	118.2
Xgpw2222	6A	118.2
P41/M48-304	6A	119.2
Xbcd758	6A	119.2
Xfba367	6A	119.2
Xfbb192	6A	119.2
Xfbb283b	6A	120.2
Xbcd1860	6A	121.2
Xcdo204	6A	121.2
Xcfd30	6A	122.2
XR61	6A	123.2
Xtam36	6A	123.2
Xpsr915	6A	123.2
Xwmc684	6A	123.2
Xcdo772	6A	124.2
Xwmc1	6A	124.2
Xfbb170	6A	126.2
Xgpw2060b	6A	126.2
S23M15_1	6A	128.2
Xcdo1373	6A	128.2
Xwmc201	6A	129.2

Xpsr142	6A	129.9
Xcdo388	6A	130.1
Xgwm907	6A	130.2
Xgwm132	6A	130.4
Xmtd11b	6A	131
Xwmc179j	6A	132
Xcsb112.1(Dhn5)	6A	133
Xmwg934b	6A	134
Xfbb164a	6A	136
Xgwm570	6A	136
tPt-4209	6A	136.2
Xabc175b	6A	136.7
Xwmc553	6A	137.2
wPt-7063	6A	137.8
Xwmc179	6A	139.5
Xwmc163	6A	140.1
wPt-8331	6A	144
Xfba111b	6A	162.3
wPt-3191a	6A	165.4
wPt-11612	6A	171.7
Xwmc417a	6A	172.5
tPt-3786	6A	173.1
Xcdo1091a	6A	176.2
Xbarc104	6A	177.2
Xcfa2114	6A	179.2
Xgwm169	6A	179.2
Xfbb221	6A	182.2
Xwmc417	6A	182.3
P31/M48-3	6A	183.2
Xfbb70c	6A	183.2
Xdupw167	6A	184
Xfbb82b	6A	184.2
Xgbx4071	6A	185.2
Xgpw2344	6A	185.2
S23M15_8	6A	186.2
Xfbb191	6A	186.2
XksuD12b	6A	186.2
Xopa7-520	6A	186.2
Xwmc580	6A	186.4
S13M25_5	6A	187.1
Xfba20	6A	187.1
Xfba8	6A	187.1
Xfbb40.1	6A	187.1
tPt-7761	6A	187.3
wPt-8373	6A	187.5

wPt-11668	6A	187.9
S23M15_6	6A	188.1
XksuG49b	6A	188.1
wPt-2877	6A	188.5
P35/M48-1	6A	188.7
Xgwm427	6A	188.7
wPt-6678	6A	189
wPt-7655	6A	189.2
S12M26_9	6A	190.1
S13M14_9	6A	190.1
Xbcd1510b	6A	190.1
tPt-6661	6A	190.5
Xgwm617	6A	191
Xwmc642	6A	192.2
wPt-6995	6A	194
wPt-7390	6A	194
wPt-5572	6A	194.2
wPt-1375	6A	194.8
S12M15_9	6A	195.3
S18M17_3	6A	195.3
XksuD27	6A	195.3
Xcdo836	6A	195.8
P35/M47-14	6A	197
P39/M49-238	6A	197
Xpsr546a	6A	197
wPt-0938	6A	200
wPt-4229	6A	200
wPt-8944	6A	200
wPt-9474	6A	200.2
F133	6A	200.3
P35/M57-4	6A	200.7
P40/M55-1	6A	200.7
Xmwg2053	6A	200.7
wPt-1661	6A	200.7
wPt-4445	6A	200.7
wPt-5331	6A	200.7
wPt-5696	6A	200.7
wPt-5787	6A	200.7
P41/M51-316	6A	201.7
P41/M58-90	6A	201.7
Xmwg573b	6A	201.7
Xmwg798	6A	201.7
wPt-2975	6A	201.9
wPt-8439	6A	202.4
wPt-9976	6A	202.4

S13M15_6	6A	203.3
Xgpw7388	6A	204.7
wPt-1642	6A	205
Swes953	6A	205.6
wPt-8954	6A	205.9
wPt-6829	6A	206.3
P31/M48-1	6A	206.5
MlRE	6A	208.1
Xgwm999	6A	208.3
Sr26	6A	208.5
Xgwm1089	6A	208.7
wPt-2632	6A	209.8
P31/M53-2	6A	211
wPt-8773	6A	213.2
P33/M52-8	6A	214
Xwmc621	6A	216.2
Xwmc206	6A	216.3
P38/M51-4	6A	218.8
Xwmc254	6A	254.3
Xwmc59	6A	263.3
wPt-9690	6B	0
wPt-2573	6B	1.2
wPt-0357b	6B	4.5
wPt-5931b	6B	5.2
wPt-8833b	6B	5.2
wPt-7599b	6B	7.2
wPt-0959b	6B	14.6
Xwmc430	6B	33.8
wPt-2991	6B	34.5
wPt-6154	6B	35.7
wPt-3774	6B	36.8
wPt-7662	6B	36.8
Xgwm1051	6B	37.8
XksuG8	6B	38
wPt-8894	6B	38.5
wPt-6127	6B	38.6
wPt-0151	6B	38.9
wPt-1089	6B	39
Xdupw217	6B	39.2
wPt-0882	6B	39.2
wPt-6293	6B	39.2
wPt-9406	6B	39.2
wPt-0562b	6B	39.3
wPt-9532	6B	39.3
wPt-8641	6B	39.4

tPt-0910	6B	39.5
wPt-0452	6B	39.5
wPt-1558	6B	39.5
wPt-1783	6B	39.5
wPt-1922	6B	39.5
wPt-2102	6B	39.5
wPt-2964	6B	39.5
wPt-3031	6B	39.5
wPt-3053	6B	39.5
wPt-3130	6B	39.5
wPt-4283	6B	39.5
wPt-4386	6B	39.5
wPt-5383	6B	39.5
wPt-6994	6B	39.5
wPt-7150	6B	39.5
wPt-7207	6B	39.5
wPt-8336	6B	39.5
wPt-8563	6B	39.5
wPt-8595	6B	39.5
wPt-9015	6B	39.5
wPt-9468	6B	39.5
wPt-9990	6B	39.5
tPt-2055	6B	39.6
wPt-0245	6B	39.6
wPt-2689	6B	39.6
wPt-4520	6B	39.6
wPt-3800	6B	40
wPt-7203	6B	40
wPt-8239	6B	40
wPt-4678	6B	40.1
wPt-4720	6B	40.3
wPt-1547	6B	40.4
wPt-2714	6B	41
wPt-7343	6B	41
wPt-3116	6B	41.2
wPt-3304	6B	41.2
wPt-5188b	6B	41.2
wPt-6282	6B	41.2
wPt-2133	6B	41.4
wPt-1852	6B	41.7
wPt-7777	6B	41.7
wPt-0351	6B	41.9
Xpsr119	6B	42.5
wPt-6988	6B	42.7
wPt-1474	6B	43

wPt-8015	6B	43.8
wPt-4233	6B	45.8
wPt-4706	6B	46.3
wPt-7954	6B	47.4
wPt-1437	6B	49.1
wPt-2095	6B	49.1
wPt-3424	6B	49.1
wPt-9487	6B	49.4
Xgwm131	6B	49.8
wPt-3118	6B	50.1
XP39M37(680)	6B	50.4
wPt-4900	6B	50.5
wPt-9492	6B	50.6
wPt-6916	6B	50.8
wPt-6653	6B	50.9
wPt-4564	6B	51.2
wPt-1725	6B	51.3
Xgwm613a	6B	55.8
wPt-0259b	6B	57.7
Xwmc419	6B	59.2
wPt-3376	6B	59.6
Xwmc486	6B	60
XS13M50(390)	6B	60.8
wPt-2432	6B	60.9
wPt-5256	6B	61.5
Xbarc76b	6B	61.7
Xbarc76	6B	61.9
wPt-1241	6B	62.3
wPt-8814	6B	62.8
wPt-3605b	6B	63.2
wPt-2786	6B	64
wPt-2175	6B	64.7
Xwmc487	6B	65.2
wPt-7745	6B	65.2
XksuH4	6B	65.6
wPt-4858	6B	65.8
Gli-B2	6B	67.1
S25M19_7	6B	67.2
Xgpw93017	6B	67.9
Xcfd13a	6B	68
wPt-8153	6B	68.1
TC85035	6B	68.4
Xbarc18	6B	68.9
S18M13_3	6B	78
S25M19_9	6B	80

S25M19_6	6B	83
Xgwm132	6B	87
Xgwm613b	6B	87
wPt-6942	6B	87
Xpsr167a(Hpr)	6B	88
S24M17_1	6B	91
Xgpw1079	6B	91
Xcdo476.2	6B	92
Xgwm705	6B	92.1
Xwmc104	6B	92.1
wPt-2297	6B	92.2
wPt-0470	6B	92.5
wPt-3309	6B	93.1
wPt-5333	6B	93.1
wPt-4742	6B	93.3
wPt-9971	6B	93.8
BJ236800	6B	93.9
wPt-0554	6B	94
CA741546	6B	94.2
wPt-7540	6B	94.3
Xcfd1	6B	94.4
XS13M61(290)	6B	94.5
wPt-2479	6B	94.6
wPt-9667	6B	94.6
wPt-1788	6B	94.8
wPt-2587	6B	94.8
Xak466.1(Nra)	6B	95.2
Xcdo1315	6B	95.2
tPt-2451	6B	95.3
wPt-3163	6B	95.7
TC85303a	6B	96
S23M15_6	6B	96.1
XgbxG084	6B	96.1
wPt-1185	6B	97
XS25M49(120)	6B	97.2
wPt-2218	6B	97.3
TC85307	6B	97.6
Xutv1280	6B	97.9
wPt-9594	6B	97.9
Xfba344	6B	98.5
XgbxG103	6B	98.6
Xpsr312	6B	98.9
TC85037	6B	99.3
S12M17_10	6B	99.5
Xpsr136	6B	99.5

S23M15_9	6B	100.5
S23M25_3	6B	100.5
S23M26_1	6B	100.5
S23M26_2	6B	100.5
Xgbx3165a	6B	100.5
XgpwE1	6B	100.5
TC84481b	6B	101
TC65966	6B	101.3
S12M17_9	6B	101.4
XgbxG083	6B	101.4
wPt-0052	6B	102
wPt-8194	6B	102
Xbcd342A	6B	102.3
Nor2	6B	102.3
wPt-7846	6B	102.4
wPt-9659	6B	104
Xubp74	6B	104.2
wPt-10467	6B	104.2
TC80528a	6B	104.7
M51/P65.1	6B	104.9
P78/M63.1	6B	104.9
S13M15_11	6B	104.9
S23M20_14	6B	104.9
Xbarc48	6B	104.9
wPt-3666	6B	105.2
Pm27	6B	105.9
Xpsp3131	6B	105.9
Xbarc68	6B	106
S24M15_9	6B	106.7
Xcdo365	6B	106.7
S25M14_3	6B	107.7
Gpc-B1	6B	108.2
Xgdm113	6B	110.7
Xwmc95	6B	110.7
Xgwm518	6B	111.4
S13M25_2	6B	112.4
S12M17_3	6B	114.4
wPt-1700	6B	114.8
XksuD17	6B	115.1
Xwmc597	6B	115.6
Xrz995	6B	116.1
Xwmc494	6B	116.1
wPt-7489	6B	116.1
XgpwE3	6B	117.1
Xbcd20C	6B	120.1

Xcdo270	6B	120.1
wPt-8721	6B	120.9
Xbcd1407a	6B	121
Xcdo1473	6B	122
Xfbb16	6B	122
wPt-3733	6B	123
Xbcd2014a	6B	124.7
Xfba152	6B	124.7
Xpsp3009	6B	124.7
Xtam60	6B	124.7
Xgpw2060a	6B	126.7
Xgwm816	6B	126.7
Xgwm508	6B	126.7
wPt-3606	6B	126.8
BJ213673b	6B	128.3
Xgwm191	6B	128.3
S12M26_7	6B	128.7
Xcdo507a	6B	128.7
Xfba357	6B	128.7
Xrz273	6B	128.7
Xwg908.1	6B	128.7
Xfbb61	6B	128.8
wPt-0446	6B	129.2
wPt-9784	6B	129.2
Xwmc265	6B	129.7
Xwmc737	6B	129.8
S12M26_6	6B	129.8
XgbxG176	6B	129.8
Xwmc398	6B	130.7
Xgwm193a	6B	130.9
CA594434b	6B	131
wPt-3060	6B	131.2
S12M18_2	6B	131.5
Xgwm133	6B	131.5
Xgwm644a	6B	132.8
wPt-8183	6B	133.2
M86/P65.5	6B	133.7
P78/M72.7	6B	133.7
Xwmc388	6B	133.7
Xfba345a	6B	134.7
Xbcd1383a	6B	135.7
Xpsr141a	6B	135.7
Xgwm361	6B	135.7
S23M25_10	6B	136.7
Xcdo1421	6B	136.7

Xcdo524	6B	136.7
wPt-2000	6B	136.9
Xwmc397	6B	137
Xwmc756	6B	137
Xwmc105	6B	137.1
Xgwm88	6B	137.8
XS25M55(600)	6B	137.9
BF202619-86	6B	138
Xgwm273	6B	138
Xwmc179	6B	138
Xwmc473	6B	138
Xgwm58	6B	138
wPt-2564	6B	138
wPt-7935	6B	138
Xgwm70	6B	138.7
Xbcd102.1	6B	140.7
XgbxG138	6B	140.7
Xgwm518b	6B	140.7
Xbcd1398	6B	141.1
Xbarc146	6B	141.2
TC101037	6B	141.3
Xfba328a	6B	141.4
Xwmc182	6B	141.4
Pm12	6B	141.7
Xbarc198	6B	141.7
Xcfa2110	6B	142
Xwmc726	6B	142.2
Xwmc79	6B	142.4
Xbcd1299a	6B	142.7
Xgwm191.1	6B	142.7
XksuH14a	6B	142.7
Xpsp3118	6B	142.7
Xgwm311	6B	142.7
Xgwm608	6B	143
XWB101ib	6B	144
Xrz476a	6B	144
M85/P65.6	6B	145
Xfba67a	6B	145
Xwmc105y	6B	145
Xwmc387	6B	145
Xcnl3c	6B	146
Xfba399	6B	146
P40/M56-2	6B	147
P41/M47-1	6B	147
P41/M58-206	6B	147

Xbcd1716	6B	147
Xbcd357	6B	147
Xmwg900a	6B	147
S18M17_1	6B	148
Xfba359	6B	148
rPt-1040	6B	148.2
wPt-2424	6B	148.3
Xwmc748	6B	148.8
wPt-5037	6B	148.8
wPt-9124	6B	148.8
M65/P64.1	6B	149
Nor-B2	6B	149
Xwmc786	6B	149.3
M65/P64.10	6B	149.9
S23M26_6	6B	149.9
Xbcd1299b	6B	149.9
Xbcd1495	6B	149.9
XAhas	6B	150.9
Xcfd13b	6B	150.9
Xpsr8(Cxp3)	6B	150.9
Xbcd1426	6B	150.9
P34/M51-229	6B	151.9
Xbcd2014b	6B	151.9
Xgbx3321b	6B	151.9
Xgwm133b	6B	151.9
P78/M39.5	6B	152.9
P78/M71.3	6B	152.9
Xgwm325	6B	152.9
Xisu97	6B	152.9
P32/M51-6	6B	153.9
P36/M50-2	6B	153.9
Xfbb364	6B	153.9
Xpsr908	6B	153.9
Yr36	6B	153.9
P78/M87.5a	6B	154.9
Xcdo507b	6B	154.9
Xgbx3327	6B	154.9
Xgpw1017	6B	154.9
Xpsr167	6B	154.9
Xbarc127	6B	155.2
Xwmc539	6B	155.5
Xglk479	6B	155.8
P41/M47-2	6B	156.8
P41/M48-136	6B	156.8
XksuG8a	6B	156.8

XuaWM1.1	6B	156.8
QHtap.wsu-6B.1	6B	157.8
Xbcd1407b	6B	157.8
Xwg232b	6B	157.8
Xgwm644b	6B	157.8
Xbcd1398b	6B	158.8
P41/M58-260	6B	159.8
Xwg286	6B	159.8
Xgwm626	6B	159.8
S23M14_5	6B	160.8
S24M17_5	6B	160.8
Xcdo772	6B	161.8
Xgwm887	6B	162
Xpsr915	6B	162.5
CA677684b	6B	162.6
wPt-3191b	6B	162.6
wPt-0397	6B	162.7
tPt-9048	6B	163.4
wPt-1761	6B	163.6
wPt-9241	6B	163.6
Xwmc152	6B	163.7
F138	6B	163.9
wPt-2582	6B	163.9
wPt-3581	6B	163.9
wPt-9881	6B	163.9
wPt-2162	6B	164
Xbarc79	6B	164.7
Xbarc354	6B	165.3
Xgwm193b	6B	165.3
Xpsp3112.2	6B	165.3
wPt-8508	6B	165.3
wPt-8554	6B	165.3
P33/M52-3	6B	166.3
Xpsr141g	6B	166.3
Xbcd1383b	6B	167.3
Xabc175a	6B	168.3
XksuH14b	6B	168.3
P34/M51-219	6B	169.3
P35/M49-4	6B	169.3
Xfba328b	6B	169.3
Xfba345b	6B	169.3
Xfba67b	6B	170.3
wPt-1730	6B	170.6
Xwmc182b	6B	171.3
Xgwm107	6B	171.4

wPt-4924	6B	171.4
Xfbb156b	6B	172.2
Xgbx3213	6B	172.2
wPt-1287	6B	172.8
Xmwg934a	6B	173.1
wPt-7627	6B	173.2
P78/M43.13	6B	174.1
Xcdo341b	6B	174.1
Xgwm907	6B	175
Xwg341	6B	175
Xbarc24	6B	176
P35/M61-2	6B	177
Xpsr2(alpha-Amy-1).1	6B	177
XWB178m	6B	178
Xbarc178	6B	178
Xfba42	6B	179
P40/M61-1	6B	180
Xgpw1014	6B	180
XksuG30	6B	180
Xabg473.2	6B	181
Xfbb130	6B	181
Xcdo1091b	6B	182
Xfbb164c	6B	183
Xgwm570	6B	183
Xfbb169x	6B	184
Xdupw216	6B	184.8
XksuG12	6B	185.8
Xwmc417	6B	186.1
Xfbb377c	6B	186.9
Xgwm963	6B	188.5
S13M14_10	6B	188.7
Xfbb57	6B	188.7
Xcdo836	6B	189.7
Xpsr546b	6B	190.7
Xfba111	6B	191
Xgwm219	6B	191
wPt-9930	6B	192.6
Xfbb327	6B	192.9
Xfbb359b	6B	192.9
P32/M54-2	6B	193.9
Xwmc417b	6B	194.9
Xbarc361	6B	195
P38/M50-1	6B	196.1
XWD124	6B	196.1
wPt-6441	6B	196.3

Xgwm889	6B	196.4
Xfbb251x	6B	197.1
Xgwm768	6B	197.6
wPt-9270	6B	198
P78/M63.3a	6B	198.1
Xfba127	6B	198.1
Xfbb221b	6B	199.1
Xfbb82a	6B	199.1
wPt-7830a	6B	200
Xmwg74	6B	200.1
tPt-9948a	6B	200.8
Xfbb250b	6B	201.1
PmG3M	6B	201.2
wPt-1048	6B	201.3
wPt-8418b	6B	201.3
wPt-6184	6B	201.5
tPt-6794	6B	202.2
wPt-5092	6B	202.2
wPt-5101	6B	202.2
wPt-5257	6B	202.2
Xfbb70.2	6B	202.5
Xfba251	6B	203.5
Xfbb59c	6B	203.5
wPt-7274	6B	204
wPt-8149	6B	204.2
wPt-2523	6B	204.3
wPt-3059	6B	204.3
wPt-6967	6B	204.3
wPt-8043	6B	204.3
wPt-3572a	6B	204.4
Xgwm608b	6B	204.5
Xmwg2053a	6B	204.5
wPt-1885	6B	204.5
wPt-9481	6B	204.7
Xbarc134	6B	204.8
wPt-2178	6B	205.5
Xgwm735	6B	206
wPt-9952	6B	209.8
wPt-1325	6B	212
S24M15_4	6B	236.7
wPt-6329	6B	251.3
wPt-0171	6B	255.1
wPt-1264	6B	256.4
wPt-3045	6B	256.4
wPt-5885	6B	256.4

wPt-9423c	6B	256.4
wPt-5480	6B	260
wPt-5176	6B	261.5
wPt-1541	6B	264
XWC0049t	6D	0
Xpsr889	6D	0
P31/M51-1	6D	6
P40/M51-7	6D	6
Xpsp3200	6D	6
Xpsr899b	6D	6
Xbcd1821.2	6D	7
Xgbx3165b	6D	9
D1.1_ctg3913	6D	10
D1.1_ctg6315	6D	10
Xbcd342.3	6D	10
Xbcd1821a	6D	11
Xabg466	6D	12
Xcfd49	6D	15
Xbarc183	6D	18.1
Xcfd135	6D	20.1
Xfba307.2	6D	23.1
Xbarc173	6D	26.1
Xcfd213a	6D	28.1
D1.1_ctg5153	6D	32.1
Xgwm132	6D	32.1
Xpsr964	6D	32.1
Xcfd75	6D	33.5
XWB173t	6D	33.9
Xgdm132	6D	34.5
XCp3.1	6D	36.5
Stb3	6D	40.5
XksuG48	6D	40.5
Xfbb130a	6D	45.5
Xmwg549.2	6D	47.5
D1.1_ctg7312	6D	49.5
D1.1_ctg9360	6D	49.5
Xfba1	6D	49.5
Xcfd13	6D	49.5
Xcfd1	6D	51.3
Xcfd42	6D	52.1
Xgwm469	6D	53.5
Xwmc749	6D	55.2
D1.1_ctg4968_1	6D	55.5
Xfba85.2	6D	55.5
H13	6D	56.5

Xcfd213b	6D	56.5
Xcfd132	6D	56.5
Xgdm141	6D	57.5
Xgpw1034	6D	59.5
XgpwE10	6D	61.5
XgpwE6	6D	61.5
Xfba187	6D	63.5
D1.1_ctg7851	6D	66.5
Xcfd33	6D	66.5
Xfbb319a	6D	66.5
Xfbb354	6D	66.5
D1.1_ctg4968	6D	67.5
Xfba85a	6D	67.5
Xfbb222	6D	69.5
Xbarc54	6D	70.4
Xfbb231a	6D	71.5
Xfbb283a	6D	73.5
Xwmc113x	6D	73.5
Xpsr106	6D	75.5
Xcfd19	6D	76.5
D1.1_ctg57	6D	77.8
Xpsr627	6D	77.8
Xbarc196	6D	77.8
Xmwg916	6D	79.8
Xgwm325	6D	83.3
Xcfd190	6D	85.3
D1.1_ctg2309	6D	85.4
Xcdo270A	6D	85.4
Xwmc416	6D	85.4
Xabc173a	6D	86.4
Xbarc23.1	6D	88.4
Xbarc123	6D	89.4
Xbarc202	6D	89.4
Xfba336	6D	90.4
Xcdo534a	6D	91.4
Xgwm55	6D	91.4
Xbcd1398	6D	92.4
Xgwm133	6D	93.1
Xgpw1101	6D	93.3
Xbarc5	6D	93.5
Xwmc822	6D	93.5
Xgpw304	6D	94.3
D1.1_ctg1399	6D	95.3
D1.1_ctg1580	6D	95.3
D1.1_ctg7974	6D	95.3

Xgpw1019	6D	95.3
Xpsr371	6D	95.3
Xtam31	6D	95.3
Xbcd1716.2	6D	96.3
Xbcd357.2	6D	96.3
Xcfd188	6D	96.3
Xcfd37	6D	96.3
Xcfd80	6D	96.3
Xwmc753	6D	97.6
Xcfd76	6D	97.7
Xwmc469	6D	97.7
Xcfd219	6D	97.8
PmD57-6D	6D	98.9
Xcfd287	6D	102
QHt.inra-6D	6D	103
D1.1_ctg3200_1	6D	104
D1.1_ctg3888	6D	104
D1.1_ctg5394	6D	104
Xwg933	6D	104
Xcfd47	6D	105
Xgwm582a	6D	106
Xwmc786	6D	106.9
D1.1_ctg3633	6D	107.9
Xabc175	6D	107.9
Xgpw306	6D	107.9
Xwmc748	6D	107.9
P43/M62-1	6D	108.8
Xbarc273	6D	110.8
Xfba381	6D	111.8
Xbarc204	6D	113.8
P40/M56-4	6D	118.3
QGwt.crc-6D	6D	118.3
Xcfd95	6D	118.3
Xbarc175	6D	118.3
D1.1_ctg1630_1	6D	119.3
D1.1_ctg3802_1	6D	119.3
D1.1_ctg3818_1	6D	119.3
D1.1_ctg5856_1	6D	119.3
Xfbb169a	6D	119.3
Xbarc1121	6D	120.3
D1.1_ctg5362	6D	121.3
Xabg20	6D	121.3
Xgwm55b	6D	121.3
Xcdo534b	6D	122.3
Xfba336b	6D	122.3

wPt-2782	6D	122.6
D1.1_ctg2065	6D	123.3
D1.1_ctg835	6D	123.3
D1.1_ctg8667	6D	123.3
Xcmwg669	6D	123.3
Xglk547	6D	124.3
Xabc173b	6D	125.3
Xtam28a	6D	127.3
Xcsb112.2(Dhn5)	6D	128.3
wPt-1314	6D	129.7
Xbarc96	6D	131.3
Xfbb231b	6D	131.5
D1.1_ctg3200_2	6D	135.5
Xfba81	6D	135.5
D1.1_ctg9019	6D	140.5
Xcfd38	6D	140.5
XksuE14b	6D	140.5
D1.1_ctg3096	6D	143.5
XksuD1	6D	143.5
Xgpw2232	6D	146.5
Xmtd11a	6D	146.5
Xcdo836a	6D	147.5
Xwmc773	6D	149.3
Xgpw312	6D	149.5
D1.1_ctg1630_2	6D	151.5
D1.1_ctg3802_2	6D	151.5
D1.1_ctg3818_2	6D	151.5
D1.1_ctg5856_2	6D	151.5
Xfbb169b	6D	151.5
Xcfd45	6D	153.5
Xmwg669	6D	154.5
Xfba76	6D	156.5
Xmtd12	6D	156.5
Xopad12	6D	156.5
Xtam28b	6D	157.5
Xcsb112(Dhn5)	6D	158.5
Xbcd1319	6D	160.5
Xgpw362	6D	160.5
Xfba26a	6D	161.5
Xbcd1510a	6D	164.5
Xcfd5	6D	165.5
Xcmwg684a	6D	166.5
Xgbx3317	6D	167.5
P36/M61-90	6D	168.5
Xcdo836b	6D	168.5

P32/M52-290	6D	169.5
Xbarc21	6D	170.5
XksuD27a	6D	170.5
P34/M47-1	6D	171.5
Xcfd60	6D	171.5
D1.1_ctg6151	6D	172.5
XksuD12a	6D	172.5
D1.1_ctg1004	6D	175.5
D1.1_ctg5649	6D	175.5
Xfbb59a	6D	175.5
Xfbb70b	6D	175.5
XksuE3a	6D	175.5
Xmwg2053c	6D	177.5
XMXE3M8o	6D	180.5
P39/M50-6	6D	187.5
P35/M48-7	6D	200.5
P35/M47-8	6D	201.5
XscuM01	6D	206.5
P40/M47-5	6D	213.5
Xgdm98-6D	6D	218.5
Xgwm666	7A	0
Xfba17	7A	3.8
Xfba321	7A	3.8
Xfba322	7A	3.8
Xfba65s	7A	3.8
Xfbb278d	7A	3.8
XgbxG367b	7A	3.8
XgbxR799b	7A	3.8
Xgpw2107	7A	3.8
Xgpw2233	7A	3.8
Xgpw2297	7A	3.8
Xfba311a	7A	4.8
Xfba72	7A	4.8
Xfba93	7A	4.8
Xfbb121	7A	4.8
Xfbb156c	7A	4.8
Xfbb193	7A	4.8
Xfbb9b	7A	4.8
Xgwm681	7A	4.8
Xgwm233	7A	5.8
Xopai5-650	7A	5.8
Xpsr119a	7A	6.8
Xwmc158	7A	6.9
M83/P65.4	7A	7.7
P78/M69.1	7A	7.7

Xcdo545	7A	7.7
Xpsr563.2	7A	7.7
Xbcd907	7A	8.7
QHt.inra-7A	7A	9.7
Gby	7A	12.7
P78/M63.6	7A	12.7
P78/M72.2	7A	13.7
Xfbb67a	7A	13.7
Xpsr490.3(Ss1)	7A	13.7
Xfba127	7A	14.7
Xpsr386.2	7A	14.7
Xfbb186	7A	15.7
XgbxG564a	7A	15.7
Xcdo475	7A	16.7
Xfba109	7A	16.7
Xwmc388	7A	16.7
M65/P64.2a	7A	17.7
Xglk61.2	7A	18.7
Xgwm635	7A	20.6
Xbarc70	7A	21.3
Xpsr558	7A	23.1
wPt-9496	7A	26.2
Xgwm350	7A	26.3
Xfba42a	7A	27.3
Xfba346	7A	28.3
Xgwm350b	7A	28.3
Xbarc151	7A	29
Xwmc497	7A	29.2
Xgwm526	7A	30.2
Xwmc646	7A	30.9
Xgwm471	7A	35.8
wPt-4487b	7A	36.2
wPt-6966	7A	36.6
wPt-1441	7A	37.9
wPt-0393	7A	38.4
wPt-7653a	7A	38.8
wPt-9207	7A	38.8
wPt-3135	7A	39.1
wPt-5964	7A	40.5
wPt-3648	7A	41
wPt-7491b	7A	43.1
wPt-3434	7A	43.6
Xwmc479	7A	49.2
wPt-6824	7A	49.3
Xgwm834	7A	53.2

Xwmc168	7A	55.8
wPt-5926	7A	56.8
wPt-11672	7A	63.1
wPt-7830b	7A	63.6
wPt-7188	7A	65.1
wPt-4714	7A	66.4
TC67645	7A	67.7
tPt-9948b	7A	67.9
Xgwm60	7A	69
Xsun1-280	7A	69
Xabc158	7A	73
Xfba248	7A	73
Xgwm130	7A	73.6
Xcfd13	7A	75.2
Xwmc593	7A	75.2
wPt-6034	7A	77.4
Xwmc179d	7A	77.8
Xcfd242	7A	79.1
Xbcd1066a	7A	80.2
Xgbx3480a	7A	82.2
Xfba337a	7A	83.2
Xcdo962A	7A	85.2
Xfbb218a	7A	87.2
Xcfa2049	7A	88.2
Xgpw2119	7A	88.2
Lr47	7A	90.2
Xfba248b	7A	90.2
Xfbb166b	7A	90.2
Xwg834c	7A	90.2
wPt-8789	7A	90.5
wPt-7151	7A	90.8
Xgpw2103	7A	91.1
Xwmc179	7A	91.1
QGw1.inra-7A	7A	92
wPt-1163	7A	92.2
Xbcd873	7A	93.4
Xgwm276a	7A	93.8
Xgwm60b	7A	94.8
XksuG12a	7A	94.8
wPt-6668	7A	95.3
Xbarc1034	7A	95.5
wPt-3220	7A	95.9
wPt-7299	7A	96.1
Xwmc283	7A	96.3
Xwmc83a	7A	99.3

wPt-9314	7A	99.4
wPt-4699b	7A	99.5
wPt-8650b	7A	100.2
wPt-9796	7A	100.7
wPt-6447	7A	102.1
wPt-4835	7A	102.8
Xbarc127	7A	103
wPt-7214	7A	103.2
Xbarc154	7A	103.4
Xbcd135	7A	105.1
wPt-2678	7A	105.7
Xcfa2028	7A	106
wPt-0008	7A	106.3
wPt-7034	7A	106.8
wPt-3572b	7A	107
wPt-4345	7A	107.1
XWC2028g	7A	107.8
mlRd30.1	7A	108.8
wPt-1917	7A	108.9
Xfba340	7A	109.7
Xpsr952	7A	109.7
wPt-1080	7A	110.3
wPt-7076	7A	110.9
wPt-5232	7A	111.2
wPt-4880	7A	111.4
P78/M49.4	7A	111.4
Xgwm631	7A	112.6
wPt-0288	7A	113.3
wPt-3373	7A	113.3
TC77994	7A	116.6
P78/M49.7	7A	118.8
Xcfa2174a	7A	123.8
Xfbb264	7A	124.8
Xwmc83	7A	126.8
Xwmc405	7A	130.1
Xwmc826	7A	131.5
Xgwm900	7A	135.1
Xbarc174	7A	137.3
TC77993	7A	138.7
Xcdo475b	7A	140.7
Xbarc23	7A	140.8
Xcfd6c	7A	141.4
wPt-1510	7A	141.9
XOA85	7A	142.7
Xgwm573	7A	142.7

XksuG12b	7A	143.7
XgbxG575a	7A	145.7
wPt-0205	7A	147.2
Xbarc21	7A	147.4
M85/P65.1	7A	147.5
Xfba354a	7A	147.5
wPt-3393	7A	148.1
Xbcd1066b	7A	150.4
Xglk356	7A	150.4
Xwmc17	7A	151.4
Xfba69a	7A	152.4
Xgdm14	7A	152.4
Xcdo17	7A	153.4
Xgpw2083a	7A	153.4
XWB174ha	7A	155.4
Xfbb343	7A	155.4
Xgpw2092	7A	156.4
Xgwm260	7A	156.4
Xsfr.BF475036	7A	157.4
P1	7A	158.4
Xfba97a	7A	159.4
Xbarc195	7A	160.1
Xopa10-480	7A	160.3
Xbarc49	7A	160.5
wPt-4637	7A	161.5
Xbarc29	7A	162.1
Xbarc108	7A	162.8
Xfbb145a	7A	162.9
Xfbb18a	7A	162.9
Xcdo1395	7A	163.2
Xwmc182	7A	163.2
Xwmc695	7A	163.2
Xwmc596	7A	163.6
Xwmc422	7A	163.9
Xwmc65	7A	164.6
wPt-8399	7A	164.7
wPt-4796	7A	165.1
wPt-4744	7A	165.4
M21/P76.12	7A	165.6
M67/P77.4	7A	165.6
Xfba337b	7A	165.6
Xra1-350	7A	165.6
wPt-3992	7A	165.7
Xwmc603	7A	165.9
Xfba234	7A	166.5

Xfbb218b	7A	167.5
Xfba382a	7A	168.5
Xra1-670	7A	168.5
XWB108g	7A	169.5
Xwmc143	7A	169.5
Xcdo962B	7A	172.5
Xpsp3050	7A	173.5
Xwmc107	7A	174.5
Xwmc9	7A	174.5
Xbarc121	7A	177.3
wPt-6842	7A	177.6
Xwmc607	7A	178.5
Xwmc139	7A	179.1
Xgwm10	7A	179.9
Xwmc488	7A	179.9
Xgwm4	7A	180.1
Xcfd193	7A	182.6
wPt-4877	7A	183.2
wPt-6217	7A	183.2
CA668788b	7A	183.4
Xgwm748	7A	183.4
Xdupw254	7A	183.5
CA668775	7A	184.1
Xgwm276	7A	185.1
XksuG12c	7A	193.1
Xdupw226	7A	193.3
Xwmc286	7A	195.8
M21/P76.6	7A	197.8
wPt-3692	7A	200
wPt-0433	7A	201.3
Xfba354b	7A	201.4
Xfba69	7A	203.1
Xcfa2257	7A	209.3
Xcfd20b	7A	209.3
Xcdo595	7A	209.5
Xpsr311	7A	209.5
wPt-5949	7A	210.6
wPt-7053	7A	210.7
Xwmc247	7A	210.8
Xcdo673	7A	210.9
Xwmc426	7A	211
Xcfd20	7A	211.7
P78/M49.2a	7A	214.6
Xwg23.6	7A	217.6
Xfba69b	7A	219.6

wPt-4731	7A	220.3
Xfba97b	7A	222.3
XgbxG411a	7A	224.3
M51/P65.7	7A	225.3
Xbarc192	7A	225.4
Xgwm1017	7A	225.6
Xgwm282	7A	227.8
Sr22	7A	228.8
Xgwm332a	7A	228.8
Xfbb145b	7A	232.8
Xfbb18b	7A	232.8
Xrz682	7A	239.8
Xmwg938	7A	240.8
XksuD2	7A	245.8
Xfba204	7A	249.8
Xwg380	7A	251.8
Xfba350	7A	256.8
Xgwm332	7A	259.8
Xpsp3094.1	7A	259.8
Pm37	7A	261.7
Xgwm746	7A	262.2
Xgpw2252	7A	263.5
Xwmc790	7A	263.7
Xgwm63	7A	266.5
Xwmc633	7A	268.9
wPt-0961	7A	270.3
wPt-5524	7A	270.5
wPt-4553	7A	271.1
wPt-6620	7A	272.6
wPt-4831	7A	272.7
BJ262177c	7A	273.7
Xcfd6	7A	278.2
NCA6Pm	7A	281.1
Xcfa2019	7A	281.1
wPt-9808	7A	282.4
Xgwm554	7A	286.4
wPt-6869a	7A	289.2
Xwmc116	7A	290.7
PmNCAG11	7A	290.8
wPt-7219a	7A	290.8
wPt-3439a	7A	291
Xcfa2040a	7A	291.2
wPt-1976	7A	293
wPt-3403	7A	293.1
wPt-8534	7A	293.1

BJ262177b	7A	293.6
Xmag1759	7A	295.1
Xmag2185	7A	295.5
Mlm80	7A	296.3
Mlm2033	7A	297
Xwmc273	7A	297.2
Xgwm344a	7A	297.3
PmG16	7A	298
M21/P76.13	7A	298.6
P78/M59.5	7A	298.6
P78/M71.8	7A	298.6
Xpsp3001.1	7A	298.6
Xpsr148	7A	298.6
wPt-6797	7A	298.9
MlIW72	7A	299.1
wPt-3154	7A	299.1
Xgwm1061	7A	299.4
Pm22(Pm1e)	7A	299.6
P32M51	7A	299.9
wPt-6276a	7A	301.2
M62/P64.8	7A	301.3
PmNCA4	7A	302.5
wPt-8365	7A	302.5
Xcfa2240	7A	302.7
wPt-11370	7A	303
wPt-5940	7A	304.2
wPt-1429	7A	305.1
wPt-9217	7A	305.2
wPt-8846	7A	305.8
wPt-0551	7A	306
wPt-5724	7A	306.2
wPt-5690	7A	307.9
P78/M68.6	7A	309.3
mlRD30	7A	309.4
XgbxG218a	7A	311.7
Xwmc525	7A	311.7
wPt-3226	7A	313.4
Xfba349	7A	313.8
wPt-2260	7A	313.8
TC92445	7A	313.9
Xgwm698	7A	316.8
Lr20	7A	321.6
Sr15	7A	321.6
Xpsr687	7A	321.6
Xpwir232b	7A	321.6

Pm1	7A	321.6
Xcdo347	7A	321.6
Xgwm344b	7A	321.6
Xpsr680a	7A	321.6
wPt-1601c	7A	322.7
Xpsr121	7A	323.2
Xcfa2040	7A	323.6
XksuH9	7A	323.9
wPt-5533	7A	324.8
wPt-1023	7A	324.9
wPt-6019	7A	324.9
wPt-4220	7A	325.1
wPt-4319a	7A	325.3
wPt-6495	7A	325.6
wPt-7763	7A	325.6
wPt-7947	7A	326.6
wPt-2501	7A	327
MlaG12	7A	328.3
wPt-0689b	7A	328.6
PmU	7A	338.2
Xwmc809	7A	347.5
CA594434c	7B	0
Xwmc597	7B	0
Xgwm263	7B	19.1
Xgwm255	7B	20.6
Xwmc606	7B	23.7
Xgwm46a	7B	24.9
Xwmc323	7B	24.9
wPt-7975	7B	25.2
wPt-5283	7B	26.1
wPt-0276	7B	29.4
wPt-3147	7B	29.7
P31/M52-1	7B	32.2
Xgwm569	7B	32.2
wPt-8920b	7B	37.6
wPt-5846	7B	37.9
wPt-9800c	7B	38
BJ239878	7B	47.5
wPt-2278	7B	50.7
Xwmc338	7B	52.5
wPt-4309	7B	53.1
wPt-1853	7B	55.9
Xfba311a	7B	58.7
Xgpw2233	7B	58.7
P35/M52-4	7B	59.7

Xfba72	7B	59.7
XgbxG367b	7B	59.7
Xgpw2297	7B	60.7
Xgwm233	7B	60.7
Xgwm350	7B	60.7
Xbcd907b	7B	61.7
Xcdo545b	7B	61.7
Xfba65s	7B	61.7
Xfbb121b	7B	61.7
Xgpw2107	7B	61.7
wPt-7318	7B	61.8
Xfbb9b	7B	62.8
XgbxR799b	7B	62.8
wPt-2883	7B	63.3
Xutv911	7B	64.1
Xpsr129	7B	64.8
Xfba17	7B	66.5
Xfba321	7B	66.5
Xfba322	7B	66.5
Xfba93	7B	66.5
Xfbb156c	7B	66.5
Xfbb278d	7B	66.5
Xgwm471	7B	66.5
Xgwm537	7B	66.5
Xgwm601	7B	66.8
P41/M58-169	7B	67.9
wPt-7602	7B	68
Xgwm951	7B	68.5
Xgwm195	7B	69
wPt-7653b	7B	70.6
Xwmc405	7B	70.7
wPt-2737	7B	70.9
wPt-8283	7B	71.2
wPt-2914	7B	71.5
wPt-1723	7B	71.6
wPt-0800	7B	71.9
Xdupw450	7B	72.1
Xcdo475B	7B	72.3
Xgwm400	7B	72.3
Xfbb186	7B	73.3
Xfbb67a	7B	73.3
Xglk301	7B	73.3
P32/M62-5	7B	74.3
Xfba127a	7B	74.3
Xwmc76	7B	74.3

Xfba109	7B	76.3
Xopae7-337	7B	76.3
Xfba346	7B	77.3
Xfba42a	7B	77.3
Xgwm130	7B	77.3
Xgwm526	7B	77.3
Xcdo1395	7B	79.3
Xopa10-280	7B	79.3
Xpsr952	7B	79.3
Xwmc435i	7B	79.3
S18M18_11	7B	80.3
Vrn-B3	7B	80.3
Xgwm60	7B	80.3
S18M20_4	7B	81.3
Xabc158	7B	81.3
Xgwm68	7B	81.3
Xabc465	7B	81.5
Xdupw403a	7B	81.7
Xcfa2028	7B	82.4
Xfba248	7B	83.4
wPt-9467b	7B	84.2
Xubp9	7B	85.6
Xutv777	7B	85.6
Xfbb222b	7B	86.1
Xfbb226.1	7B	86.3
Xgpw2119	7B	86.3
BE499017-174	7B	87.1
Xwmc546.1	7B	87.2
Xgpw2103	7B	87.2
Xgwm935	7B	87.2
P78/M87.4	7B	88.2
Xbcd310	7B	88.2
Xgwm276	7B	88.2
Xbarc85	7B	88.2
Xutv1557	7B	88.4
P34/M48-1	7B	88.9
Xcnl8.1	7B	88.9
Xfbb150	7B	88.9
Xpsr955	7B	88.9
Xwg180a	7B	88.9
Xpsr103	7B	89.9
AL825137	7B	90.2
wPt-1457b	7B	90.4
wPt-4230	7B	90.4
Xwmc182	7B	90.5

wPt-9630	7B	90.9
Xgwm573	7B	91.4
Xutv913	7B	91.4
Xgwm573b	7B	91.5
Xwmc426	7B	91.5
Xutv1441	7B	91.6
wPt-0963	7B	91.6
Xbarc83	7B	91.7
Xbarc4b	7B	92
Xwmc376	7B	92
wPt-5463	7B	92
Xbcd1066	7B	92.2
wPt-6498	7B	92.2
Xpsr303	7B	92.4
Xbcd707	7B	92.5
Xcdo949	7B	92.5
Xgwm871	7B	92.6
Xpsr152	7B	92.6
Xubp18	7B	92.6
Xutv1268	7B	92.6
Xgwm941	7B	92.8
M67/P77.2	7B	93
Xbcd385	7B	93
Xfba32	7B	93
Xfba363b	7B	93
Xfbb195	7B	93
Xgwm46b	7B	93
wPt-4863	7B	93.1
wPt-0318	7B	93.2
P78/M71.2	7B	93.5
Xbcd1338	7B	93.5
Xfba340	7B	93.5
Xpsp3015	7B	93.5
Xwmc399	7B	93.5
Xbarc65	7B	93.5
Xbarc72	7B	93.5
Pm40	7B	94.5
Xwmc546	7B	94.5
Xwmc758	7B	94.5
Xbarc255	7B	94.5
Xbcd98	7B	94.5
Xfba371	7B	94.5
Xwmc546.2	7B	94.7
Xgwm43	7B	95.2
wPt-5737b	7B	95.2

wPt-7925	7B	95.2
Xwmc335	7B	96.2
Xabc465a	7B	96.4
Xbcd349	7B	96.4
Xpsr490.1(Ss1)	7B	96.4
Xgwm16	7B	96.4
wPt-9814b	7B	96.4
Xgwm297	7B	96.6
Xwmc475	7B	96.8
Xwmc662	7B	97.2
Xgwm644	7B	97.3
Xwmc696	7B	97.5
Xwmc364	7B	97.9
Xwmc476	7B	97.9
Xwmc471b	7B	98.1
Xbarc267	7B	98.4
Xwmc218	7B	98.7
Xabc455	7B	98.9
Xfba234b	7B	98.9
Xfbb264	7B	98.9
Xpsr94	7B	98.9
Xbarc95	7B	99
XksuH1	7B	99.2
Xutv147	7B	99.2
wPt-8106b	7B	99.2
Xcdo595	7B	99.3
Xglk549	7B	99.3
Xgwm333	7B	99.3
Xgwm963	7B	99.3
XS13M50(330)	7B	99.8
Xwmc265	7B	99.9
Xcdo17	7B	100
Xcnl7.3	7B	100
Xgbx4899	7B	100
Xgpw2264	7B	100
Xgpw2269	7B	100
Xgwm897	7B	100.5
P32/M55-1	7B	101
S12M17_1	7B	101
Xcdo551	7B	101
Xcdo962	7B	101
Xfba337	7B	101
Xfbb193a	7B	101
Xfbb218	7B	101
Xgwm540	7B	101

Xpsr690	7B	101
Xwmc269	7B	101
Xutv934	7B	101.1
Xwmc435	7B	101.1
Xbarc90	7B	102
Xfba311b	7B	102
Xfbb343x	7B	102
XgbxGx228	7B	102
Xgpw1100a	7B	102
Xgpw313b	7B	102
S12M20_1	7B	103
Xbcd178	7B	103
XgbxG732	7B	103
Xglk598	7B	103
Xopag18-340	7B	103
Xrz476	7B	103
Xwmc396	7B	104
Xwmc653	7B	104.1
wPt-6463	7B	104.6
wPt-7934b	7B	104.7
Xgwm213	7B	104.8
wPt-11310	7B	104.8
W01T03b	7B	105.2
Xpsr490	7B	105.6
wPt-3873	7B	105.7
Xutv1110	7B	106.2
XS13M50(460)	7B	106.3
wPt-0103b	7B	107.5
Dn1881	7B	108.6
Xglk478	7B	108.6
Xpsr927	7B	108.6
Xwmc17	7B	108.6
TC69176	7B	108.6
Xbarc176	7B	108.6
XksuG12	7B	109.6
Xwg514	7B	109.6
P31/M48-2	7B	110.6
Xfba354b	7B	110.6
Xfbb258	7B	110.6
XgbxG575a	7B	110.6
Xopz10-680	7B	110.6
P78/M49.2	7B	111.6
P78/M63.4	7B	111.6
S23M15_4	7B	111.6
Xfba301	7B	111.6

M62/P64.2	7B	112.6
Xfba69a	7B	112.6
Xfbb179	7B	112.6
Xgpw2083a	7B	112.6
Xpsr350	7B	112.6
S24M26_5	7B	113.6
Xbarc278	7B	113.6
Xfbb175	7B	113.6
Xgpw2092	7B	113.6
P78/M63.7	7B	114.6
Xfba97	7B	114.6
M77/P64.12a	7B	115.6
S13M18_3	7B	115.6
Xfba305	7B	115.6
Xfbb145b	7B	115.6
Xfbb18	7B	115.6
Xfbb352	7B	115.6
Xgwm274	7B	115.6
Xwg686	7B	116.6
Pc1	7B	117.6
Stb13	7B	117.6
Xfba382a	7B	117.6
Xwg180b	7B	117.6
TC69177	7B	117.6
wPt-1149	7B	117.7
XS13M49(400)	7B	118.2
XS25M49(175)	7B	118.2
XS25M49(400)	7B	118.2
wPt-11598	7B	118.7
wPt-3833	7B	118.8
S25M15_19	7B	119
Xpsr680	7B	119
wPt-2305	7B	119
wPt-0920	7B	119.1
wPt-6372	7B	119.2
CA668788a	7B	119.3
wPt-3730	7B	119.3
Xgwm1036	7B	119.4
wPt-4025	7B	119.4
wPt-8919	7B	119.4
TC95791	7B	119.5
Xpsr687	7B	119.5
Xcdo686	7B	120
Xpsr593c	7B	120
Xpwir232b	7B	120

S13M19_8	7B	122
Xgwm112	7B	122
wPt-0841	7B	122
wPt-3447	7B	122
wPt-8193	7B	122
wPt-8312	7B	122
wPt-9133	7B	122
wPt-9698	7B	122
wPt-8069	7B	122.6
wPt-0980	7B	123.3
wPt-4323	7B	123.3
Xcfa2106	7B	123.6
Xdupw403b	7B	123.6
wPt-1817	7B	123.6
wPt-6700	7B	124.2
Xcfd22	7B	124.3
wPt-1250b	7B	125.4
Xdupw395b	7B	126.4
wPt-6180	7B	126.7
Xwmc51	7B	127.4
wPt-9665	7B	128.1
wPt-8981	7B	128.3
Xgwm302	7B	128.9
wPt-9511	7B	129
wPt-3503b	7B	129.3
Xwmc540b	7B	129.7
Xwmc723	7B	129.7
Xwmc540	7B	129.9
P38/M59-224	7B	130.2
Xwmc051	7B	130.2
BJ213673a	7B	131
Xfba259	7B	131.2
wPt-6162	7B	132.9
Xgwm767	7B	133.5
wPt-9071	7B	133.5
wPt-2273	7B	134.1
wPt-2994	7B	134.4
P78/M59.7	7B	134.7
S24M18_12	7B	134.7
Xwmc517	7B	134.7
Xgwm131	7B	134.7
Xbarc315	7B	135.7
Xwmc537	7B	137.4
Xpsp3033	7B	140.7
wPt-3723	7B	141

wPt-5922	7B	141.1
wPt-1826	7B	141.6
Xgwm746	7B	142.2
Xpsr129c	7B	142.7
Xdupw398	7B	143.8
wPt-4258	7B	144.5
Xfbb67b	7B	144.6
Xopag2-950	7B	144.6
Xgwm111	7B	146.6
wPt-2688	7B	146.8
wPt-9925	7B	147.4
M77/P64.12	7B	147.6
wPt-5892	7B	147.7
wPt-7887	7B	147.9
XksuD2	7B	148.6
Xcfd6a	7B	148.8
M86/P65.0	7B	149.6
P32/M52-343	7B	149.6
Xgwm746.2	7B	149.6
wPt-1553	7B	149.6
Xwmc792	7B	149.8
M83/P65.7	7B	150.1
P42/M52-3	7B	151.1
M21/P76.9	7B	152.1
M62/P64.11	7B	152.1
M65/P64.2	7B	152.1
Xglk302	7B	152.1
tPt-5747	7B	152.5
P36/M49-6	7B	152.7
P36/M53-184	7B	152.7
P37/M57-180	7B	152.7
Xpsr547	7B	152.7
XP78M71.6	7B	153.7
Xbarc258	7B	153.8
wPt-5341	7B	154.2
M62/P64.9	7B	154.6
P35/M47-1	7B	154.6
P40/M60-58	7B	154.6
XksuE18	7B	155.6
tPt-7362	7B	156.1
wPt-0447	7B	156.3
wPt-1715	7B	156.3
wPt-2668	7B	156.3
wPt-4010	7B	156.3
wPt-4298	7B	156.3

wPt-7351	7B	156.3
P78/M43.14	7B	157.1
Xwmc311	7B	157.1
Xgwm232	7B	158
wPt-8417	7B	158
rPt-3887	7B	158.1
wPt-8615	7B	158.2
wPt-5343	7B	158.4
wPt-8921	7B	158.8
wPt-1422	7B	159.2
wPt-4342	7B	159.2
wPt-5547	7B	159.2
wPt-0482	7B	159.3
wPt-9547	7B	159.6
wPt-5280	7B	160.5
Xgwm420	7B	160.8
wPt-0194	7B	160.9
Xglk750	7B	162.1
wPt-4297	7B	163.2
Xwg232a	7B	163.7
wPt-2356	7B	165.9
Xcnl2.1	7B	166
Xgwm1267	7B	166
wPt-4814	7B	166.1
mlxbd	7B	166.4
P32/M53-4	7B	167.2
P35/M61-1	7B	167.2
Xmwg710a	7B	167.2
wPt-6156	7B	167.3
wPt-3086	7B	167.7
wPt-4300	7B	167.7
wPt-1132b	7B	167.8
wPt-8938	7B	168.3
Xgwm783	7B	169.1
Xwmc613	7B	169.6
TC88833	7B	170.3
P40/M47-4	7B	170.4
Xgwm611	7B	170.4
Xcnl1	7B	171.4
Xwg420	7B	171.4
P35/M47-3	7B	172.4
P38/M49-1	7B	172.4
P41/M48-98	7B	172.4
P41/M50-8	7B	172.4
Xcdo414	7B	172.4

Xwmc322	7B	172.4
P39/M53-3	7B	173.4
Psy-B1	7B	173.4
Xglk576	7B	173.4
Pm5d	7B	173.9
Lr19	7B	174.4
P33/M55-6	7B	174.4
P35/M49-5	7B	174.4
P38/M54-3	7B	174.4
Rht13	7B	174.4
Sr25	7B	174.4
Xfba21	7B	174.4
Xfbb189.1	7B	174.4
Xgwm577	7B	174.4
wPt-3190	7B	174.4
wPt-7740	7B	174.4
wPt-3093	7B	175.1
wPt-0600	7B	176.5
PmTm4	7B	177.3
wPt-8040b	7B	178.6
wPt-3058	7B	178.8
wPt-6104	7B	178.9
wPt-8989	7B	178.9
wPt-9880	7B	178.9
wPt-0217	7B	179
wPt-7295	7B	179
wPt-1267	7B	179.2
wPt-3939	7B	180
XpdaC01	7B	180.3
wPt-8007	7B	180.3
Xmag600	7B	181.1
Xwmc581	7B	181.3
wPt-5138	7B	183.1
wPt-6701	7B	184.5
wPt-6657	7B	185.8
wPt-6869b	7B	186.1
wPt-1075	7B	186.5
wPt-1957	7B	186.5
wPt-5228	7B	186.5
wPt-5462	7B	186.5
Pm5e	7B	186.8
wPt-7413	7B	187
Xbarc10	7B	187.2
Xbarc1073	7B	187.3
wPt-7720	7B	187.4

wPt-3530	7B	188.1
wPt-4743	7B	188.2
wPt-5585	7B	188.2
Swes619	7B	188.4
wPt-3439b	7B	188.4
wPt-7219b	7B	188.4
wPt-7374	7B	189.9
Xgwm344a	7B	190.2
wPt-2677	7B	190.4
Xbarc340	7B	190.7
Xpsr680b	7B	191.1
wPt-6484	7B	191.3
wPt-4054	7B	191.5
Xmag4362	7B	191.6
XgbxR035	7B	192.1
wPt-5309	7B	192.6
wPt-7046	7B	192.7
Xwmc166	7B	192.9
Xwmc276	7B	193
Xbarc32	7B	193.1
Xbarc50	7B	193.3
wPt-4038	7B	193.8
tPt-6363	7B	194.1
wPt-3004	7B	194.3
wPt-2449	7B	194.8
wPt-4393	7B	195
wPt-7108	7B	195
wPt-5617	7B	195.2
Xwmc273	7B	196.1
TC70722	7B	196.2
Xmwg2062	7B	196.7
Xmag4321	7B	197.3
wPt-4902	7B	197.7
Xcfa2040	7B	198.4
wPt-4140	7B	198.5
wPt-4875	7B	198.5
wPt-5747	7B	198.5
Xbarc182	7B	199
Xgwm1061	7B	199.3
wPt-3710	7B	199.6
wPt-0494	7B	200.1
wPt-3785	7B	200.1
wPt-5816	7B	200.1
wPt-6276b	7B	200.1
wPt-6320	7B	200.1

wPt-9813	7B	200.1
tPt-8504	7B	200.2
wPt-0126	7B	200.2
wPt-1359	7B	200.2
wPt-2838	7B	200.2
wPt-4319b	7B	200.2
wPt-4644	7B	200.2
wPt-5677	7B	200.2
wPt-8936	7B	200.2
wPt-9515	7B	200.2
wPt-9746	7B	200.2
wPt-9992	7B	200.2
wPt-0577	7B	200.3
wPt-4620b	7B	200.4
wPt-9877	7B	200.6
wPt-2878	7B	200.7
wPt-3402	7B	200.7
wPt-5377	7B	200.7
Xmag1715	7B	201.1
Xmag1932	7B	201.1
Xmag1933	7B	202.3
wPt-1475	7B	202.6
BG262689-071	7B	203.1
wPt-7891	7B	204.3
wPt-8598	7B	204.5
Xwmc557	7B	204.9
wPt-1085	7B	204.9
wPt-0504	7B	206
wPt-6869d	7B	206
wPt-5069	7B	206.7
Xwmc10	7B	207.9
Xwmc526	7B	209.5
Xwmc500	7B	209.8
wPt-8650c	7B	209.9
wPt-4057	7B	210.6
wPt-4259	7B	210.6
wPt-7567b	7B	210.6
Xbarc123	7B	211
Xwmc70	7B	211.1
Xgwm146	7B	212.4
QLr.osu-7BL	7B	213.4
wPt-0465	7B	214.1
Stb8	7B	214.4
Xrz508	7B	214.4
wPt-1069	7B	214.8

Xgwm344b	7B	216.4
wPt-0786	7B	217.4
Xwmc232	7B	218.8
Xbarc94	7B	220.4
Xbarc20	7B	220.8
Xwmc261	7B	231.8
wPt-9488	7B	238
Xmwg710b	7B	272.6
Xgwm350	7D	0
Xwmc646	7D	6
Xfba243	7D	10
Xgpw1108c	7D	10
XgbxR799a	7D	13
wPt-0833b	7D	16.3
Xbcd1975	7D	17
Xwmc506	7D	21
D1.1_ctg1720	7D	22
Xpsr103b	7D	22
wPt-2551	7D	24.1
wPt-0992b	7D	24.7
Xbcd129	7D	27
Xbarc184	7D	28.1
Xwmc450	7D	30.8
Xgwm635	7D	31.5
D1.1_ctg1058	7D	32.2
Xcdo1400	7D	32.2
Xbarc70	7D	41.3
Xbcd588	7D	42.2
Xcfd41	7D	44.2
Xgpw328	7D	46.2
Xgpw1142a	7D	50.2
Xgpw351	7D	51.2
Xgdm145	7D	52.3
Xgdm88	7D	52.3
Xcfd31	7D	53.2
Xcfd26	7D	53.4
Xfba253	7D	53.4
Xcnl6a	7D	54.4
Xcfd66	7D	55.1
Xwmc629	7D	55.3
D1.1_ctg4058	7D	55.5
D1.1_ctg4364	7D	55.5
Xbcd1872	7D	55.5
Xwmc698	7D	56
Xwmc606	7D	57.3

D1.1_ctg3072	7D	58.4
D1.1_ctg4968	7D	58.4
D1.1_ctg8047	7D	58.4
D1.1_ctg9710	7D	58.4
Xfba8y	7D	58.4
XgbxG564	7D	60.4
Xgwm130	7D	61.2
Xbarc154	7D	64.5
Xcfd30	7D	67.1
D1.1_ctg1525	7D	67.1
D1.1_ctg5098	7D	67.1
Xwg834	7D	67.1
Xbarc92	7D	68.1
Xwmc827	7D	68.2
BE418437-195	7D	70.9
Xbarc87	7D	71.9
Xbarc5	7D	72.6
Ltn	7D	73.2
Yr18	7D	73.3
Xgpw1106	7D	73.9
Xgwm1220	7D	74.3
Xwmc463	7D	74.6
Xswm10	7D	74.6
Lr34	7D	76.6
Xwmc405	7D	76.6
Xbcd1438	7D	78.6
Xbarc126	7D	79.6
Xgpw1142b	7D	80.5
Xgwm295	7D	81.4
Xgwm44	7D	82.9
Xcnl6b	7D	83.3
Xbarc352	7D	86.3
D1.1_ctg3701	7D	87.3
D1.1_ctg4161	7D	87.3
Xmwg710b	7D	87.3
Xcfd21	7D	88.3
Xrz2	7D	97.3
Xwmc438	7D	99.1
Xwmc702	7D	99.1
Xfbb366b	7D	100.1
Xbarc125	7D	101.1
Stb5	7D	103.1
Xfba377	7D	108.1
Xgpw299	7D	108.1
Xcfd46	7D	109.1

XksuA1a	7D	109.1
Xbarc214	7D	117.1
Xcfa2174	7D	122.1
Xgwm676	7D	122.1
Xwmc121	7D	124.1
D1.1_ctg4396	7D	125.1
M62/P64.6	7D	125.1
P78/M43.5	7D	125.1
Stb4	7D	125.1
Xpsr160.2	7D	125.1
Xpsr392.2	7D	125.1
Xpsr563.1	7D	125.1
Xwmc42	7D	125.4
Xgwm111	7D	126.5
XWC0031du	7D	126.9
Xbarc128	7D	126.9
Xgwm296	7D	127.9
Xwmc414	7D	127.9
XWB154u	7D	128.9
Xcfd68	7D	128.9
Xmgl59	7D	128.9
Xpsp3035	7D	129.9
XWB026u	7D	130.9
XWB128ib	7D	130.9
XWC0014u	7D	130.9
Xpsp3113	7D	130.9
Xwmc017	7D	130.9
Xgwm473	7D	131.2
Xwmc653	7D	131.2
Xcfd2	7D	132.2
Xwmc182	7D	132.2
P34/M50-14	7D	132.8
Xgwm437	7D	132.8
Xwmc489	7D	132.8
Xwmc630	7D	132.9
Xwmc221	7D	133
S24M20_11	7D	134
Xbarc26	7D	134
Xcfd14	7D	134
Xcfa2099	7D	139
Xfbb112	7D	142
Xwmc473	7D	142.3
Xcfd193	7D	143.4
Xgpw2052	7D	144.7
Xgpw350	7D	144.7

Xgpw2160	7D	145.7
Xwmc488	7D	145.9
D1.1_ctg7915	7D	147.5
Xcdo775	7D	147.5
S15M15_1	7D	148.5
Xwg719	7D	148.5
Xwmc426	7D	149.5
Xbarc172	7D	151.5
Xgwm121	7D	154.4
Xwmc94	7D	154.5
Xwmc150	7D	157.8
Xbarc121	7D	159.3
Xgdm67	7D	159.6
Xabc173	7D	160.2
Xwmc797	7D	160.6
Xgdm46	7D	162.1
Xcfd25	7D	164.1
Xbarc105	7D	167.1
Xgpw2327	7D	169.1
XksuE9	7D	169.1
Xwmc671	7D	171.5
Xbarc111	7D	171.8
Xgpw1009	7D	171.8
Xfba69.2	7D	173.8
XgbxG451	7D	174.8
Xgpw334	7D	174.8
Xpsp3123	7D	174.8
D1.1_ctg10053	7D	178.8
Xfbb79	7D	178.8
D1.1_ctg4224	7D	179.8
Xfba264	7D	179.8
D1.1_ctg11444	7D	184.8
Xmwg975	7D	184.8
Xpsp3045	7D	187.8
Xfbb325	7D	188.8
Xwmc824	7D	190
XgbxG411c	7D	193.5
Xbarc53	7D	196.4
Xwg420	7D	197.2
Gb7	7D	199.2
Xbarc235	7D	199.2
Xfba204a	7D	202.2
Xgwm428	7D	206.2
Xpsp3094.2	7D	210.2
Gbd	7D	219.2

Xfbb189.2	7D	220.2
Gbz	7D	221.2
Xwmc157	7D	221.2
Gbx1	7D	224.2
Gb3	7D	235.2
Xgwm37	7D	235.2
Xwmc273	7D	237
Xwmc634	7D	237.3
Bdv2	7D	237.4
Xcfa2040	7D	238.2
M71/P77.3	7D	238.3
Xbarc76	7D	238.4
Xcfd69	7D	239.1
Xwmc166	7D	241
M71/P77.7	7D	244.3
D1.1_ctg5649	7D	245.3
XksuE3.4	7D	245.3
Xwmc14	7D	247
Xcfd175	7D	248.4

Supplementary material 2. Description and category of the traits collected for QTL meta-analysis.

Trait	Description	Category
ACT	Root Activity	Root Activity
ARD	Average Root Diameter	Root Diameter
ARL	Average Root Length	Root Length
ARN	Axial Root Number	Root Number
AWSDW	Above-wax seminal dry weight	Root Weigth
BWSDW	Below-wax seminal dry weight	Root Weigth
CCR	Root Ca2+ concentration	Nutrient Concentration
CRS	Number of Root Crossings	Root Crossings
DRR	Deep Root Ratio	Root Depth
DRW	Deep Root Weight	Root Weigth
EASR	Emergence Angle of the Seminal Root	Root Angle
FRK	Number of Root Forks	Root Number
KCR	Root K+ concentration	Nutrient Concentration
LatRL	lateral root length	Root Length
LRL	Longest Root Length	Root Length
LRN	Number of lateral roots	Root Number
MaxRD	Maxium Root Depth	Root Depth
MaxRL	Maximal Root Length	Root Length
MaxRW	Maximum Root Width	Root Width
MCR	Root Mg2+ concentration	Nutrient Concentration
MRL	Main Root Length	Root Length
NCR	Root Na+ concentration	Nutrient Concentration
PRA	Project Root Area	Root Area
PRD	Primary Root Diameter	Root Diameter
PRE	Primary root elongation	Root Length
PRL	Primary Root Length	Root Length
PRS	Primary Root Surface	Root Area
PRV	Primary Root Volume	Root Volume
RAH	Root Angle in response to Hydrotropism	Root Angle
RBB30	Root biomas below 30cm	Root Biomass
RBU30	Root biomass up to 30cm	Root Biomass
RDW	Root Dry Weight	Root Weigth
RER	Root Elongation Rate	Root Length
RFW	Root Fresh Weight	Root Weigth
RGA	Root Growth Angle	Root Angle
RHL	Root Hair Length	Root Length
RKC	Root K content per plant	Nutrient Concentration
RKUE	Root K utilization efficiency	Nutrient Concentration
RNC	Root N content per plant	Nutrient Concentration
RP	Root Penetration	Root Root Growth
RPC	Root P content per plant	Nutrient Concentration
RPR	Root-Plant biomass Ratio	Root Biomass

RPUE	Root P utilization efficiency	Nutrient Concentration
RRGI	Relative Root Growth Inhibition	Root Growth
RSA	Root surface Area	Root Area
RSR	Root Shoot Ratio	Root Shoot Ratio
RT6	Presence of the sixth asymmetric seminal root	Root Number
RV	Root Volume	Root Volume
SAL	Seminal Axes Length	Root Length
SASA	Seminal Axes Surface Area	Root Area
SAVol	Seminal Axes Volume	Root Volume
SLL	Seminal Lateral roots Length	Root Length
SLSA	Seminal Lateral roots Surface Area	Root Area
SLVol	SeminalLateral roots Volume	Root Volume
SRA	Seminal Root Angle	Root Angle
SRL	Seminal Root Length	Root Length
SRN	Seminal Root Number	Root Number
SRW	Shallow Root Weight	Root Weigth
TIP	Number of Root Tips	Root Number
TRB	Total Root Biomass	Root Biomass
TRD	Total Root Diameter	Root Diameter
TRL	Total Root Length	Root Length
TRL30	Total Root Longer 30cm	Root Length
TRN	Total Root Number	Root Number

Supplementary Material 3. QTL overview index. The horizontal solid line represents the average value for the overview index and the horizontal dotted line represents the threshold for high values.

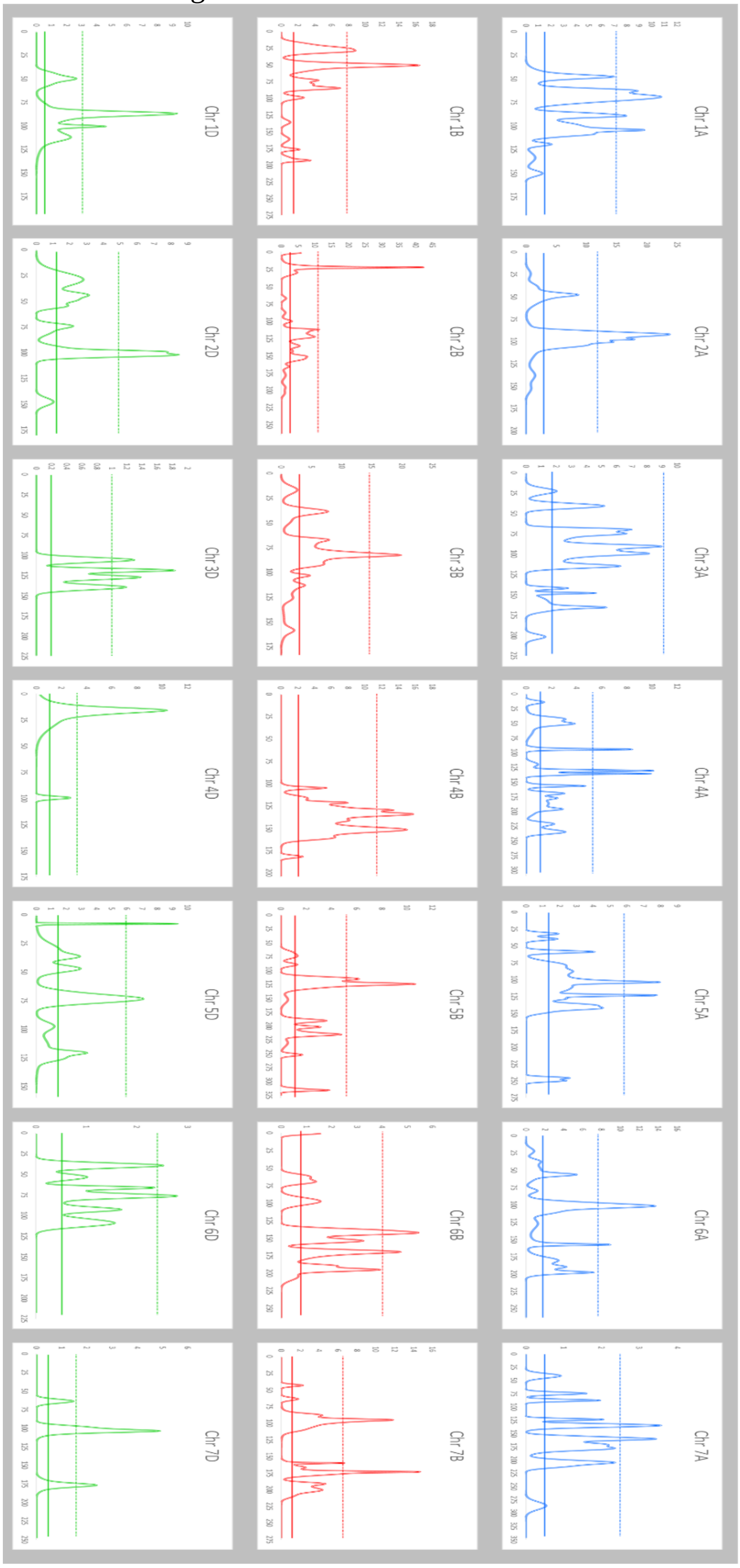

Supplement: Supplementary file 1 — Supplementary Info [file 41598_2019_47038_MOESM1_ESM.pdf]
